# Care for older adults with disabilities in Long Term Care Facility

**DOI:** 10.1590/0034-7167-2022-0767

**Published:** 2023-12-08

**Authors:** Isadora Queiroz Correa Garchet Furtado, Isabela Silva Cancio Velloso, Carolina Salles Galdino, Alexandre de Pádua Carrieri

**Affiliations:** I Universidade Federal de Minas Gerais. Belo Horizonte, Minas Gerais, Brazil

**Keywords:** Aged, Frail Elderly, Health of Institutionalized Elderly, Homes for the Aged, Nursing Care, Anciano, Anciano Frágil, Salud del Anciano Institucionalizado, Hogares para Ancianos, Atención de Enfermería, Idoso, Idoso Fragilizado, Saúde do Idoso Institucionalizado, Instituição de Longa Permanência para Idosos, Cuidados de Enfermagem

## Abstract

**Objective:**

to analyze the constitution of care offered to older adults with disabilities, from Long Term Care Facility professionals’ perspective.

**Methods:**

this is a discourse analysis based on the post-structuralist framework. Participants in this study are professionals involved in the care for older adults, totaling 14 respondents, 13 women and one man.

**Results:**

from professionals’ perspective, there is a fine line between caring for older adults with disabilities and maintaining their autonomy. Care in which autonomy is restricted predisposes older adults to a process of dependency.

**Final considerations:**

caring for older adults with disabilities constitutes the challenge between caring and maintaining independence. Thus, older adult-centered care should be taken as a premise so that their individualities are respected.

## RESUMO

**Objetivo:**

analisar a constituição do cuidado ofertado à pessoa idosa com incapacidades, na perspectiva de profissionais de uma Instituição de Longa Permanência para Idosos.

**Métodos:**

trata-se de uma análise do discurso com base no referencial pós-estruturalista. Os participantes deste estudo são profissionais envolvidos no cuidado de pessoas idosas, totalizando 14 entrevistados, sendo 13 mulheres e um homem.

**Resultados:**

na perspectiva dos profissionais, existe uma linha tênue entre o cuidar da pessoa idosa com incapacidades e a manutenção da sua autonomia. O cuidado em que a autonomia é restrita predispõe ao processo de dependência da pessoa idosa.

**Considerações finais:**

a cuidado à pessoa idosa com incapacidades se constitui o desafio entre cuidar e manter a independência. Assim, deve-se ter como premissa o cuidado centrado na pessoa idosa, para que suas individualidades sejam respeitadas.

## RESUMEN

**Objetivo:**

analizar la constitución del cuidado ofrecido a los ancianos con discapacidad, en la perspectiva de los profesionales de una Institución de Larga Estancia para Ancianos.

**Métodos:**

se trata de un análisis del discurso basado en el marco postestructuralista. Los participantes de este estudio son profesionales involucrados en el cuidado de personas mayores, con un total de 14 encuestados, 13 mujeres y un hombre.

**Resultados:**

desde la perspectiva de los profesionales, existe una delgada línea entre cuidar a los ancianos con discapacidad y mantener su autonomía. Los cuidados en los que se restringe la autonomía predisponen al anciano a un proceso de dependencia.

**Consideraciones finales:**

el cuidado del anciano con discapacidad constituye el desafío entre cuidar y mantener la independencia. Así, debe tomarse como premisa el cuidado centrado en el anciano, de modo que se respeten sus individualidades.

## INTRODUCTION

The number of older adults in the world has been increasing continuously and exponentially. In 1950, there were 202 million people over 60 years old, increasing to 1.1 billion in 2020, and should reach 3.1 billion in 2100. The older adult population aged 60 years or over represented 8% of the total population in 1950, increasing to 13.5% in 2020, and should reach 28.2% in 2100. The increase in older adults in Brazil has been faster when compared to the global scenario. The number of Brazilian older adults aged 60 and over was 2.6 million in 1950, rising to 29.9 million in 2020, and should reach 72.4 million in 2100. Older adults aged 60 and over accounted for 4.9% of the total population in 1950, rising to 14% in 2020, and should reach the impressive percentage of 40.1% in 2100^(1)^.

This scenario of growth in older adults and also in the longevity of individuals, concomitant with socioeconomic, cultural and family structure changes, has led to an increase in demand for vacancies in Long Term Care Facility (LTCF)^(2)^. LTCF are defined as “governmental or non-governmental institutions, of a residential nature, intended for the collective residence of people aged 60 or over, with or without family support, in a condition of freedom, dignity and citizenship”^(3)^.

LTCF’s main characteristic is to defend older adults’ dignity and rights, but this is questioned due to the fact that many of them resemble large accommodation, marked by strict rules, predetermined routines and the absence of perspectives for residents. In this context, individual differences and the life stories of each individual are ignored. Thus, older adults tend to lose their identity and autonomy, becoming a passive subject, which can lead to isolation and make them apathetic and even depressed^(4)^.

The natural process of aging does not necessarily involve the presence of functional decline, however, when it does occur, it can be attributed to the disabilities that affect older adults. Thus, the impairment of the main functional systems generates disabilities, such as cognitive impairment, postural instability, immobility, sphincter incontinence, iatrogenesis, family failure and communicative disability^(5)^. The presence of these disabilities in older adults’ lives makes them more dependent.

Functional decline in older adults should be analyzed from two perspectives: one related to cognition and one related to physical limitations. Regarding cognition, it comprises the mental sphere and the ability to store, process information and constitute memory^(6)^. Regarding physical limitations, Resolution 502/2021 of the Brazilian National Health Regulatory Agency (*Agência Nacional de Vigilância Sanitária*)^(3)^ defines older adults’ degree of dependency, and this dependency is characterized as a condition of individuals who require the help of people or special equipment to carry out activities of daily living (ADL).

The option for institutionalizing older adults who require, to some extent, support from another person to carry out their ADLs as well as the option to keep them in their own home or that of a family member is related to care that will be necessary and possible to be provided to them. Care demands responsibilities, and thinking about the attributions of this care involves deciding who will care, how and why, determining how the social context will be organized for this. It is through this organization that the possibilities, impossibilities and limits that shape care practices and relationships are delimited^(7)^.

Success in practices and in the action of treating to care permeates understanding older adults’ institutionalization process and how this interferes with the transforming action of their identity. Thus, care routines become an attribute of being for intersub-jectivity. All this dynamism raises the need for a projected and molded construction so that the assistance does not only have a practical north, but an ethical, affective and aesthetic orientation^(8)^.

In LTCF, it is important that the care offered to older adults is carried out by qualified professionals^(3)^ and that the practice of this care is widely discussed and reflected by society. However, what has been seen is the participation of some entities in the elaboration of documents that guide the care provided to the institutionalized older adult population, narrowing the possibilities of discussions with society. This fact is legitimized due to the incipient implementation of public policies in Brazil for this group, recognizing that studies are needed to identify sensitive points that directly interfere with care in LTCF^(9)^.

Older adults’ institutionalization scenario is questioned when there is a restriction of their autonomy as a result of cognitive loss and loss of independence. This justifies the relevance of investigating, from different perspectives, what are the challenges of care considering the development of disabilities by older adults in LTCF.

## OBJECTIVE

To analyze the constitution of care offered to older adults with disabilities, from professionals’ perspective in a Long Term Care Facility.

## METHOD

### Ethical aspects

In order to meet the ethical aspects, the project was submitted for approval by the Ethics and Research Committees of the *Universidade Federal de Minas Gerais* (COEP/UFMG). LTCF’s acceptance to participate in the research was documented by signing a Letter of Consent. Thus, the specifications of Resolution 466, of December 12, 2012, and Resolution 510, of April 7, 2016, of the Brazilian National Health Council are accepted. The Informed Consent Form (ICF) was obtained from all individuals involved in the research, through 2 copies. One copy was delivered to the interviewee at the time of the interview, and the other was filed by the responsible researcher in an appropriate place.

### Study design

This is a qualitative study, based on discourse analysis (DA), based on the post-structuralist theoretical-philosophical framework, which has French philosopher Michel Foucault as one of its main representatives. Qualitative research explores the non-quantified, emphasizing the dynamics of the meanings of human actions and relationships, which corresponds to a deepening of phenomena, processes and relationships^(10)^. The results of this research followed the COnsolidated criteria for REporting Qualitative research (COREQ) guidelines.

### Study setting

The chosen LTCF is philanthropic, and is located in the Center-South Region of the capital of Minas Gerais, and offers 30 vacancies, characterized by being an institution exclusively for older adult women. The institution has a multidisciplinary care team composed of nurses, social workers, physicians, psychologists, caregivers and nursing technicians.

### Methodological procedures

A semi-structured interview was applied, which contained the following questions: what is it like, for you, to work in an LTCF? How is your daily work at the institution? Do you consider that the older adults in this LTCF are able to make decisions about the things they need to do in their daily routine? Why? Could you tell me a little about your relationship with the older adults who live here? What is your perception about the relationship of institutionalized older adults with their family and friends? Do you care for institutionalized older adults? What kind of care do you provide? Do you consider that the older adults in this LTCF are able to make decisions about the things they need to do in their daily routine? Why? In your opinion, how do the institution’s older adults and professionals deal with inmates who require palliative care? What feeling does this awaken in you? What does it mean to you to grow old and be an older adult in Brazil?

Frequent visits were made to the institution, 3 to 5 times a week, from January to March 2017, for data collection, with a record in a field diary for observation. The diary was filled in at relevant times during the interviews and together with the researchers’ observation, which took place on weekdays and weekends, alternating the hours of the day from 07:00 to 20:00. Thus, interviews were carried out in all work shifts, morning, afternoon and night, and professionals from different categories participated in the interview.

### Data source

Participants in this study were members of the body of professionals involved in care in a LTCF, and were informed about the link that the researchers had with a federal university in the state of Minas Gerais and in relation to the research topic to be developed. Inclusion criteria were defined as professionals working at the institution for three months or more, expressing a desire to participate in the research and allowing the interview to be recorded. One professional was excluded for working at the institution for less than three months.

To define the sample, the data saturation criterion was used, which consists of the technique of suspending new participants in the collection, when this inclusion generates redundancy of information and the collected data becomes repetitive^(11)^. Thus, the sample consisted of 14 professionals from the following categories: nurse, physician, psychologist, social worker, caregiver and nursing technician.

The LTCF work team was composed of a team of direct care for older adults, namely: two general practitioners, a nurse, 3 technicians and 5 caregivers. There was also the indirect care team that was responsible for management, which had a social worker and a psychologist.

### Data collection and organization

The research team consisted of three researchers, one of them with a PhD in nursing, a master’s student and a nursing student. The undergraduate student was guided and supervised by nurses in the activities in which she participated. The master’s student and the undergraduate student were trained by a professor in terms of techniques for conducting the interviews.

The researchers invited professionals to participate in the interview during their visits to the institution, scheduling a day and time that was convenient for participants. About five visits were made to LTCF to learn about the institution’s interest in carrying out the research, the house structure, the older adults and the professionals, in addition to understanding the hours of greatest demand for care by professionals and their openness to the researched. No pilot project was carried out, and the surveys were unique and were not redone.

The interviews took place in a reserved and welcoming environment in the LTCF itself, which was similar to a living room, with only the researchers, master’s and undergraduate students, and the interviewed professional present. They had an average duration of 15 minutes, being recorded in Media Player equipment and, later, the content was transcribed, in full, by the master’s or undergraduate student. There was no need to redo any interviews, nor were they returned for corrections or comments. All collected material was converted and treated as text. The transcription of the interviews into narrative texts was done in Microsoft Word as well as field notes became observation texts of care practices observed in the LTCF.

### Data analysis

The collected data were submitted to DA, which aims to define and organize the corpus, identifying the enunciating subjects. The collection of records continued, which is all linguistic data collected by the researcher in its raw state. Subsequently, the analysis was carried out, in which the data were organized, which means handling them theoretically and questioning them. In this phase, the theory of the collected data was interconnected with the researched theoretical subsidy. Then, he returned to the corpus to highlight speech characteristics, in addition to cutting and analyzing it. From the corpus, semantic groups were organized to then start DA interpretation and writing^(12)^.

It is important to emphasize that the article also addresses the constitution of care for older adults with functional disability in LTCF, although other themes were found in the analysis. After data collection, the importance of the research results was discussed with the unit manager and some participants, which will be presented to the other participants, enabling developments about this care.

## RESULTS

In the present study, 14 interviews were conducted with LTCF professionals, consisting of one man and 13 women aged between 26 and 65 years, with working time at the institution ranging from one to 30 years, five of whom had higher education and eight, secondary education. The only professionals who had specific training in caring for older adults were the team of caregivers, and the rest of the multidisciplinary team did not have training for the public in question. The interviews were identified by the letter P, followed by an Arabic numeral corresponding to the sequence of the interview.

From professionals’ perspective, the presence of physical dependency is directly related to cognitive decline, with a fine line between caring for others and maintaining their independence.

*These* [older adults residing in LTCF] *grade I, that we talk about, are the ones that walk. The ones who manage to fully manage their lives.* (P4)

*I say that those that answer for themselves, yes. The others, who have no way of answering, that it’s a habit for us. Like I say a question, who is in a wheelchair, we have to help.* (P10)

*But others, who are really lucid, have more authority, right?! Of which the ones that are so bedridden.* (P14)

The speeches also point out that independence is confused with cognitive capacity, and limits of care are established by the institution for those who depend on some type of assistance.

*Some are aware. Those who are aware* [...], *nothing prevents them from making decisions. Mainly that if we take this away from those who are able to do it, to exercise* [autonomy], *we are taking away the person’s own freedom and that, sometimes, I think it weighs heavily on LTCF.* (P5)

*Sometimes they talk, because even the non-lucid ones sometimes have moments of lucidity, when the answer is congruent with the question, right?!* (P12)

In the fragments above, the division of two categories can be observed: those who manage to manage their lives and those who cannot manage their lives. In addition to this, although there is concern about the quality of care to be provided in LTCF, sometimes this care predisposes to a process of increased dependency on older adults, especially if there is any limitation due to the lack of encouragement for independence.

*Just today, I was talking to a caregiver... about a resident who arrived here, she was getting out of bed* [...]. *She went from chair to bed, in her house, alone. Today, the woman is no longer able to leave the chair and go to bed. Why? “It will fall!” “It will hurt!” Then later, it will fall on me. Then, “I’ll put you to bed, okay?!” I’m going to bed. But I thought she regressed a lot after she got here.* [...] *today, she can barely pick up a spoon, right?!* (P12)

Again, in this fragment above, we are faced with the process of managing older adults. It is only now that caregivers’ action on older adults’ daily management leads them to lose autonomy over their daily lives. This capacity can be observed in older adults’ cognitive capacity, which is directly linked to their independence in carrying out ADLs, as can be seen in the fragment below:

*One day, I went to visit an older adult in her room and, after a few minutes of conversation, she wanted to go to the bathroom, but I noticed that she was wearing a diaper, which allowed her to urinate. However, I noticed that she did not want to urinate in the diaper, as she stated that she was afraid of “leakage” and thus getting dirty. Despite being an older adult with preserved cognitive skills, she was blind, and therefore depended on help in some daily activities, such as going to the bathroom. This older adult had recurrent episodes of urinary tract infection, which led me to reflect on the situation. The possible causes of infections may be due to the fact that the older adult did not have the independence to go to the bathroom alone or because she held her urine for a long time for fear of getting dirty, which ended up increasing the frequency of infections. In addition, to facilitate handling, the care team put on a diaper, causing the older adult to spend a longer time with this dirty, wet and uncomfortable device.* (Field Diary, 2018)

The speech that follows reinforces older adults’ dependency as a complex issue to be handled in the context of the care provided in the LTCF. Additionally, it points out that the need for help with ADL means that older adults with some degree of dependency do not have many opportunities to exercise their independence.

*There are reports here that surprise me! From a resident who came in here with sphincter control, with urinary control and who uses a diaper today, because the routine does not allow her to go to the bathroom, whenever she wants. This is serious for me! Did you understand?! What am I telling you? That she is in a wheelchair. So, to go to the bathroom, she depends on someone. That’s cruel of you to think! That you’re going to start wearing a diaper, because you can’t go to the bathroom when you want to. Cruel! When I arrived, it killed me! But, honestly speaking, there will be days when there will be no way* [decreases voice tone]. *Did you understand?!* (P7)

In a routine in which, not infrequently, professionals deal with an overload of activities, care is sometimes carried out in an impersonal way, and follows strict institutional norms, with little room for flexibility and sensitivity to listen to the person’s choices. The growing service needs to be executed and standardized by caregivers as in a Fordist assembly line and, as in every treadmill on the line, it is the time that regulates product assembly: the older adults. The following story is also an example of this.

*That the house had it like this. Oh! Let’s take! Today is the day to take so-and-so to bathe. Then, I remember that the girls came up to me and said, “Can we proceed as we did?”, “Get the so-and-so to bathe her.”, “Wow, let me see how this is?” I do not forget this scene* [...]. *Then, there was the resident taking a shower, then two caregivers came, a technician and a caregiver, they opened the door, locked it and that war scene started in there, right?! I could only hear, “Ahhh* [imitates screams and screams]”. *And I was like, “What is this?” Look at the detail, this resident is very forgetful, she stayed behind me the whole day. You sent it, right?! All day! So, what was traumatizing for her, she didn’t forget, she kept it in her memory, the whole day and for days. Then we start thinking about other solutions. We never took her by force again. Never! It has been at least eight months since this has happened.* (P7)

Thus, it is perceived that the rigid and impersonal routine in the organization of care for the other is a factor of embarrassment and dissatisfaction not only for the person being cared for, but for those who provide this care.

## DISCUSSION

Since aging is a cumulative and irreversible process, although not pathological, the body undergoes degradation, which often disables individual functionally and cognitively. Associated with this, institutionalization in LTCF leads older adults to a more sedentary life, with loss of their autonomy and gradual worsening of cognitive capacity. Their functional capacity is influenced by cognitive deficit, with damage and their independence and autonomy^(13)^.

Encouraging autonomy and independence in older adults significantly improves their quality of life. Inserting educational institutions that carry out activities with older adults helps them to exercise and socialize, a fact that already happened in the house, however, because it was a school vacation period, the house was empty. Another possibility is the insertion of the community in older adults’ lives, such as groups belonging to the church close to the house, or the residents of the surroundings, allowing visits and carrying out recreational activities, or even taking responsibility for older adults and taking them for a walk or to attend mass. The inclusion of older adults in community life contributes to their self-esteem, independence and autonomy, favoring bonds, interactions and preventing illness.

In a LTCF, setting of this study, it was observed, through the speeches, a classification of older adults according to the degree of dependency, informally standardized in the institution. This classification involved the demand for help in self-care activities, such as bathing, feeding, walking, being visually impaired, using a wheelchair or having a cognitive deficit, which were factors that led them to be classified as dependent older adults and, consequently, considered as not lucid. Due to this fragility, their decision-making power was curtailed by the team and they were subject to the power of those who cared for them. On the other hand, if older adults were autonomous, to the point of coming and going alone and deciding for themselves, they were considered independent older adults and had their autonomy protected.

The characteristics and limitations of each group of older adults defined the behaviors, demands and management in relation to the care provided to them. However, the habit of classifying reinforced the idea that, in LTCF, there were only two groups of older adults: dependent and independent. According to Foucault^(14)^, there is a need to qualify behaviors and performances based on opposing values, using a perspective between the positive and negative pole, good and bad. The imperative to qualify older adults made the unspoken, through institutional rules and norms, indispensable. Thus, although there was no formal delimitation of groups, this fact was perceived and said in speeches and in care.

The categorization of older adults, between dependent and independent, was related to performing their ADLs, and, in relation to independent older adults and without cognitive decline, few limitations were imposed. To the others, rules and limits previously established by the institution were imposed. This ideology takes us back to Foucault and his thoughts regarding disciplines in a hierarchical and disciplining environment, such as an LTCF. Thus, according to Foucault^(15)^, disciplines have their own discourse and, for that, inventively create their apparatus of know-how and knowledge. The discourse that the disciplines create is linked to the rule, to the discourse of the norm, defining a standardization code that encompasses the field of human sciences and clinical knowledge^(16)^. In this way, the institution’s knowledge disqualified other knowledge, establishing discourses and daily care practices that were convenient for them, defining which ones had or did not have autonomy.

The categorization of older adults was done at random, following parameters informally pre-established by caregivers, between managers and non-managers of their routines. It is interesting to note that the word “manage” brings responsibility and authority to it. Management, according to Chauí^(17)^, is linked to the neoliberal discourse and the competence discourse. Thus, for the professionals interviewed, the older adults who have the competence to take care of themselves are separated from the non-competent ones, and these must be managed by them, as they do not have the lucidity to do so.

The professionals who took care for older adults directly established their own criteria and standards and, as a result, each older adult received different treatment from the professional who provided care. Moreover, this informal classification functioned as a way of standardizing older adults, which aimed to homogenize, classify and hierarchize. This standardization, as already mentioned, creates the idea of a Fordist assembly line of work in LTCF, in which time is the most important category of care, bath time, food time, medicine time, time to watch television, time to stay on the patio, time to pray and time to sunbathe. It is also important to point out that classifying, hierarchizing and distributing are used by the disciplinary power as a way of normalizing a social body and, based on this homogenization, which is the rule, imperiously introduces the measurement of individual differences^(14, 15, 16, 17, 18)^.

It is understood that reality is constructed based on the assumption that there is only one universal body considered normal, and bodies that do not meet standards of normality will always be problematic and particular as well as the classifications that disable or incapacitate them, making them visible. Thus, normalization comes as a way of reproducing differences and asymmetries and, thus, fails to be successful, as it seeks to undo these distinctions. In the same perspective, policies and social services are found, as they are based on the principle of normalization, as if all bodies were normal^(19)^.

Although there are instruments that define older adults’ degrees of dependency and cognitive capacity, their systematic use was not observed in LTCF. As an example, Collegiate Board Resolution 502/2021^(3)^ defines older adults’ degree of dependency: degree I older adults are independent, even if they require using self-help equipment; those in degree II are older adults with dependency on up to three self-care activities for daily living such as food, mobility, hygiene, but do not have cognitive impairment or have controlled alteration; and degree III are older adults who require assistance in all self-care activities, in addition to the presence or absence of cognitive impairment.

The informal categorization of older adults leads to the determination of their needs, demands and conditions as well as limits their ability to express themselves and be autonomous, since they are inserted in a controlled and regulated environment. LTCF is an institutionalization model that is loaded with control, discipline and division. This control aims to ensure daily work dynamics continuity^(20)^.

The discourse of a unit manager points out that the care provided in an LTCF tends to make older adults more dependent on what the course of the years imposes on them. Evidence from the literature indicates that care centered on older adults tends to improve life experience, health outcomes and quality of care, as it starts with assistance based on the needs and preferences of those being cared for^(21)^.

The manager’s speech also points to the issue of older adults’ independence as a process not yet carried out in practice by professionals, despite raising reflections among them. In this regard, it is relevant to consider that disability is not characteristic of an individual, but something that he becomes. Furthermore, the central issue is how people become and are made incapable and, in view of this, what are the possibilities for articulating alternatives^(19)^.

There is also dissatisfaction with the situation experienced, while at the same time conforming to the scenario of scarcity of human, financial and structural resources. The discourse reinforces older adults’ dependency as a complex issue to be handled by LTCF and that the need for help from dependent older adults makes them more dependent, because possibilities were not thought and created to allow them to carry out their self-care activities without the help of a caregiver. It is important to consider that the environment plays an important role in limiting or favoring older adults’ independence in their daily tasks, as it has a direct impact on quality of life and demand for care^(21)^.

Care for older adults involves considering them as a being in their entirety, understanding aging as a complex process permeated by diseases, disabilities, dependency and loss of autonomy. From the perspective of care, it is understood that “good care” and “bad care” may be obvious at times, while not at others, as different environments carry different complexities and ambivalences. Sometimes good intentions can have bad effects; good practices may contain some intrinsic evil; good care can become a goal for even better care; and sometimes it is simply not clear what kind of care is provided^(22)^.

It is perceived that there is still a long way to go for person-centered care, which refers to a care approach that goes beyond the disease and social context, focusing on individuals and their uniqueness to become a reality in LTCF. It is emphasized that this is an approach that values dignity, respect, compassion, without neglecting the emotional, social and practical needs of the person being cared for and their caregivers. It constitutes an alternative for LTCF to escape the total institution model, based on practices that involve attentive and respectful listening and appreciation of older adults^(21)^.

Care can be perceived in moments that go beyond meeting basic health needs, it can be demonstrated by holding an arm at the right time or going to have a hot chocolate for a conversation. In practice, what reflects good care can reflect not only different values in relation to it, but also involves different ways of ordering reality. Thus, care implies negotiation about the different “goods” that can coexist in a specific and given local practice. It is important to emphasize that care practices must be seriously attended to so that they do not erode. Otherwise, they will be subject to regulations and rules that alienate them^(22)^.

It is common for LTCF s to have their practices structured around a disciplinary power, being marked by strict rules and daily routines, with well-defined schedules^(23)^. In view of this, care is sometimes marked by discontent and rigidity. Rigidity in norms and dissatisfaction with the lived experience. Forms of coercion and sanction are so frequent that they become something naturalized. Forcing an older adult to take a shower seems to be part of the routine, and respect for the other’s body ceases to exist in the face of the need for cleanliness and hygiene. Such behavior presents a setback in any type of argumentation and the failure of a structured disciplinary society.

According to Foucault^(14)^, use of physical force as punishment of the body had been extinguished since the 19^th^ century, since, from then on, the punishment was of the soul, reaching the heart, intellect, will and dispositions. In this way, relations are permeated by power, which starts from the processes of subjection, permeating the uninterrupted and continuous processes that subject bodies and govern their behavior; power of some over others, constituted progressively in the multiplicity of bodies, forces, matters and desires^(24)^.

From this perspective, it is necessary to look at care in a different way so that it is based on practices that allow transforming caregivers of older adults into a LTCF with a potential instrument that frees them from a practice based on norms and encourages, in fact, independence. This practice would avoid the subjection of older adults in relation to those who care for them, since the determination of the other’s disability is a process that can be traced genealogically and that emerges from a game of power and knowledge, through mechanisms of regulation^(19)^. In this way, the power game between caregiver and care takes place as a way of keeping power centered on the institution, on the norms, on the speeches, on the behaviors, on the routines and on the imposed rules, which makes older adults maintain their condition of dependency due to their fragile and senile identity.

### Study limitations

This study has as a limitation the fact that it was carried out in only one institution and due to female homogeneity in the study participants.

### Contributions to nursing

The study made it possible to know the care practices instituted in a LTCF, which are based on the collective and common sense of those who provide care, without taking into account older adults’ specificities and scientific evidence. In the institution, a protocol for classifying older adults’ functionality is not defined, which means that all dependent older adults are treated in the same way, regardless of the reason why they depend on help. The lack of encouragement for independence means that it needs more and more care, resulting in a growing overload of professionals. At the same time, this care is transformed into a loss of autonomy for the hospitalized older adults, as, in a way, LT C F management imposes the institutionalization of a standard of care where products, who are older adults, are cared for in the time established by the professionals and institution, and not in the time of their needs and demands.

Although there is a regulatory requirement for the presence of nurses in LTCFs, this professional’s work process is not yet well defined, with accumulation and overlapping of functions, overload, focus on solving emerging demands, often leading to a work that is not very reflective. This situation hinders the nurses’ work process, resulting in a generalist care that complies with institutional demands and rules, however, lacking in person-centered care. It is not sensitized to meet the desire and will of those who receive it, as there is no clarity to care for; goes beyond a shower or a meal. It is not just to remedy the physical demands, but also to listen to those who are cared for and to do everything possible so that care is individualized and humane. Thus, this study can contribute to a reflection on the work processes of the multidisciplinary team that provides care to older adults in LTCF s, with emphasis on the nursing team.

## FINAL CONSIDERATIONS

From the perspective of the professionals in this study, caring for older adults with disabilities constitutes the challenge between caring and maintaining independence. The care provided by professionals can be seen as a way to protect older adults from any intercurrence that may happen to their physical integrity. In relation to professionals, it is performed as a way of safeguarding themselves regarding issues related to negligence, malpractice and imprudence of their acts. However, this form of care does not encourage older adults’ independence, and one of the main factors that negatively influence this process is cognitive impairment and using self-help devices by older adults.

It is important to point out that working on a LTCF is not a simple task. Caring encompasses particularities and specificities, especially when the person being cared for is an older adult. Associated with this, there is no incentive for professionals to train and motivate themselves for the work they perform, becoming a set of automatic tasks that homogenize not only their practices, but the lives of those who are cared for. This homogenization materializes in classifications and categorizations of older adults as a way of standardizing conduct and behavior and marking deviations. Thus, the care provided does not always respect individuals’ needs, and there is no flexibility of norms and routines, which end up prevailing in the institution.

Thus, older adult-centered care must be taken as a premise so that their individualities are respected and their demands are met. Moreover, guiding care with older adults makes it possible to encourage their independence, allowing them to transpose the role of dependent and subject subjects to individuals capable of deciding for themselves. Therefore, there is a gap in guaranteeing means for older adults to be cared for according to their needs, understanding that this achievement directly impacts older adults’ care and quality of life.

## Supplementary Material

0034-7167-reben-76-s2-e20220767-suppl01

0034-7167-reben-76-s2-e20220767-suppl02

0034-7167-reben-76-s2-e20220767-suppl03

0034-7167-reben-76-s2-e20220767-suppl04

0034-7167-reben-76-s2-e20220767-suppl05

0034-7167-reben-76-s2-e20220767-suppl06

0034-7167-reben-76-s2-e20220767-suppl07

0034-7167-reben-76-s2-e20220767-suppl08

0034-7167-reben-76-s2-e20220767-suppl09

0034-7167-reben-76-s2-e20220767-suppl10

0034-7167-reben-76-s2-e20220767-suppl11

0034-7167-reben-76-s2-e20220767-suppl12

0034-7167-reben-76-s2-e20220767-suppl13

0034-7167-reben-76-s2-e20220767-suppl14

0034-7167-reben-76-s2-e20220767-suppl15

0034-7167-reben-76-s2-e20220767-suppl16

0034-7167-reben-76-s2-e20220767-suppl17

0034-7167-reben-76-s2-e20220767-suppl18

0034-7167-reben-76-s2-e20220767-suppl19

0034-7167-reben-76-s2-e20220767-suppl20

0034-7167-reben-76-s2-e20220767-suppl21

## Data Availability

https://doi.org/10.48331/scielodata.SZTYSJ
