# Supplementary material for: Care for older adults with disabilities in Long Term Care Facility
Source: Rev Bras Enferm. 2023 Dec 8;76(Suppl 2):e20220767. doi: 10.1590/0034-7167-2022-0767 (PMC10704689; doi:10.1590/0034-7167-2022-0767)
Supplement: 0034-7167-reben-76-s2-e20220767-suppl01 [file 0034-7167-reben-76-s2-e20220767-suppl01.pdf]

EI 4

1) Pesquisador 2: **De quem foi a decisão de você vir morar aqui? Por quê?**

EI 4: Qual a decisão?

*Pesquisador 2: De quem que foi?

EI 4: Olha, foram perdas, que eu tive na vida. Perdi em épocas diferentes, toda a minha família, perdi pai, perdi os irmãos e por fim perdi minha mãe. E fiquei sozinha, sem mais ninguém, nesse clã, né?! Familiar. E depois, o que eu tinha de patrimônio, eu perdi também, aí eu fiquei zerada. Então, comecei a ficar mais dependente, mais doente, mais coisa e tal, resolveram os amigos, um grupo resolveu: "ah vão fazer isso e tal e tal", acharam melhor, com certeza também não queriam ficar, tomar aquele problema pra eles, olhar a Alda e tal. Então acharam bom, eu vim pra cá, aí tô aqui. Não foi mesmo uma opção assim, foi uma necessidade, mais necessidade, do que mesmo opção.

2) Pesquisador 2: **Como é o seu dia a dia aqui? Como é, para você, morar aqui?**

EI 4: Você quer assim que eu relate o que acontece?

*Pesquisador 2: Sua rotina.

EI 4: O que acontece ou como é para a Alda, assim?

*Pesquisador 2: Como é o seu dia-a-dia, a sua rotina aqui.

EI 4: Assim, levantar, tomar café, essas coisas, assim? Que cê que? Então bom, a minha vida é assim, uma vida quase que reclusa, rs, dentro do meu quarto, porque eu levanto, faço as refeições junto com o pessoal da casa, e eu tenho liberdade de ir e vir em qualquer lugar sem acompanhante, desde que eu tenha possibilidade física e saudável, de saúde e posso viajar, não tem nenhuma restrição quanto a isso. Então quando eu tô bem, eu saio por aí mesmo, vou passear, então não tem, outro dia eu fui sozinha ver o show do Chico Buarque, aaaah... mais então essa é minha vida e como eu gosto muito de ler, como eu não tô dando mais conta, né? Então a leitura tá meio afastada, meio não, totalmente afastada e tô, então eu arranjei já, antes de vir pra cá, quando eu me aposentei, é um artesanato, então eu faço um tipo de, até tem ali na diretoria, depois cês olham ali eu faço aquele tipo de estandarte, sabe? Faço desse, desse e desse tamanho, então isso foi a coisa

que eu criei, que ninguém me ensinou, eu pesquisei e fiz, e a noite eu gosto muito assim de documentário, mas aqui eu não tenho multicanal, só pega essas estaçõezinhas, então não sou noveleira, gosto mais de documentário, como eu falei, e vejo os jornais, mas assim eu vejo um, passo pra outra estação, as vezes as mesmas palavras que eu ouvi no primeiro vou ouvir no segundo e pronto.

*Pesquisador 2: E como é pra senhora morar aqui?

EI 4: Se é bom ou ruim?

*Pesquisador 2: É.

EI 4: Olha, num pode ser, é muito difícil, cês imaginam uma pessoa que tinha um padrão de vida, morava ali na Francisco Sales, com Maranhão, num apartamento de três, quatro quartos e vim morar numa casa dessa, com pessoas assim, ah é muito difícil, num é fácil, de um apartamento, ir morar num quarto. Então eu não tenho outra opção, eu não tenho.

*Pesquisador 1: A senhora sempre morou sozinha?

EI 4: Não, sempre morei com minha mãe, sempre, eu sou a caçula e a que fiquei solteira, não quis casar mesmo, namorei deeeemaaaais e minha mãe viveu até 102 anos, lucida inteiramente, graças a Deus, e quando eu perdi minha mãe, eu perdi o chão, sabe?! E aí eu fui perdendo tudo, vendi o apartamento, cê vende, não faz nada, vai pegando o dinheiro e o que, que faz? Nada. A única coisa que eu fiz boa nesse período que eu vendi o apartamento foi a Itália.

*Pesquisador 1: Foi o que?

EI 4: Ir a Itália, que a Itália realmente vale a pena ser vista, e foi, meu objetivo maior num era mesmo a Itália em si, mas era Assis, minha paixão por São Francisco é antiga e grande. E eu fui sozinha, fiquei quatro dias, isso deve ter o que uns quatro anos só, quatro cinco anos no máximo, sabe? Tranquilamente e foi realmente uma viagem linda, Itália deve ser vista mesmo, se cês não foram procurem ir, cultura tá ali, beleza tá ali.

3) Pesquisador 1: **Me fale um pouco sobre seu relacionamento com as pessoas que trabalham aqui.**

EI 4: Aqui? Você fala na gerência?

*Pesquisador 1: De forma geral, os cuidadores.

EI 4: Oh eu não preciso de cuidadora, né?! É, mas assim na hora da medicação, sim, elas

é que levam pra gente, fora isso eu faço tudo sozinha, né?! Não tem muita dependência, tem umas assim mais, tem até uma cuidadora aqui que ela não dá, ela, rs, eu falo assim porque que essa moça não gosta de mim, ela não me cumprimenta nem a pau, ela passa assim. Então tem umas, tem dificuldade, a eu acho assim, elas deviam ser orientadas mais, falta, eu acho, eu sempre falei, falta alegria e ternura aqui nessa casa, sabe?! Isso eu falo mesmo, já falei ate com a gerencia, porque na outra casa era muito mais alegre, sabe?! E isso me faz falta viu, eu sou de gargalhada, sabe?! Eu dou muito hahaha e agora com a gerencia, brigo, faço as pazes e tal, reclamo, falo e questiono, sabe?! E mesmo com a parte de saúde, eu questiono muito esse medicamento, pra que que é? Pra que que, não é? E também, eu acho assim que falta um pouco de higienização das cuidadoras, por exemplo tem que pingar um colírio no meu olho, já pingou naquela de lá e não higieniza as mãos, nem nada, eu vejo aqui muita dificuldade, eu não vejo elas quase enluvadas, na outra casa não, falei tanto, elas gastavam muitas luvas. Então eu acho nessa área, vocês não vão mostrar a casa não né?! A minha entrevista vocês vão passar pra casa?! Rs.

*Pesquisador 1: Não.

*Pesquisador 2: Não.

EI 4: Mas eu já falei isso, eu sou muito franca e muita verdade, detesto mentira, então eu acho que essa parte assim é bem falha, sabe?! Essa pra mim é falha, e eu sou muito observadora, olho muito, sabe?! Às vezes eu até vejo as coisas sem querer, então nessa parte eu acho que deixa a desejar bastante.

*Pesquisador 1: Entendi.

4) Pesquisador 1: **Agora, me fale sobre seu relacionamento com os outros idosos que moram aqui.**

EI 4: Ah muito boa, converso brinco assim, igual as vezes me contam alguma coisa sabe?! “Ei tudo bem?” “Ei gente!” Igual quando eu saio da mesa: “com licença, já acabei” então é legal, mas não é assim aquele relacionamento que eu teria com vocês, um bate papo, não né?! Diferente né?! Mas elas um relacionamento carinhoso, é um relacionamento humano, num é assim de desprezo, absolutamente.

*Pesquisador 1: A senhora tem amigas aqui?

EI 4: Não, não.

5) Pesquisador 1:**Você mantém contato com outras pessoas de fora da**

Instituição. Se sim, com quem e que tipo de contato é esse?

EI 4: Só tem.

*Pesquisador 1: É? Conta pra gente, com quem...

EI 4: Não é assim, só tem não, meus relacionamentos são com pessoas que já eram minhas amigas, num aqui dentro num tenho né?! Eu tenho uma prima, tenho uma afilhada, meus colegas, tenho meus colegas de... de... de... grupo, de curso primário, tenho colegas de faculdade que ate hoje vem, a gente sai faz um lanche, alguma coisa, toma uma cervejazinha, sabe?! Que eu gosto muito, rs. Então é isso, esse relacionamento, é um relacionamento social de... de também, um relacionamento de confidencias, né?! De trocas, relacionamento de pessoas amigas, é isso.

6) Pesquisador 1:**Você se sente em condições de tomar decisões sobre as coisas que precisa fazer no dia-a-dia? Por quê?**

EI 4: Eu não entendi o início.

*Pesquisador 1: Se a senhora, se sente em condições de tomar decisões.

EI 4: Ah se eu sinto em condições, de... de... ah, disso eu sinto, eu faço o que eu quero.

Pesquisador 2: Mesmo aqui dentro da casa?

EI 4: É nesse sentido?

*Pesquisador 1: É.

*Pesquisador 2: Mesmo aqui dentro da casa a senhora se sente em condições de tomar suas decisões?

EI 4: É por exemplo tem um grupo que faz um trabalho aí, com uma das psicólogas, eu não faço, porque? Porque não quero, então elas já falaram você faz o que você quiser, você é dona de você, e eu sou mesmo, então só faço o que eu quero, ninguém me força nada, disso nem precisa de pensar, sou muito independente.

*Pesquisador 1: Então a senhora considera que faz aquilo que a senhora gosta, que a senhora quer.

EI 4: É o que eu quero, rs. Rebelde....
