# Supplementary material for: Care for older adults with disabilities in Long Term Care Facility
Source: Rev Bras Enferm. 2023 Dec 8;76(Suppl 2):e20220767. doi: 10.1590/0034-7167-2022-0767 (PMC10704689; doi:10.1590/0034-7167-2022-0767)
Supplement: 0034-7167-reben-76-s2-e20220767-suppl02 [file 0034-7167-reben-76-s2-e20220767-suppl02.pdf]

EI 6

1) Pesquisador 2: **De quem foi a decisão de você vir morar aqui? Por quê?**

EI 6: Eu fazia parte da associação Santa Zita, associação, aqui era casa das domesticas, então aí entrei em 1982, né?! De onde eu morei no colégio ali em cima, eles me, a irmã me trouxe aqui pra poder quando eu aposentasse, eu podia, quando tirasse férias, fosse mandada embora do emprego né?! Então aí tá, eu aposentei e não vim não, né?! Continuei minha vidinha lá fora, mas como aqui tava mudando, e a prefeitura agora tá junto né?! Então tinha três vagas aqui, que tinha um tempo pra preencher essas vagas, aí eles me convidaram pra eu vim pra cá, aí eu vim, nunca pedi não, gostei, fui convidada a vir. Mas eu já era aposentada, tava morando sozinha.

*Pesquisador 1: Aí antes disso, a senhora morava na outra casa?

EI 6: Depois que eu aposentei, eu tava morando lá no bairro Jaqueline na minha casa, mas é alugada, né?! Mas era minha casinha, muito bonitinha. Rs.

*Pesquisador 1: Aí a senhora mudou de lá pra cá?

EI 6: É, aí eu vim pra cá, aí eu fui convidada e vim pra cá, sou muito feliz aqui.

2) Pesquisador 2: **Como é o seu dia a dia aqui? Como é, para você, morar aqui?**

EI 6: De manhã, levanto cedo, levanto 5:30, por aí, apronto, vou pra missa, que eu fiquei responsável pela missa das 7hs, na Boa Viagem, né?! Que eu canto lá, e aqui quando precisa de mim, eu tô sempre as ordens, né?! Que as vezes eu saio com pessoas pra levar ao banco, as vezes a irmã pede pra ir no posto né?! Buscar pedido ou levar, ou qualquer coisa assim que precisar, acompanhar as pessoas no médico assim e a tarde sempre a gente tem a oração da tarde, 15hs. A noite as vezes eu tenho programa na igreja, hoje mesmo eu tenho ensaio, eu participo do coral lá, né?! E assim, mexo com a horta, com o jardim.

*Pesquisador 2: E como é pra senhora morar aqui?

EI 6: Pra mim?

*Pesquisador 2: É.

EI 6: Eu acho ótimo, sabe porque, se eu tivesse assim dependente eu acho que não ia ser tão boa assim, rs. Mas como graças a Deus né, eu ainda tenho condições de ajudar, de

poder servir, então pra mim tá sendo ótimo, porque eu tô tendo a oportunidade de ajudar minhas companheiras, né?! Ajudar na casa, ser útil.

*Pesquisador 1: E porque que a senhora acha que se fosse dependente, não seria tão bom morar aqui?

EI 6: Ah, porque a gente vê assim, quer dizer, eu como tô me preparando pra esse momento é uma coisa, agora quem vem assim, que o negócio assim o cê vim, outra coisa é você ser trazida, sabe?! Então tem pessoas que do jeito que fala que vieram, as vezes foi assim meio violento assim né? As vezes mente pras pessoas, né?! Então fica um pouco sofrido, que chega aqui, elas custam a adaptar, né?! Fica querendo ir embora, fica querendo fugir, rs... Entendeu? Então, eu acho que assim que hoje tá tendo muito esse preparo, porque antigamente a pessoa não vivia tanto assim, né?! Eu não achava que fosse chegar aos 50 anos, cheguei aos 70 com essa força toda aí. Então, as pessoas já chegam assim muito assim né, é triste, sabe?! Porque fica aqui os parentes não vem visitar tanto assim, como deveria vir né?! Isso é bom pra vocês aí, terem consciência que seus paizinhos, sua vozinha, então que põe aqui, aqui é ótimo a pessoa é muito bem tratada, com muito carinho, com tudo, mas não é igual em casa, eles sentem falta dos parentes, por isso eu procuro assim, preencher o vazio delas, eu brinco com elas, canto com elas, brinco, bagunço, aquelas que tão muito triste, eu sabe?! Mexo, mexo até que elas eu consigo fazer elas dar uma risada, abrir, porque tem umas que fica, sente que elas tão assim vagando, eu fico assim, meu Deus o que ela tá pensando, né?! Então fica assim, eu pra mim não, mas eu sinto que tem muito solidão, entendeu?! Então é nesse ponto que eu digo sabe?! Que eu penso assim, eu no meu modo de pensar, eu acho que viver é uma coisa, agora vegetar eu acho outra coisa, eu acho que chega o momento que a pessoa nem tá vivendo mais, né?! Então levar pra cá ela vai, cê da comida ela come, se cê num dê ela não come, então fica uma vida assim, eu acho muito assim, sabe?! É triste, não é fácil não, então isso aí, por aí entra o bom senso de quem cuida, né?! Pra preencher esse vazio das pessoas, a gente percebe, né?! Então eu da forma que eu faço a minha parte né, já falei com Jesus, olha enquanto eu tiver podendo ajudar, enquanto eu tiver vivendo, podendo ser útil, num é que eu não queira ser, entendeu?! Então tudo bem igual se eu vê que, como diz o outro "não presta pra nada mais" rs.. Aí senhor pode me levar, que eu tô prontinha, sabe? Que tem gente que agarra, né?! Não tá aguentando nem tá ali insistindo, as vezes num pode, "não vai ali, se for ali você cai", aí tema e vai e cai e depois machuca e tem que ir pro hospital, entendeu?! Então tem que saber envelhecer, cê sabendo

envelhecer, beleza, teve aqui a Rosa, ela morreu com 104 anos, um exemplo de vida a Rosa, sabe?! Alegre, brincalhona, feliz da vida, tem aí a dona Geralda 103 anos, né?! Cuidando do papagaiozinho dela, toma conta de mim né?! Que eu mexo no jardim, aí eu tomo conta o jardim e ela toma conta de mim, rs. E tem a Cristina, né?! Que é aquela pretinha que fica lá estudando, cê vê que gracinha, preenche a vida dela, tá lá estudando bonitinho, quer dizer tem o que fazer, entendeu aí é uma coisa, agora pra quem não se prepara, aí fica assim uma coisa muito vazia, sabe?! Então nesse ponto que eu tiro, sabe assim, que a gente sente que a pessoa fica triste, né?! Então não é fácil não, num é fácil não, mas graças a Deus, aqui é muito bem cuidada, tem muito apoio, tem muita distração, aqui evento, nossa senhora, a semana inteira. As pessoas vêm aqui cantar pra gente, brincar, jogar, é... bingo né?! Vem dançar, cantar, tocar, essas festas, todo domingo, todo final de mês tem aniversario, eles programa passeio pra gente, sabe?! É a primeira dama veio aqui nos visitar, no final do ano, nos convidou minha filha, pra ir participar de show, aí no Palácio das Artes, eu fui dois, agora chega, rs. Então nós fomos assistir grupo de dança, fomos assistir um moço aí que veio cantar, fazer um evento baseado no foi no Chico? Acho que no Chico Buarque, MENINA, lá de camarote lá oh, tudo madame lá, rs.. Então é assim quando você tá vivendo, assim tá com, como se diz, consciente né?! Assim podendo mover, pelo menos cê poder assim, olhar e participar, vale a pena, a gente leva as meninas, levam de cadeira, a gente vai no parque municipal, fazer atividade, lá, sabe?! Então é muito bom, só que eu no meu modo de pensar, eu no meu modo né?! Pra mim eu não quero viver tanto assim não, rs.

*Pesquisador 1: A senhora não quer ficar é dependente né?

EI 6: Num é dependente, também, não é só dependente que eu acho meio bobo assim, sabe?! Cê fica sem fazer nada. Eu quebrei meu braço, né? Tava indo pra missa, um belo tropeção na hora que eu atravessei a rua, sai assim fala catando cavaco, sai tchinn e não parava não minha filha, Jesus misericórdia, aí eu cai, a gente faz assim... minha mão bateu no meio fio, quebrou isso aqui, aí eu me virei assim mesmo, fazer o que né?! Fui pra missa assim mesmo, cantei a missa, voltei minha mão tava dessa altura, “irmã quebrei o braço”, aí não quis ir pra Upa não, fui no pronto socorro, eles engessaram né?! Aí eu fui e me virei assim mesmo, aí pude experimentar, assim que depende muito de você também ter uma força de vontade, que as vezes a pessoa, né?! Se entrega, então eu tô trabalhando muito isso, muito isso em mim, pra assim quando eu penso, né?! Porque o idoso pode

fazer, deve deixar ele fazer, né?! Porque se não a pessoa se entrega ali e oh, fica muito monótono né, eu acho.

3) Pesquisador 2: **Me fale um pouco sobre seu relacionamento com as pessoas que trabalham aqui.**

EI 6: Muito bom, muito bom, graças a Deus, com umas mais, né?! Sempre tem as que você tem mais afinidade né, outras menos que é normal, né?! Mas o relacionamento em si, graças a Deus muito bom, não tenho que queixar de nenhuma delas, são muito assim, cuida bem das, das, eu falo as meninas né, tem paciência com elas, as vezes assim, que como eu tô boa, eu ando pra ir, as vezes tem uma lá precisando de socorro, né, eu peço passo pra elas, e as vezes tem umas que sai vai tomar sol e chega lá, ela esquece da vida né?! E como, elas tão mexendo com outras coisas pra cá né, eu vou lá dou uma, "oh fulana tá lá no sol, posso tirar?" "Pode." E a gente brinca muito, sabe, quando tem missa aqui, eu convido elas pra fazer a leitura, né?! Na missa, é muito bom.

4) Pesquisador 2: **Agora, me fale sobre seu relacionamento com os outros idosos que moram aqui.**

EI 6: Com elas nem se conta, são meus dodói, rs. Nossa, minha paixão eu gosto, muito, muito delas, como eu falei aqui eu arrumei filha, que é aqueles que depende muito, né?! Arrumei mãe, são as que eu saio com ela, a Cristina, eu saio com a Cristina, todo mundo fala é mãe e filha, rs. Tem as que é minha a mãe, a Eugenia, diz que eu sou sobrinha dela, então eu tô em casa, tem minha família. Então como eu falo muito, com a gente, nos aqui temos as que são mais novas, que tá com a cabeça assim como a minha, gente nós temos que ser bem unidas, pra gente viver como irmãs, né?! Uma assim, é querer o bem pra outra, tentar ajudar a outra, né?! E assim, a se alegrar com a outra, pra gente formar, aqui a gente nós formamos uma família, igualzinho em viu, tem briga, tem, rs. Mas a gente é amiga e uma defende a outra, aí de quem, uma vê a outra falar qualquer coisa, essa Eugenia que eu tô falando, minha tia, um dia uma delas tava brincando comigo, a gente brinca né?! Ela ficou escutando assim, chegou "o que que é que ela tá falando com você?" "Nada não, nos tão brincando" rs.... Então é muito gostoso, muito gostoso mesmo.

5) Pesquisador 2:**Você mantém contato com outras pessoas de fora da Instituição. Se sim, com quem e que tipo de contato é esse?**

EI 6: Demais, nossa Senhora.

*Pesquisador 2: Com quem a senhora mantém?

EI 6: Oh tem com as pessoas de onde eu morei, né?! Aquelas pessoas da igreja, pessoas que fica minhas amigas e as vezes nem conheço, né, nem sei, porque como eu sou uma só, são muitos né, outro dia eu tô lá na igreja “oh que não sei que lá”, me cumprimentando e me beijando e eu meu Deus quem é, “que cê sumiu” e eu tô assim da onde, rs. De onde meu Deus, de onde eu conheço essa pessoa. Então, graças a Deus, tem muito contato mesmo e lá é assim, na igreja, como, eu sinto que de repente você conhece todo mundo assim, no olhar, né?! Então assim, eu sinto falta de alguém, eu pergunto porque não tá vindo, sabe, fico preocupada se tá doente, e a mesma coisa se eu falto de missa, quando eu quebrei o braço, eu fiquei uma semana sem ir, ficou todo mundo lá, perguntando por mim, sabe?! Vieram aqui me ver.

*Pesquisador 1: As pessoas vêm aqui visitar a senhora?

EI 6: Olha, eu, visita minha mesmo eu tenho pouca visita, assim, mas as visita dos outros são minhas visita, rs.

*Pesquisador 1: E outras pessoas recebem muitas visitas?

EI 6: Não, algumas sim, outras não, é, tem uma senhora aqui que ela recebe visita todo, umas quatro, cinco vezes na semana, do filho dela, sabe?! Uma moradora. E tem outra também que vem os netos, vem quatro gerações, eu acho lindo, sabe?! Também vem muito visitado, agora outros recebem mais ou menos, mais recebe, tem um ou outro assim, que a gente não vê quase ninguém visitar, mas porque os parentes não moram aqui, né?! Mais os que moram aqui recebem visita, e fica assim, a gente é tão unida, que visita de uma é visita da outra, por exemplo chega uma visita pra uma, se não me vê, já pergunta: “cadê a Graça?” né?! Na hora que eu chego, fazendo aquela festa pra mim, como se a visita fosse pra mim também. Então assim, o que é de uma é de todos, uma casa não chega uma visita, na sua casa, vai visitar só sua mãe? Ou só você, né a visita é pra família né?! Então eu sinto assim, sinto em casa aqui, me sinto em casa.

*Pesquisador 1: Que bom.

6) Pesquisador 2: **Você se sente em condições de tomar decisões sobre as coisas que precisa fazer no dia-a-dia? Por quê?**

EI 6: Ah eu tomo, rs. Sim.

*Pesquisador 2: Por quê a senhora acha?

EI 6: Porque eu tô boa ainda, eu vim pra aqui eu tava trabalhando de cuidadora.

Pesquisador 2: A senhora cuidava de idoso?

EI 6: O povo achava a maior graça quando eu falava cuidador de idoso, tudo bem, quando eu falava minha idade, “o quê?” Rs. É, então aqui eu me sinto, graças a Deus.

*Pesquisador 1: Você pode decidir tudo que você faz, a hora que você quer fazer?

EI 6: Eu tenho, eu tô assim eu falo pra elas, por exemplo, vamos supor que tem uma viagem, tem um passeio, eu quero ir, eu não gosto muito de sair não, mas se eu quero, eu decido que eu quero ir, “irmã eu to querendo ir em tal lugar assim, assim, eu posso?” “Pode.” E vou, entendeu, é igual aqui, precisa de fazer alguma compra na cidade, “você pode comprar assim”, “vou”. Mas tudo que precisa aqui de compras, assim tipo comprar peça pra fogão que estragou, peça pra maquina, num sei que lá, é tampa, levar panela pra colocar tampa, é tudo sou eu, sabe, que faço. E por exemplo, igreja, quando tem missa, eu já sei que vai ter missa, eu sei que é muito pras meninas, pras que faz faxina né, que é uma só, eu vou à missa, chego tomo meu café, já desço e vou arrumar a capela, varro, se precisar passar pano, eu passo pano, sabe?! Tiro pó, arrumo tudo direitinho, aí depois a irmã vai só pra fazer a parte dela, né. Então aqui, vida normal, graças a Deus, e elas também contam comigo né, muitas coisas que elas vão fazer, igual aqui, eu levava a menina estava sem receber, a Cristina, ela tava com a conta muito longe, então quando precisa, por exemplo fazer o... como que fala... prova de vida né, lá naquela lonjura, irmã eu posso transferir, trazer ela pra cá? Agora não, agora tem a menina que é a chefe, né?! Vanessa, mais antes era a irmã, pode, eu tomei toda providencia e trouxe a Cristina eu trouxe a transferência pra cá, agora a gente vai aqui, inclusive elas trocam ideia, Graça eu tô querendo fazer isso, o que você acha? Aí eu acho oh, vão devagar, sei lá se vai dar certo. Então assim a gente tem liberdade sabe, graças a Deus, sinto em casa mesmo aqui.

Pesquisador 1: Que bom, isso é muito importante.
