# Supplementary material for: Care for older adults with disabilities in Long Term Care Facility
Source: Rev Bras Enferm. 2023 Dec 8;76(Suppl 2):e20220767. doi: 10.1590/0034-7167-2022-0767 (PMC10704689; doi:10.1590/0034-7167-2022-0767)
Supplement: 0034-7167-reben-76-s2-e20220767-suppl03 [file 0034-7167-reben-76-s2-e20220767-suppl03.pdf]

EI 8

1) Pesquisador 1: **De quem foi a decisão de você vir morar aqui?**

EI 8: É... decisão minha mesmo. Uma opção que eu fiz.

*Pesquisador 1: Por quê?

EI 8: Porque meus pais já tinham falecido né?! E... meus irmãos casaram. Só eu solteira, eu já estava trabalhando aqui em Belo Horizonte. Aí quando eu me aposentei, aí eu fui convidada pra morar aqui. A irmã que coordenava a casa me convidou. Aí eu fui, falei com meus familiares, eles não gostam muito não, mas falei assim ah... eles já tem os problemas, família deles, tudo né?! Aí eu... acho que eu posso dar conta... sozinha (rs).

*Pesquisador 1: E o que?

EI 8: Eles aceitaram.

2) Pesquisador 1: **Como é o seu dia a dia aqui?**

EI 8: O meu dia a dia aqui é muito bom porque (rs) eu vou à missa de manhã, depois eu volto, vou fazer minhas coisas, meu quarto. Eu que limpo, eu que lavo minhas roupas, eu que passo, e... Tem as atividades da casa também. Teve até umas meninas que falam aí: ah... a nossa vida aqui é muito boa, é comer e dormir... mas não é tanto assim também não né?! (kkkk) Tem tem uns afazerezinhos né?! Quando eu tô assim mais coisa, tem essa palavra que eu gosto né?! E... pra ajudar a memória também né?! Eu gosto de ler. Então, a gente vai levando os dia aí né?! Bate papo com uma com outra, 3 horas da tarde vamo reza o terço na capela. E...

2) Pesquisador 1: **Como é, para você, morar aqui?**

EI 8: Uai... tranquilo... eu gosto de morar aqui. Já acostumei, a gente vai aprendendo também, até a gente... com as que tá mais assim... debilitada, a gente vai aprendendo também né?! Quando, a gente não sabe que amanhã é o futuro da gente né?! Então é um aprendiz não é?!

3) Pesquisador 1: **Me fale um pouco sobre seu relacionamento com as pessoas que trabalham aqui.**

EI 8: Ah... o meu relacionamento com elas é sempre bom, porque eu não tenho do que reclamar delas, não sei se elas têm o que reclamar de mim né?! Porque eu procuro tratar todas iguais né?! A gente tá sempre assim, na boa né?! Por mim, tá tudo tranquilo.

4) Pesquisador 1: **Agora, me fale sobre seu relacionamento com os outros idosos que moram aqui.**

EI 8: Também! Às vezes tem umas coisinhas assim coisa, a gente não leva a sério não porque (rs), a gente respeita a idade né?! Cada um tem seus problema, então a gente não pode levar a sério né?! Então, a gente vive.

5) Pesquisador 1: **Você mantém contato com outras pessoas de fora da Instituição. Se sim, com quem e que tipo de contato é esse?**

EI 8: Sim, tem umas colegas lá fora. Eu tenho um grupo também que a gente reúne as... quarta e domingo, elas vêm aqui. A gente conversa e tal, até faz uma reunião aqui.

*Pesquisador 1: Então você recebe as pessoas aqui também?

EI 8: Recebo! E tenho, eu sou lá da Igreja também né?! Tenho colega que eu vou na casa delas, elas vêm aqui visita também.

*Pesquisador 1: A sua família, cê mantem contato?

EI 8: Sim, sim. Até semana passada mesmo teve aqui a minha sobrinha e o filho dela. Quando eu viajo, eu fico na casa dela. Aí ela veio, que o menino dela veio fazer uma prova no domingo. E... eles ficaram aqui (inaudível). Foram embora na terça feira, chegaram sábado a tarde e foram na terça.

*Pesquisador 1: E cê acha que a Instituição de alguma forma, ela incentiva esse contato de vocês?

EI 8: Sim, sim, incentiva sim.

6) Pesquisador 1: **Você se sente em condições de tomar decisões sobre as coisas que precisa fazer no dia-a-dia? Por quê?**

EI 8: Sim... é claro que a gente as vezes tem coisas que a gente tem que, é... tem que falar né?! Com a coordenadora, o que cê vai fazer e tal, né?! Mas por mim a decisão, eu sei tomar. Mas é... como eu moro aqui, então a gente tem que é... obedecer né?! Dar satisfação.

*Pesquisador 1: Você acha que todas vocês tem condições de tomar decisão sobre as coisas do dia a dia?

EI 8: Todas não.

*Pesquisador 1: Por que?

EI 8: Umas 5, mais ou menos.

*Pesquisador 1: Por que você acha que não são todas?

EI 8: Oh... Ah... porque a gente vê né?! Que elas tem mais dificuldade né?!

*Pesquisador 1: Mas em que sentido?

EI 8: Uai, até no conversar mesmo né?! Elas vão falando uma coisa e nã...nã... (não consegue se expressar), como é que fala?! Nã... não completa aquela frase. Então, eu acho que elas não têm condição de tomar decisão sozinhas. Elas precisa de alguém pra ajudar, pra dar banho, pra arrumar. É nesse sentido que eu... que eu penso.

*Pesquisador 1: Você acha que coisas mais simples assim, como tomar banho, comer, essas coisas, elas conseguem decidir?

EI 8: Algumas sim, outras não.

*Pesquisador 1: Entendi.

EI 8: A maioria não né?!

*Pesquisador 1: E porque que que cê acha que a maioria delas não consegue?

EI 8: Ah... eh... porque eh... tem dificuldade mesmo. Tem umas que já teve AVC né?! E... que não movimenta os braço, as vezes a perna também não tem. Então, eu acho assim né?! Ou tá na cadeira de roda. Tem umas que num... tem umas que não tá na cadeira de roda, mas precisa de ajuda...

*Pesquisador 1: Por que elas são dependentes?

EI 8: Ah... eu acho que sim.

*Pesquisador 1: Entendi

EI 8: Tem umas às vezes até... que as vezes até dá conta, mas se... se deixar elas não tomam banho, não trocam de roupa. Nesse sentido aí mesmo...
