# Supplementary material for: Care for older adults with disabilities in Long Term Care Facility
Source: Rev Bras Enferm. 2023 Dec 8;76(Suppl 2):e20220767. doi: 10.1590/0034-7167-2022-0767 (PMC10704689; doi:10.1590/0034-7167-2022-0767)
Supplement: 0034-7167-reben-76-s2-e20220767-suppl04 [file 0034-7167-reben-76-s2-e20220767-suppl04.pdf]

EI 15

1) Pesquisador 2: **De quem foi a decisão de você vir morar aqui? Por quê?**

EI 15: Ah, olha foi depende que a gente vai chega numa idade, a gente tem que procurar o lugar adequado, não que a família queira desfazer da gente, mais é por causa do momento né?! Que cê ter um lugar adequado pro idoso, a família precisa trabalhar e não ter condição de manter um cuidador em casa, particular, de maneiras que eu tive opção de vim pra casa Santa Zita.

*Pesquisador 2: Então foi a senhora que tomou a decisão de vir pra cá?

EI 15: Eu tomei a decisão, a família não queria e tudo, mas eu falei: “não chegou a hora”, comecei já com problema de saúde e então eu falei assim, quer dizer o idoso dependendo não são todos, mais a maioria dá trabalho. Então se a gente tem o lugar adequado e pelo menos aqui, aqui por exemplo a casa Santa Zita, é uma casa muito boa, né, que a gente tem de tudo, cuidadora, cuidador, médico, assistente social, né, uma equipe de acompanhamento para nos. Então eu acho, que eu fiz uma boa opção, né eu vir para a casa Santa Zita.

2) Pesquisador 2: **Como é o seu dia a dia aqui? Como é, para você, morar aqui?**

EI 15: Olha o meu dia a dia aqui é vinte e quatro horas na cama, porque eu tive trombose na perna direita e tô fazendo assim até com fisio... em dezembro com a fisioterapeuta da PUC, né, a Débora, é os exercícios pra ver se eu consigo andar, porque nem na cadeira de rodas não tô movimentando né, passo o tempo todo na, o meu dia a dia é na cama, né, mais não tenho queixa.

*Pesquisador 2: E como é pra senhora, morar aqui?

EI 15: Eu acho bom, eu acho bom, e como diz o ditado, né, é o lugar adequado para a minha vida do dia a dia, né, os momentos, pra acompanhamento, né, eu acho ... muito bom.

3) Pesquisador 2: **Me fale um pouco sobre seu relacionamento com as pessoas que trabalham aqui.**

EI 15: Olha, é excelente, tem um, assim um bom comportamento, faço a minha parte, né, e são tem um acompanhamento muito bom, graças a Deus, bom demais, né?!

4) Pesquisador 2: **Agora, me fale sobre seu relacionamento com os outros idosos que moram aqui.**

EI 15: Também relaciono bem, assim respeitando cada uma, o seu jeito, aquela coisa toda, vivo bem com todas, né, quando eu podia ir no refeitório, participava ali de conversa, daquela coisa toda, mais agora eu num vou mais no refeitório nem na cadeira de roda, ainda não tô podendo movimentar muito.

*Pesquisador 2: A senhora sente falta de ir no refeitório?

EI 15: Olha eu sente falta, porque tem as companheiras que a gente bate um papo, pelo menos a gente tá ali vendo todas, a gente rir de vez em quando, tem umas que são assim espirituosas e tudo, então a gente sente falta do ambiente, né, porque ficar sozinha não é bom, mais a gente tem que, como se diz, aceitar que a coisa que eu falo que na vida da gente é aceitação, né, chega um memento que a gente não pode tá querendo, vamos dizer aquilo que a gente quer, né, nem tudo que a gente quer pode, não é mesmo? Então a gente tem que ter aceitação, aguardar o momento certo, né, de recuperação, e que eu possa participar, participei das atividades, sinto falta da capela, de ir, nos rezamos o terço todos os dias, quer dizer eu rezava né, agora não tô participando e então tudo isso faz uma falta, né na vida da gente.

*Pesquisador 2: E a senhora tem amigas aqui na casa?

EI 15: Tenho, tenho amigas, tanto eu tenho amigas com as enfermeiras, as cuidadoras, né, são sempre muito atenciosas comigo, não tenho queixa de nenhuma da casa, tenho mais é que agradecer do que pedir.

5) Pesquisador 2:**Você mantém contato com outras pessoas de fora da Instituição. Se sim, com quem e que tipo de contato é esse?**

EI 15: Parentes e amigos, cê fala assim, visita?

*Pesquisador 2: Isso.

EI 15: Tenho, constantemente, grandes amigas, tenho os parentes, sobrinhos, que vem pelo menos, minha sobrinha já prometeu segunda feira vem aqui, o meu sobrinho também, né.

*Pesquisador 2: E antes da senhora ter esse probleminha na perna a senhora saia, da casa, pra passear, fazer alguma coisa?

EI 15: Não, sempre sai, bom antes de ter o problema nas perna, eu ia ao médico, é quando tinha um passeio eu participava, né, andava pela casa, sempre participei de todas as atividades da casa, né, estava sempre presente.

6) Pesquisador 2: **Você se sente em condições de tomar decisões sobre as coisas que precisa fazer no dia-a-dia? Por quê?**

EI 15: Tenho, eu pelo menos eu falo que as pernas não tá muito boa, mais a cabeça graças a Deus tá, minhas compra, o que que tá faltando, né, tenho a enfermeira da noite que ficou seis meses, é, mais ou menos, ela ficou me acompanhando que eu passei muito mal, né, aí tive que ter uma pessoa só pra me atender, né, ela é enfermeira da noite, né. Então é, aí até eu ter uma melhora, já poder movimentar alguma coisa, eu pego sozinha, né, então, foi bom demais esse momento, mais a gente não pode ficar muito na dependência, que acostuma, né, tem que ter que agir, fazer as coisas, e ter força de vontade, que é o principal, né, não deixar, como se diz assim ah que eu não posso fazer isso, não posso fazer aquilo, ficar só querendo que uma pessoa faça pra ela, não pode. Então eu sempre fui muito independente.

*Pesquisador 2: E a casa proporciona pra senhora essa, a senhora tomar decisão do que quer fazer, o horário que a senhora quer fazer, a casa te dá essa liberdade?

EI 15: Dá, que eles têm assistente social, né, é, e a nossa superiora também, não é mesmo?! Tá sempre presente, perguntando se tá faltando alguma coisa, nos temos um médico geriatra, ontem mesmo ele veio a noite, desde que eu cheguei pra casa, ele me atende, quando eu preciso, ele faz as vista a noite, muito bom, dr. Mauro. Ele é ótimo, né, muito atencioso, tenho que agradecer a Deus por tudo e por todos, né, aqui eu falo assim: Deus nos dá, nos todos temos um anjo, mais eu falo assim: tenho diversos, são muitos anjos ao meu redor, né, muito bom. Tenho o médico da UNIMED, que faz a vista domiciliar, todo mês, a enfermeira, né, talvez ela venha até hoje, porque essa semana começaram a trabalhar, né, feriado já passou e já começaram na atividade. Então essa coisa assim, eu falo assim, a gente tem que saber agradecer que a gente nunca tá sozinho, né, não tem esse negocio de ficar “ah eu tô só”, não, primeiro que a gente tá com Deus, segundo as pessoas que tão ao nosso redor, muito bom, valorizar a vida, o que a gente tem que agradecer a Deus pelo dom da vida, mesmo na ah porque tá na velhice e tudo, a gente não pode falar “eu estou velha”, tem que falar eu estou vivendo e agradecer a Deus pelo dom da vida. Chega um momento que a gente tem que aceitar é, as consequências

da idade, que são diversas, né, então “ah eu fazia isso eu fazia aquilo”, “eu queria”, não, a gente faz o que pode, manter a cabeça no lugar, né, a disciplina é muito importante, obedecer os horários dos medicamento, das refeições, isso tudo é importante na nossa vida, na vida do idoso, né, eu falo não é idoso, é a velhice mesmo, eu falo assim, as vezes eu falo com as meninas assim, que aí começa tudo de novo, é a terceira infância, porque a fulano, com isso, aquilo, é aquela coisa né tá sempre na dependência, quase igual uma criança, a gente, a gente tem que ver isso, todas essas coisas.
