# Supplementary material for: Care for older adults with disabilities in Long Term Care Facility
Source: Rev Bras Enferm. 2023 Dec 8;76(Suppl 2):e20220767. doi: 10.1590/0034-7167-2022-0767 (PMC10704689; doi:10.1590/0034-7167-2022-0767)
Supplement: 0034-7167-reben-76-s2-e20220767-suppl05 [file 0034-7167-reben-76-s2-e20220767-suppl05.pdf]

E16

1) Pesquisador 2: **De quem foi a decisão de você vir morar aqui? Por quê?**

EI 16: Minha mesmo, própria. Opção própria

*Pesquisador 2: Por quê?

EI 16: Devido à eu não ter família, condições financeira né, não ter saúde pra sobreviver sozinha né, um salário pouco, um salário mínimo e que aqui dava condições que eu pudesse vir pra cá né?!

2) Pesquisador 2: **Como é o seu dia a dia aqui? Como é, para você, morar aqui?**

EI 16: O meu dia a dia aqui eu estou no bem bom! No momento, a única coisa que eu estou fazendo é beber, comer e dormir, até que dor... comer eu não tô comendo muito bem não porque eu tô com pouco apetite não sei porque, se é alimentação. Agora no dia a dia a gente pode ... com as pessoas, sou um pouco assim estre... não é estressada, as vezes eu quero ser uma pessoa boa, ver o mundo com justiça, essas coisas que eu sinto bem. Aí faze conta que tá tudo bonita, não está. Isso eu não concordo.

*Pesquisador 1: Mas...

EI 16: E aí eu questiono isso e sou muito mal interpretada. Eu mesmo... tento me policiar pra achar que tá tudo bonito, a mil maravilha e não está.

*Pesquisador 1: É aqui dentro da casa que a senhora...

EI 16: Não, é no dia a dia de todos nós, nós estamos vivendo um momento assim que as pessoas, não é questão, é, é uma violência assim, que não é de agressão assim, mas a gestic... gesto, uma palavra assim, não tão assim com boa vontade, uma expressão, tudo isso, é no dia a dia. Mas na casa aqui, pelas todas condições, pelas limitações que a gente vê é uma casa pobre né?! Com dificuldade, é convenio com a prefeitura, é colaboração com outras pessoas pra manter porque nosso valor é simbólico, o que a gente paga aqui. É muito bom aqui e muito bom. No dia a dia você vê aí fora, mas aqui eu vejo as coisas muito boa e acho que as pessoas aqui, principalmente os cuidadores, eu tiro o chapéu, pelo espaço físico deles trabalharem, deixa a desejar, mas também a gente vai ver as condições e está difícil, está uma crise e a Igreja também não pode também dar um conforto, porque é uma casa antiga não é, que não é adequada pra ser uma casa de idosos, mas se tornou uma casa devido as pessoas viverem mais e também não tem como deslocar

daqui pra outros lugares. E aqui é muito bom, com todos os altos e baixos a gente se diverte... se diverte mesmo, se você ficar aqui, um dia assim... igual esse dia que tá chovendo o pessoal tá mais tranquilo, mas na hora dum lanche, na hora do almoço aqui não parece um asilo. É muito interessante isso, tem musica né, tem os palhacinhos que vem aqui, é o pessoal da Igreja Santo Antônio que vem aqui comemorar os aniversariantes do mês né?! Então é uma casa bem movimentada, de visitas e a parte de lazer, eu gosto. Mas no dia a dia a gente tá vendo que o mundo está nada assim, mas ainda tem muita coisa boa, só que divulga o que há de pior né?! Mas ainda tem as coisas boa que ainda faz a diferença, e aí que a gente tenta. Eu mesma tô me reeducando e tentando vê ainda se eu melhoro alguma coisa em mim mesmo, não é?! Porque eu tenho que melhorar em mim pra ver no outro.

*Pesquisador 1: Verdade!

EI 16: Tem as vezes na primeira assim, interagir mesmo, se eu não tô bem vou para o quarto, fico lá, ligo o rádio, que a minha paixão é música, a minha paixão, o que eu amo ainda na vida, é a música. Música, a arte de apresentar, tudo eu gosto. Só não sei tocar! Mas agora no momento, eu trabalhava como... até 2016 eu acom... eu acompanhei, eu não era acompanhar, dava plantão ali no Otaviano Neves, ali tem uma ala dos acamados que vocês devem conhecer lá né?! No Otaviano Neves, 3 andar?! 3 andar é a ala dos acamados, com traqueo, com gastro ou então pequenas cirurgias que fazem ali. Fiquei ali 2 anos e 6 meses trabalhando a noite, até que é... 11 de maio de 2016, é uma senhora que tem traqueo e gastro da minha idade, chama Isabel, eu dava plantão noite sim e noite não. Pra mim era até interessante ir pra lá, primeiro que tive aquele impacto de ver o pessoal com traqueo e gastro que eu, eu não tenho curso de técnico de enfermagem, eu simplesmente, que tem os técnicos ali pra dar, pra ajudar a fazer o trabalho deles. Então ficava ali pra uma emergência, chamava um técnico, mas fazia alguma coisinha, era uma limpeza, uma higiene, umas coisas assim, mas acabei até aprendendo porque ela era muito secretiva, acabei aprendendo pelo menos o mínimo, aspirar, não dentro daquilo que eu podia fazer, que eles me orientavam né?! Fazer aqueles pequenos curativos, que as vezes era na gastro que sabe aquelas.. a gazinha, isso até que fazia, medicação era deles, dar uma água, coisa assim. E depois a minha amiga adoeceu que morava em um asilo, no, ali no bairro Itapuã, no Planalto, e ia morar no asilo, quando ela adoeceu eu sai do, do Otaviano Neves e fui acompanhar a minha amiga que veio a falecer em janeiro do ano passado, porem, depois que ela faleceu, eu comecei a ficar doente e hoje em dia eu estou

num proc... eu comecei a ficar com problema de queda de potássio e sódio, e fiquei internada 8 dias e tive uma convulsão, eu não sei eles que conta pra mim. Fiquei 8 dias ali no Luxemburgo e potássio, desidratação, sódio, tanto é que eu fiz um exame ontem, mas nã... então eu tirei esse período, um ano de dois mil, assim depois que a minha amiga faleceu, eu estou assim. Faço uma palavra cruzada, leio um livro, uma coisa assim, mas tô pedindo a Deus que eu possa melhorar porque eu não ficava dentro de casa, eu não! E eu senti, na hora que eu parei e a, e a minha amiga faleceu, eu ten... macho que eu morri uma parte assim que ainda, parece que foi um bloqueio e eu... tô sentindo até hoje ainda, mas no momento eu estou de... como que eu falo assim com, eu tô de vagabundisse, é... mas eu gosto da leitura, gosto de uma leitura eu gosto, ainda gosto da leitura e da música.

2) Pesquisador 2: **Como é, para você, morar aqui?**

EI 16: E aqui é muito bom! Aqui é muito bom porque nós não fica presa, aqui que eu não tô podendo sair todos os dias com esse problema agora que eu tô, não sei se é bem uma labirintite, mas se eu tivesse bem tava medindo a rua... é, bolsinha atravessada aqui. Não tem, é quando você tá independente, aqui é uma beleza, dando satisfação: tô saindo, volto tal hora, tem uma liberdade aqui que é até difícil. Eu falo que se a gente aqui deixar, deixar de existir, agente não vai termos essa liberdade que a gente tem aqui não. Tem muita liberdade, o café fica lá pra tomar a hora que quiser, não tem aquele negocio de um pãozinho que vem, não, não, a gente pega o pão tá mesa, você serve um leite, serve um café, pão, tudo é muito, acho que nem na casa da gente, agente tem tanta liberdade igual tem aqui. Tem as pessoas que tem suas limitações e a gente tá ainda com um pouco de independência, a gente pode fazer essas coisas, tanto é que eu fiz esse trabalho até 2016. Deixei pra acompanhar a minha amiga e depois que ela faleceu que eu fui ficar doente... mas espero que fique bem que ainda precise fazer alguma coisa. Nós pegamos uma água, atender uma porta assim no sábado que não tem o pessoal, atender qualquer uma atende né?! Tem que ter, tem que se identificar, por exemplo, mas aqui é muito bom. Aqui assim, eu tô sendo muito sincera com você, tem uns que tem essas coisas assim, tem o terço, tudo, tudo, tudo que cê pode pensar, teve o Carnaval, o Carnaval nosso foi meio esquisito sabe, mas foi alegre, foi interessante, vai ali liga um som, mesmo as cadeirantes tem umas que sabem as musicas de marchinha de carnaval, foi uma beleza, é muito interessante aqui. Aqui a gente não sente assim aquela tristeza, marasmo, tanto lugar que cê vê as pessoas com aquele semblante triste, apático, não. Eu estou sendo sincera com você porque eu conheço outros asilos, quando eu fiz curso de cuidadora né?! Fiz depois de

velha quando o Brasil acordou tarde para o envelhecimento do, do país e eles começaram a investir, fazer esse curso de cuidador que muitas vezes faz o curso de cuidador e não tem assim, a dimensão que significa assim a palavra cuidador né?! E aí eu fiz o curso foi na época, foi em noventa e... que começou realmente mesmo a divulgar foi a partir de 99. Conheci uma menina que foi presidente nacional do Idoso, na época do Fernando Henrique, não sei se ela ainda tá viva, Rita Félix, convivi muito com ela e ficava sabendo assim, das dificuldades, necessidades dos idosos no interior.

4) Pesquisador 1: **Agora, me fale sobre seu relacionamento com os outros idosos que moram aqui.**

EI 16: Tem umas pessoas que eu gosto mais, eu sou muito ligada aqui... pelos indefesos. Porque quem é... os normais, não precisam de estar aqui não. Eu sou um pouco polêmica, não vai pensar você que, soouuu!!! Iiihhhh! Não queira saber, mas eu gosto mais é dos cadeirantes. Da Altina, vocês já fizeram entrevista com a Altina, aquela que é cega? Tem a Altina, é... a Fia, esses sim. Eu acho que tem uma palavra, por exemplo, uma água, poder pegar uma água, sirvo um café na hora que tá todo mundo, plantão da Graci, por exemplo, ela não deixa a gente, eu respeito, não é?! Mas no outro assim se puder servir, eu sinto bem de servir um café. Eu acho assim que eu não tenho o direito de sentar ali, eu não concordo, é isso que me atrapalha, nesse sentido. Eu sentada lá, tomando um café enquanto tá uma cadeirante ali aguardando uma cuidadora, uma técnica, não acho justo, entendeu?! Meu jeito de ser é um pouco es, eu sou polêmica, toda vida não é agora, de direito, de igualdade, de justiça, tudo isso eu questiono.

3) Pesquisador 1: **Me fale um pouco sobre seu relacionamento com as pessoas que trabalham aqui.**

EI 16: Ah isso aí é di, di, di, di (inaudível) muitas vezes e depois volta às boas. Uma fala uma coisa, a outra fala outra, não é pra fazer e eu falo, e vai...

*Pesquisador 1: A senhora entra em atrito, às vezes?

EI 16: Xiiiii!! Diz que quando meu cabelo levanta, não assim, será que quando ela levantou o cabelo e isso. Eu sou polêmica, toda vida eu fui, não é agora, mas querendo achando que a coisa, gosto da justiça, gosto das coisas muito certas. Não vai pensar você que eu não compro uma briga assim de sensibiliza com, não é uma briga em si, mas a gente vê às vezes... no dia que eu presenciei a... cuidadora um pouco rígida assim, áspera

com uma idosa, eu não me senti bem. Cê sabe que de vez e quando tem esses casos, não adianta que todo mundo sabe que tem né?! Eu, por exemplo, lá no Otaviano Neves, eu fazia um plantão que os dois técnicos eram... eles reclamavam direto deles, na diretoria. A aspereza com que eles pegavam o idoso pra trocar e tudo, eu mudei de, então, eu, é eu, eu pedi a outra, e a outra fazia o outro plantão, falei assim: olha eu abro mão de dois plantões pra você trocar comigo porque eu tô precisando que, mentira eu não tava fazendo nenhum trabalho, só pra não presenciar a forma com que eles...

*Pesquisador 1: Mas a senhora acha que isso acontece aqui, essa aspereza, às vezes, por parte do cuidador?

EI 16: ... (silencio) eu acho que onde tem humano, ser humano, nós não somos diferentes... né?! Às vezes tem hora, que tem momentos que a pessoa está ou preocupada com algum problema, tá estressada, que é o transito, que é o filho, que é o marido, uma coisa assim, cê sabe que o idoso é uma coisa difícil, eu sou, eu acho, não vou falar que eu sou, que eu me posiciono assim, todos são difíceis. Tem uns mais flexíveis, mas o trabalhar como cuidadora ou cuidador, ele tem que pensar duas vezes se ele quer realmente exercer a profissão porque ele tem de que se posicionar no lugar daquele que está precisando, se é que ele queria de ser tratado daquele jeito. Ele tem que saber disso, um tom de voz, um bom dia, porque a pessoa sente né?! Se você der um bom dia afável, amigável, você passa aquela firmeza, aquela sinceridade, aquela segurança pro outro, às vezes cê vê pessoas assim que dá um bom dia e parece que ele, nossa mãe, aquilo nem, nem, não sabe nem, pelo menos manter aquela, porque a pessoa sente, mas tem aquelas pessoas boas. Nós tivemos aqui na casa uma pessoa maravilhosa que veio a falecer, um eletricista né?! Aquele eu senti, aquele tinha assim, sabe aquela pessoas assim, aquele anjo, uma paz dentro de si, isso é espontâneo, que eles pensam que a pessoa boa, é, é não, bondade não confunde com o que ele é. Eu senti! Eu senti a morte do Ailton viu, porque ele era carinhoso com as idosas, não, e independente disso era a pessoa dele, não adianta eu falar assim, eu faço isso, eu faço aquilo, isso não tem nada a ver. Aí você é prestimosa, você não é?! È uma pessoa solidária, mas uma pessoa boa de um anjo, aquela luz, é diferente. A pessoa irradia isso é diferente não é?! E, é isso eu olho muito nas pessoas, eu sou muito observadora, eu sou bem observadora ne?! Nesse problema que eu tive lá no Otaviano Neves eu não falei, ela falou assim ah que você mudou de plantão porque, não, eu tô fazendo um outro trabalho que porque tinha dois técnicos que, eu preferia o outro plantão porque tinha dois técnicos que eram mais profissionais. Passou um filme muito

antigo que tinha uma, dois, quatro técnicos da noite, não sei se vai, não sei se é hoje porque eu afastei e essa senhora, tem 4 anos que ela tava internada com traqueo e gastro. E a gente conhece todos os cuidadores, o tempo que a gente fica aqui, agente fica conhecendo os cuidadores dos outros plantões porque pra todo lado tem cuidador, um acompanhante né?! E aí eu falei, não, eu prefiro é abrir mão de dois plantões para estar no outro, mas existe aquelas pessoas que tem o dom, tem o mesmo assim que não seja carinhoso, que seja só profissional não tem importância não, cuidando bem direitinho e tudo, pro cê cobrar nada não

5) Pesquisador 2:**Você mantém contato com outras pessoas de fora da Instituição. Se sim, com quem e que tipo de contato é esse?**

EI 16: Eu também sou preguiçosa e sou enrolada, mas me conhece. Tem é uma que quando eu ligo pra ela assim. Mas você demorou, eu digo ahhh, aí eu enrolo, enrolo que é danado. Elas já me conhecem, mas são amizades assim, amizades de 30 anos, de 20 anos, a minha ex chefe tem 40 anos. Cê sumiu Gilberta, ahh eu não sumi, daí elas já, todo mundo já sabe que eu sou enrolada, mas são amizades verdadeiras. Que a minha ex chefe o que ela tem que falar ela fala, é uma amizade transparente, uma amizade sincera. Aquela amizade assim que se puder ajudar e tudo.

*Pesquisadora 1: E a senhora vai encontrar com eles ou eles vêm aqui na casa?

EI 16: E quem disse que eu gosto de receber pessoas, eu não! Assim, eu falo que se eu tivesse casa, eu ia comprar copo descartáveis e olha lá e servir só água. Eles morre de rir de mim. É porque eu ia, dona de casa, praticamente eu fui sozinha né?! Minha irmã casou, minha mãe faleceu, éramos sete irmãos né?! Então morei com meu irmão, com meu pai, então era uma dona de casa que cuidava das coisas, ihh, fazia café pra visita, que antigamente era assim, se chegasse na hora do almoço, se fosse pra almoçar normalmente o pessoal almoçava muito nas casas antigamente, agora tem self service, os restaurantes eram pouco, as amizades eram assim, eu almocei agora vou fazer o lanche da tarde ou ia almoçar e ficava ali o dia todo. Então assim, eu não quero saber mais disso não. Agora eu tô descansando do ferro, de passar roupa, de organizar a casa, essas coisas. E tinha que estudar a noite né?! Porque meu curso eu tive que fazer a noite e fiz depois de velha né?! Estudei depois de velha, e pobre na minha época era difícil de estudar, meus professores eram médicos, era difícil ou então era assim... formado na UFMG todos eles e da UFMG. Tinha um que era muito engraçado de biologia, era Dr Romildo Mendes que Nossa

Senhora, esse era terrível. Bunda era malfada, Mafalda, apelidou malfada o nome, pra falar não tem importância não né?! Ihhh, tá gravando? Ihh esqueci. Então ele era terrível, médico, ginecologista, você não via pobre dando aula pra gente não. Doutor Francolino era dentista, tinha especialização, estou na, fez um curso na Rússia, quase todos eles era assim, eram famílias tradicionais que dava aula pra gente, não tinha pobre dando na escola normal, porque eu estudei no Estadual e não tinha feito nem o primeiro nem o segundo grau.

6) Pesquisador 2: **Você se sente em condições de tomar decisões sobre as coisas que precisa fazer no dia-a-dia? Por quê?**

EI 6: As independente é ainda toma, mas tem a parte, agora cê sabe que teve a fiscalização veio e nenhuma de nós ficou com a medicação no quarto. Eu acho que eles tão até certo que eu não tô esquentando muito a minha cabeça não, por causa de remédio. Elas às vezes me falam assim: oh, tá faltando essa medicação. Isso aí eu acho certo mesmo, acho que elas tão certas...

*Pesquisador 1: Você acha que são só as independentes que conseguem tomar decisão sobre as coisas do dia a dia?

EI 16: É porque essas outras aí, por mais que elas queiram, mais aí é a parte da família quando a família assume, e as outra é a própria instituição né?! Mas é muito bem assim, eu acho as decisões aqui muito boas, eu gosto do sistema de trabalho, porque tem essas que não tem família. Não pode citar nome não né?! É porque tem a Dora que não tem família, então eles que assumem toda a assistência pra Dora, tem... acho que praticamente é Dora, são poucas né?! Aquelas que realmente não tem família, aquelas que têm família e que são responsáveis, elas que assumem assim, a medicação, a assistência,

*Pesquisador 1: Mas e as idosas?

EI 16: A idosa que você fala em que sentido?

*Pesquisador 1: Ela consegue decidir por exemplo o que ela quer comer, horário, se ela quer tomar banho?

EI 16: Não, os horários são programados senão elas vão ficar o dia todo né?! Por aqui tem uma. O café da manhã a partir de 7 e meia, mas nem todos, as vezes, as dificuldades que tem, poucos funcionários, muitas que precisam de ajuda na hora do banho. Então atrasa, na parte de medicação, mas o que se pode fazer dentro do possível, que os horários

que se tem de, de, de refeição, tem de agilizar mais as coisas né?! Agora quanto a alimentação de quem tem controle, é seguido rigorosamente por que no caso dessas que tem diabete e outras coisas mais, tem que ter um controle de alimentação, faz certinho também né?! Segue a risca, isso aí segue a risca e... nós temos a nutricionista, mas se a pessoa quer comer uma coisa diferente ela pode ter liberdade, principalmente nós independentes, a gente assim, mas as vezes elas fazem coisas assim que pode agradar a todas assim. Normalmente a pessoa gosta de doce, eu sou avessa a, avessa a doce, então... não sinto falta. Mas aquelas que pode e elas fazem sabe?! Quando sai um pouquinho do cardápio, alguma coisa diferente, faz. Aqui tem muito isso, aqui é muito bom, o dia a dia, a nossa alimentação. Eu não almoçava, povo no Otaviano Neves falava almoça aqui, eu falava: eu não! Eu vou deixar minha comidinha lá pra almoçar aqui essa comida sem tempero, mas nem vê aquela comida. Aqui eles seguem a risca né?! Aqui tem nutricionista, mas a comida é muito temperada e gostosa. Eu gosto muito daqui. Nós não vamos, eu falo com a Nercina assim: nós não vamos caso .... tem geladeira no quarto, minha televisão tá velha, porque, porque eu não gosto, a digital não pega a TV cultura de São Paulo, porque aqui não pega, a parabólica pega aqui, então eu sou ... eu pego a TV cultura de São Paulo, que eu gosto do jornal, da TV cultura. E tem a TV pras idosas assisti a missa sabe?! Tem tem tudo. Assim essa parte é muito bem assim, aqui pela casa, pelas condições da casa é muito boa. Televisão a vontade, fica lá ligada, direto, se elas quiserem assistir, assiste...
