# Supplementary material for: Care for older adults with disabilities in Long Term Care Facility
Source: Rev Bras Enferm. 2023 Dec 8;76(Suppl 2):e20220767. doi: 10.1590/0034-7167-2022-0767 (PMC10704689; doi:10.1590/0034-7167-2022-0767)
Supplement: 0034-7167-reben-76-s2-e20220767-suppl06 [file 0034-7167-reben-76-s2-e20220767-suppl06.pdf]

EI 17

1) Pesquisador 2: **De quem foi a decisão de você vir morar aqui? Por quê?**

EI 17: Hum?

*Pesquisador 2: De quem foi a decisão de você vir morar aqui?

EI 17: Eu, porque aí eu tava morando em São Paulo, aí voltei, já voltei doente, eu trabalhava muito a noite, né, fiquei com problema de cabeça, fiquei internada umas três vezes no hospital psiquiátrico, que eu trabalhava de dia e de noite e minha chefe também trabalhava de noite, aí não aguentei, fiquei esgotada, trabalhar de dia e de noite. Aí dentro de um ano, eu fui operada três vezes, né, aí eu não tava aguentando mais trabalhar em hospital que era serviço pesado, aí meu medico psiquiatra arrumou pra mim vim embora, aí quando eu vim embora, já fiquei morando, ainda voltei pro emprego, depois quando eu não dei conta mais pagar aluguel, comprar coisa né, comer, aí a gente já tinha a casa aqui, depois a gente vi, vem pra cá.

2) Pesquisador 2: **Como é o seu dia a dia aqui? Como é, para você, morar aqui?**

EI 17: É bom, né muito ruim não, mais agora a pouco tempo pra cá minha cabeça boa não.

*Pesquisador 2? Como que, o que a senhora faz durante o dia?

EI 17: Antes que fui operada da vista, de glaucoma, né?! Eu tava fazendo fuxico, fazendo minha costurinha, assim tapete e tudo, aí depois que eu fui operada da vista, operada da vista duas vezes de glaucoma, e essa de catarata, aí minha vista agora tá embasando muito, eu não tô dando conta de costurar, de fazer minhas coisas tudo, não tô dando conta, agora esses dias que minha cabeça não tá muito boa, por causa dos remédios que eu tomo, tô dormindo muito, aí durmo de manhã, depois do café, depois do almoço, só vou lá fora pra almoçar, volto e deito outra vez, eu sinto não tá tendo pessoa pra ajudar a gente, teve muitos estudantes aí né, mais agora nas férias tá parado, mais não tô dando conta de estudar muito não, eu tô fico muito parada no quarto.

*Pesquisador 2: E quando tem a fisioterapia a senhora participa? Quando tem as atividades?

EI 17: Eu participei três vezes, agora não quero mais, agora eu quero é artesanato.

*Pesquisador 2: Ah, senhora quer fazer artesanato agora?

EI 17: Ah eles ficam mandando a gente muito, né?!

*Pesquisador 2: E como é, pra senhora, morar aqui?

EI 17: É ruim não, é bom, que antes eu ia à missa, rezava muito, já depois dessa confusão de carro aí, o médico não deixou a gente sair mais.

*Pesquisador 2: Aí a senhora não sai mais da casa, não?

EI 17: Sozinha não, só com acompanhante.

*Pesquisador 2: E a senhora costuma ir à missa ali na igreja, da Boa Viagem?

EI 17: Antes de ontem eu fui à noite, mais de vem quando o padre vem aqui primeiro sábado, aí eu assisto a missa.

*Pesquisador 2: Aqui na capela, né?!

EI 17: É.

3) Pesquisador 2: **Me fale um pouco sobre seu relacionamento com as pessoas que trabalham aqui.**

EI 17: Eles maltratam muito a gente.

*Pesquisador 2: É?

EI 17: A única coisa que me maltrata muito, tira muitas coisas da gente, coisa que a gente guarda, coisas que a gente ganha, só esse mês pra cá tive tanta decepção, eu ia viajar, tirou o, quando eu não tava no quarto, tirou meu dinheiro, viajei aborrecida, com meu sobrinho lá perto de Mariana, umas coisas minha que eu tinha de estimação, me tirou, eu tô muito amolada.

*Pesquisador 2: É?

EI 17: Eu choro muito.

4) Pesquisador 2: **Agora, me fale sobre seu relacionamento com os outros idosos que moram aqui.**

EI 17: Minhas companheiras?

*Pesquisador 2: É.

EI 17: Eu não tenho, não tenho queixa não.

*Pesquisador 2: A senhora tem alguma amiga na casa?

EI 17: Amiga é difícil né?! Porque cada uma tem seus quarto né, quem não tem nada pra fazer vai pro seus quarto e elas vão ver televisão e eu vou pro meu quarto, de noite vejo televisão também, as coisas que tão aparecendo religioso, né?! Aí fico assistindo até na hora de dormir.

5) Pesquisador 2:**Você mantém contato com outras pessoas de fora da Instituição. Se sim, com quem e que tipo de contato é esse?**

EI 17: Com quem?

*Pesquisador 2: Se a senhora tem contato com alguém fora da instituição.

EI 17: Ah, eu tenho amiga, né?! Tenho sobrinho, tenho uma irmã, meu pai, minha mãe morreu, nós ficamos no emprego lá no Sesita, aí a mais nova foi pro Rio, e eu vim pra Belo Horizonte com uma amiga minha. Eu tenho amizade assim, tem meu sobrinho que fica me procurando, são três irmãs, uma veio aqui me procurar, eu já encontrei com ela duas vezes, fui lá, me levaram no Rio pra encontrar com elas e encontrar com as filhas dela, porque não podia vim todo mundo aqui e a outra eu ..... fugiu, rs, e a outra minha sobrinha tem uma filha de cinquenta ano que fica me procurando.

*Pesquisador 2: Eles vêm aqui visitar a senhora?

EI 17: A minha sobrinha vem.

*Pesquisador 2: Eles moram aqui?

EI 17: Mora, mora em Uberlândia, agora vão mudar pra cá.

*Pesquisador 2: E as amigas da senhora, vem visitar a senhora, também?

EI 17: É muito difícil, né?! todo mundo trabalha, muito difícil.

6) Pesquisador 2: **Você se sente em condições de tomar decisões sobre as coisas que precisa fazer no dia-a-dia? Por quê?**

EI 17: Hein?

*Pesquisador 2: Senhora se sente em condições de tomar decisões da vida da senhora aqui dentro da casa?

EI 17: Eu não tenho não, porque...

*Pesquisador 2: As coisas que a senhora precisa fazer no dia a dia, a senhora consegue tomar decisão?

EI 17: Agora esses tempo, eu não tô não, teve um problema do dinheiro que eu ia viajar, a cuidadora pegou o meu dinheiro, escondido de mim, levou meu dinheiro, quando eu fui procurar o dinheiro e pôr na bolsa, pra mim viajar, não tinha nada. Aí quando a gente, quando ela voltou, no dia deu viajar, a gente teve discussão, agora a gente tá conversando não, era quatrocentos reais, depois ela me deu duzentos, num dava pra eu viajar né, eles também não ia ficar sem dinheiro pra viajar, nos fomo de carro. Aí o outro agora teve uma conversa aí e tudo com a Vanessa, com as companheiras, que eu tomava conta do eu dinheiro, agora não posso tomar conta mais, aí tá aí no escritório. Quando preciso pagar as coisas tem que pedir dinheiro a ela.

*Pesquisador 2: E assim, a senhora consegue tomar decisão no horário que a senhora quer comer, tomar banho, essas coisas a senhora se sente em condições?

EI 17: Tomo.
