# Supplementary material for: Care for older adults with disabilities in Long Term Care Facility
Source: Rev Bras Enferm. 2023 Dec 8;76(Suppl 2):e20220767. doi: 10.1590/0034-7167-2022-0767 (PMC10704689; doi:10.1590/0034-7167-2022-0767)
Supplement: 0034-7167-reben-76-s2-e20220767-suppl07 [file 0034-7167-reben-76-s2-e20220767-suppl07.pdf]

EP 10

1) Pesquisador 2: **Como é, pra você, trabalhar em uma ILPI?**

EP 10: Pra mim é muito bom, né?! A gente sente assim, pra mim é um trabalho muito gratificante, né, trabalhar com idoso, com pessoas que mais precisa, que aqui é uma obra que, com pessoas mais carente. Então pra mim isso é uma gratidão, estar aqui, sinto muito feliz, estar aqui com as idosas.

*Pesquisador 2: E antes da senhora vir pra cá, a senhora chegou a trabalhar em outra casa de idosos?

EP 10: Trabalhei sim, sempre cuidei de pessoas doentes e idosas. Trabalhei numa ILPI 2006 a 2012, na região de Raul Soares, na cidade de Raul Soares, lá já era coordenadora mesmo, já tinha minha carteira como coordenadora da casa.

2) Pesquisador 2: **Me fale um pouco sobre seu relacionamento com os idosos que residem aqui.**

EP 10: Pra mim é muito bom, é, a gente se sente assim, feliz de estar no meio delas, né, elas são pessoas muito alegre, as aqui são alegres, são idosas graças a Deus sente em casa, sente feliz. Bem no inicio que chega é um desafio pra adaptar, mas com o tempo elas passa a sentir se em casa, né, e a gente também se sente em casa com elas, porque a gente reside aqui, 24 horas, mora aqui, temos nosso quarto, quartinho ali, só uma reserva pra gente a noite, tá descansando, mas durante o dia tá com elas aqui, pra mim é muito bom. Alegria, rs.

3) Pesquisador 2:**Qual a sua percepção sobre a relação dos idosos institucionalizados com seus familiares e amigos?**

EP 10: É assim, pra mim também, elas se sentem muito bem também em tá acolhendo a gente, sente tá acolhendo bem as pessoas que vem aqui visitar, amigos e elas também, se sente bem, elas sempre falam né,” volte outra vezes”, quando a pessoa vem visitar lo ou vem aqui aí elas sempre tá convidando pra voltar mais vezes, né?! Não sei se é isso que você...

*Pesquisador 2: Elas recebem muitas visitas?

EP 10: Recebe, graças a Deus.

*Pesquisador 2: Dos familiares?

EP 10: Quem tem familiares, né, vem sim, de vez em quando vem, agora tem umas que não tem, tem pessoas aqui que não tem ninguém na família, né, só mesmo os amigos, voluntario que vem aqui e nos aqui com as funcionárias.

*Pesquisador 2: E a senhora acha que elas sentem falta desse contato, com a família?

EP 10: Eu acho que sim, isso faz falta, mesmo que a pessoa não é lucido, mas a gente sente que eles precisa desse carinho, da família.

*Pesquisador 2: Elas falam alguma coisa?

EP 10: Não, a que tem aí, que não tem família não consegue falar não, a gente percebe assim, que elas são, quando a gente chega perto dela, ela quer um abraço, a gente percebe que a gente não tem a família, que o abraço de alguém, né?! Então a gente se sente isso, agora a Maria das Dores é uma que não tem, ela foi, uma que foi institu... essa Associação da Toca de Assis que pegou ela na rua e encaminhou ela pra aqui, né?! Então ela é uma que não tem família, até hoje não descobriu ninguém na família, tem assim, teve família, na história dela, né, teve até filhos, né, mas não tem contato, não sabe como que. Então isso, pra mim é muito triste, né, a gente tenta fazer o máximo por ela, carinho, a gente da o carinho, que sabe que precisa, que não tem a família pra dar, a gente tem que dar esse carinho, esse abraço, né?! Ela sente, que a gente tá chegando perto dela, manifesta que quer um abraço.

4) Pesquisador 2: **Você considera que os idosos dessa ILPI têm condições de tomar decisões sobre as coisas que precisam fazer em seu dia-a-dia? Por quê?**

EP 10: As que são lúcidas sim, né, porque é direito delas, né, pelo estatuto do idoso, a gente vê que eles têm direito, de se manifestar, de re..., de exigir, né, aquilo que for bom pra elas.

*Pesquisador 2: E a senhora acha que essa tomada de decisão tá relacionada com a lucidez?

EP 10: Isso.

*Pesquisador 2: Tá. E as que estão numa cadeira de roda, mas que ainda são lucidas, elas têm essa autonomia pra poder tomar decisão? Aqui dentro da casa, pra coisas da rotina.

EP 10: Não, tem sim.

*Pesquisador 2: A casa proporciona?

EP 10: É, a casa oferece isso né, a gente fica com elas mesmo 24 hora, a gente oferece aquilo que tá no alcance da gente poder ajudar lo, o que puder fazer por elas, a gente faz sim e elas ficam felizes com isso, né, mas também se você não fizer isso, né?! Colocando no lugar delas, é pedir uma coisa, exigir uma coisa e não poder conseguir aquilo, né, que vezes tiver, a gente sabe que tá no alcance, então é triste, né?! Eu acho assim tô sempre colocando no lugar delas, né, que se fosse eu que tivesse na cadeira com a minha, com toda lucidez, assim por exemplo, gostaria de tá, tenho filho, tem uma aí que tem filho, a dona Elvira , é que telefonar pro filho, quer comunicar com o filho, é direito dela, é um exemplo que eu tô dando, não sei se.. Então é direito dela, tá ligando pro filho, comunicar com o filho, então ela tem todo esse direito e a gente dá essa autonomia pra ela. E várias que são, que tão aí que precisa, dessa ajuda, a gente tá a disposição.
