# Supplementary material for: Care for older adults with disabilities in Long Term Care Facility
Source: Rev Bras Enferm. 2023 Dec 8;76(Suppl 2):e20220767. doi: 10.1590/0034-7167-2022-0767 (PMC10704689; doi:10.1590/0034-7167-2022-0767)
Supplement: 0034-7167-reben-76-s2-e20220767-suppl08 [file 0034-7167-reben-76-s2-e20220767-suppl08.pdf]

EP 11

1) Pesquisador 1: **Como é, pra você, trabalhar em uma ILPI?**

EP 11: Nó, a primeira palavra que vem na minha cabeça é desafiador né!? Desafiador que é conciliar... é a filantropia como uma proposta de uma política né?! Então assim, tem toda uma estrutura duma política que a gente tem que cumprir, mas tem toda uma questão aí de uma... de uma carência financeira mesmo que é um desafio muito grande. Então é desafiador... trabalhar em uma ILPI.

2) Pesquisador 1: **Me fale um pouco sobre seu relacionamento com os idosos que residem aqui.**

EP 11: Essa é a parte boa da história (risos), é a parte fácil na verdade né?! Não é fácil de gerir uma casa só com mulheres né?! Que somos... todas as moradoras mulheres e todas as colaboradas mulheres também né?! É... e é isso né?! Mulher com as suas peculiaridades né, com é... com as intrigas, com as conversas, enfim, mas a parte boa é isso, a parte gostosa é o contato com as idosas, com a sabedoria delas, com o jeito de cada uma de ser, é... muito gostoso essa relação. Eu falo que a gente até aproveita pouco né?! Eu falo isso muito com a equipe assim: falei, pô a gente tem que parar pra pensar que a gente tá numa instituição com um monte de idosinha, que a gente tinha tudo pra ter... ter um momento gostoso assim. De sentar, de escutá-las né, de acolhê-las aí na questão afetiva, mas às vezes a rotina e o dia a dia até... afasta a gente, apesar da gente ter muito contato né, por ser uma casa, então todos os dias a gente tem muito contato com elas assim. É, eu gosto muito... Pra mim é novo também, eu não sou da Política do Idoso na verdade, é um desafio novo né?! Eu, eu começo minha trajetória acadêmica tendo muito contato com a Saúde Mental né?! Eu venho desse processo da Saúde Mental, então estagiei dois anos no PPJ. Não sei se cês conhecem o programa?!

*Pesquisador 1: Não

EP 11: É um programa do Tribunal de Justiça pro é... pro portador de sofrimento mental judiciário. Então assim minha, minha trajetória acadêmica, ela vem muito marcada com essa coisa do judiciário e da saúde mental né?! Estagiei no Galba, no Raul, então tinha uma trajetória toda pra, pela saúde mental. Aí num determinado momento, eu formo e vou morar no Vale Jequitinhonha, então vou ter uma experiência aí com a miséria no Vale do Jequitinhonha. Literalmente né?! Porque acho que é muito isso que eu vou

buscar também assim né?! E... e aí quando eu volto, é... eu vou ter um, um, uma trajetória aí pelo, é... eu trabalho numa unidade de semiliberdade com adolescente infrator, então já vou lá pra, pra segurança e tem um, um, um trabalho lá. E aí veio a Política do Idoso, então assim, é um momento também deu entender um pouco assim, o discurso da Politica do Idoso que é uma política que tá sendo construída na verdade. Né, então eu falo muito isso com as meninas assim, quando eu chego, mas qual é o olhar né que a gente tem que dar pra uma ILPI? Pro idoso aqui? Que isso lá, por exemplo, na Ceme, no discurso aí da SEDS da Politica da Segurança, tá muito definido né?! Que que é o ato infracional pra adolescente, que lugar que ele responde quando ele atua. Então é isso que eu fico tentando o tempo inteiro elaborar com as meninas né, que olhar que é esse que a gente vai dar pra casa assim. Então hoje a gente tá num processo muito de entender também, assim, de tentar trabalhar, muito a qualidade e autonomia numa ILPI, que eu acho que é um ponto mesmo assim, de todas eu imagino! Que é tentar ser uma casa, mas uma casa também que a gente vai, vai conseguir permitir, garantir ou auxiliá-las nesse lugar aí também né?!

3) Pesquisador 1: **Qual a sua percepção sobre a relação dos idosos institucionalizados com seus familiares e amigos?**

EP 11: ... (silencio) Pois é... vamos pensar assim uma coisa. A gente tá num momento da Política Municipal do Idoso, assim, com essa nova gestão municipal né?! E automaticamente com uma nova gestão da assistência, e... de, de pensar hoje, com o crescimento aí demográfico mesmo dos idosos, assim, o que que a gente vai ter que fazer pra gente dar conta disso aí?! A gente tem 1% dos idosos de Belo Horizonte institucionalizados, eu fico pensando cadê os 99% né?! Que às vezes tem situação de vulnerabilidade, mas a gente nem chegou neles ainda né?! A gente tem uma fila gigante na Prefeitura, pra acolhimento de idosos de grau III. Grau I e grau II nem tanto, mas que grau I e grau II ainda dá pra, pra vincular aí com a família. Hoje a gente tá num momento da Política que eu vejo muito o secretário falar, é... da gente pensar... em reforçar a, a, a capacidade protetiva das famílias né?! Então, pensar aí nesse bum do idoso, não como ILPI, como uma, uma, uma, uma única resposta pra isso que tá crescendo que a gente não vai dar conta. Senão a gente vai construir um milhão de ILPI´s aí né?! A ideia é isso, é reforçar a capacidade protetiva, lá na, na, na proteção básica né?! E aí a gente já diminui o público pra média e pra alta complexidade porque aí cê tem que pensar... Se eu já tô na alta complexidade, se eu já tô numa instituição de

acolhimento, é porque já tudo que tinha que ser tentado pra não ser institucionalizado foi e já não funcionou, mas na verdade, uma instituição de ILPI faz a gente pensar muito assim, mas foi mesmo? Tentado tudo que podia ser pra não chegar ao acolhimento institucional? E muitas vezes a gente vê que não. Entende?! Então, por isso que o discurso hoje da secretaria é, é, é esse de re... reforçar cada vez mais a capacidade protetiva da família pra que talvez essa pessoa não venha a ser institucionalizada e não pensar talvez numa instituição, como uma instituição de longa perman... instituição de acolhimento como instituição de longa permanência, por exemplo. Hoje a gente tem o nome de ILPI mesmo, é longa permanência, entrou aqui acabou! So... sua possibilidade de reinserção familiar é quase zero depois que cê entra numa instituição de acolhimento, isso é realidade de todas as ILPI. Mas também pensar nisso ou pensar antes disso acontecer na verdade. Como? Vou te dar um exemplo assim que eu tenho hoje vivo na casa. A gente tem uma meta pra 2018 na casa que é uma coisa que não tinha, da gente avaliar mesmo, é... as idosas que tem potencial de reinserção social. Como? Então, nós vamos fazer visita domiciliar, nós vamos fazer reunião com os familiares, nós vamos, tá saiu, uma proposta é, é de trabalho com essas famílias. Então eu tenho, por exemplo, uma idosa hoje, que desde que eu cheguei, pra mim é uma idosa com possibilidade de reinserção. Por que que essa idosa tá aqui hoje? Porque essa família ainda é uma família que precisa de produzir, trabalhar fora pra, pra, pra sustentar a casa né?! Então, a... o, o filho e a nora. O filho que trabalha na verdade, a nora não trabalha porque a, a mãe dela também adoeceu e ela hoje, entre a sogra e a, a mãe, ela ficou cuidando da mãe. Até então ela cuidava da sogra, então nesse momento que a mãe adoece, a sogra vem pra uma ILPI e ela fica. Mas o que eu vejo, é que não é uma família constituída de um filho, é uma família constituída de vários outros membros e que talvez se a gente pensasse aí em juntar essa família né, e mediar essa relação pra pensar num arranjo familiar possível de incluir essa idosa ali, talvez até com uma divisão mesmo de tarefas, eu acho que seria possível. Então por exemplo, quando eu discuto com ele, que ela não gosta de morar numa ILPI, ela não é feliz aqui, eu vejo isso nitidamente né, e eu fico pensando, cê faz todo um projeto de vida, todo um projeto talvez pra sua velhice, principalmente, a cultura anterior a nossa que é um projeto de crio todos os meus filhos, pensando que meus filhos vão sim, cuidar de mim é, é nessa fase. A gente não, a gente já é tipo uma, mentalidade da gente é outra, a gente já sabe que filho é pro mundo, que é esse discurso de hoje né, que o filho tem autonomia e independência dele. Então cê não tem garantia de nada, que hoje ele tá aqui, amanhã ele tá morando fora, amanhã acontece alguma

coisa, enfim... a gente, eu acho que isso diminui cada vez mais esse pensamento. E aquela coisa também, de que hoje cada vez, é... essa capacidade financeira nossa com o tempo ela diminui, então cada vez menos a gente vai ter, é, é ou condição de colocar alguém pra auxiliar nesses cuidados, ou então paralisar a vida pra cuidar de pai e mãe, então acaba que a ILPI é um recurso, independente da classe social, as vezes assim. Às vezes é, é um recurso como dificuldade do manejo também da família, no cuidado é com aquele ente familiar, é, mas enfim... Ela quando eu discuto com, com o filho dela, por exemplo, a possibilidade de retorno, pra dizer disso assim, eu crio isso da minha cabeça, não que eu veja um potencial dentro da sua família, porque eu vejo ela infeliz aqui dentro, ela não gosta de morar aqui e ela tem família, e ela tem filhos, e ela tem uma casa que é dela, e ela tem um, um benefício previdenciário! Então o que que falta nessa família pra gente tentar ver esse arranjo? O discurso dele pra mim é isso, ele vai dizer assim: olha, eu luto muito na minha vida até hoje, pra dar uma vida digna pra minha mãe. E aí eu questiono pra ele o que que é dignidade pra ele né?! Eu começo esse trabalho, mas o que que é dignidade? Ele vai dizer assim, dignidade pra mim é ela ter o mínimo, é ter um lugar pra morar e ter um plano de saúde. Então hoje ele paga um plano de saúde da Unimed pra ela, por 800 reais. Entende?! Que é o que ele entende pra ele que seria uma vida digna pra ela, é ter um médico aqui, que é o domiciliar, que acompanha ela aqui, e talvez pra ela não é isso... Tenho certeza que ela trocaria isso pra ter um convívio maior com essa família.

*Pesquisador 1: Entendi.

EP 11: Entende isso que eu tô te falando?! Então é uma família que tinha um super potencial para isso, mas não tem financeiro. Então assim, ela é uma pessoa, por exemplo, que eu não sei qual recurso que a política podia criar por exemplo, mas teria total condição da gente pensar numa proposta dela voltar pra casa que seja aí, com estado, município ajudando financeiramente essa família. Entendeu?! Daria pra fazer esse arranjo. Agora assim, a casa é feita de todos os tipo de, de pessoas, igual eu falei, tem pessoas, tipo a Yara que escolheu morar aqui que tem um arranjo familiar super extenso, que tem um convívio familiar enorme e que a qualquer momento que ela falar, não quero mais ficar aqui, ela tem vários lugares pra ela morar mas é uma escolha dela mesmo, dessa questão da autonomia mesmo, dessa mulher que decide que vai ser feito com o destino e com a vida dela, como foi a vida inteira. E como eu tenho pessoas que não tem vinculo familiar nenhum mesmo. A maioria do meu publico, é um público que

tá numa ILPI porque não teria outro lugar pra ir...né?! Mas um outro arranjo também possível por exemplo. Eu tenho um publico que é de grau I, por exemplo, que é a Gilberta, a Nercina, a que a gente veio falando, que pra mim não é um publico que seria publico de uma ILPI, seria um publico de republica... entendeu?! Então elas são ativas, elas são pensantes, elas dão conta de se organizar, inclusive para fazer pra além das atividades diárias delas mesmo, pra pensar em, na organização de uma casa. Então, por exemplo, eu não acho que seria publico pra cá. Hoje elas dariam conta de morar numa república, cada uma ter um quartinho, elas se organizarem ali pra ajudar numa compra, numa feira, fazer uma comida, ajudar na organização. Visitei uma republica agora e há pouco tempo, super legal!

*Pesquisador 1: É?

EP 11: É! Mas que hoje a gente tem uma república em Belo Horizonte, então, nã... não ta aí por isso. E a ILPI fica mais pra um grupo que já vai ter aí um grau de, de dependência maior. Vai conciliar essa coisa com a família e a financeira. É... mas são públicos diversos assim que eu vejo na casa.

*Pesquisador 1: Você acha que esses parentes, familiares são participativos aqui dentro da casa?

EP 11: ... Sim. É muito assim sabe, acho que que toda pessoa que deixa um parente na ILPI, ele acaba se ausentando cada vez mais desse lugar da responsabilidade que ele tem com aquele ente. E aí a gente vai pensar o que? Esse laço que ele tem, dessa obrigatoriedade na verdade com esse ente, ele é imaginário, simbólico, que na verdade na lei, ele não tem. Então não tem nem o que cobrar. Eu tento pegar eles por outro lugar, porque assim quem vai ter mesmo na lei, na constituição essa obrigação com o ente é o filho com o pai. Eu vou te falar, a maioria aqui, não é nem filho que vem, é parente. É uma tia, é um sobrinho, é um neto, então assim, na lei eu não posso cobrar nada dessa pessoa, nada! A gente tenta ir por um outro caminho, de dizer, era, era uma convivência que cês tinham pra esse lugar, da importância da presença na vida mesmo emocional dessa pessoa do que na obrigação aí perante a lei, né?! Apesar da política ter todo aí um critério pra isso né?! A gente tem uma lista de presença, então todas as pessoas que visitam, elas têm que deixar o nome ali. E as meninas faz um controle das idosas porque durante a semana a gente até vê, final de semana não. Elas fazem o controle das idosas que estão sendo visitadas ou não, as idosas que não estão sendo visitadas, a gente vai

fazer uma análise disso. Não está sendo visitada por quê? Por exemplo, no caso da Gilberta, ela não recebe visita mesmo de parente, isso é uma coisa que ela coloca, ela fala disso né?! Eu não quero, sei lá. Eu chego aqui dessa forma e... é mais algumas não né?! Tinha família envolvida e em determinado momento, por um período quando a família afasta, a gente faz esse contato, a gente puxa orelha mesmo, a gente fala: oh vem, tá sentindo falta, né?! Por que não? Mas o que a gente percebe é que a partir do momento que tá aqui numa ILPI, a gente passa a, é... gerir tudo da vida desse idoso né?! Fica até cômodo pra família desse idoso, a gente tem médico aqui dentro, tem a equipe toda, as vezes um medicamento é comprado por aqui e hoje é um movimento que cada vez mais eu faço na verdade né?! Quando eu cheguei eu vi aqui técnica falando comigo assim, cê vai pegar o cartão dessa idosa, você vai administrar o dinheiro da idosa. E aí a tendência é que essa família venha cada vez menos, é uma possibilidade, mas aí eu acho que a gente tem que começar a criar laços com essas, com a família, que não seja por essa via né?! Então assim, por outra via mesmo. É vir aqui visitar ou ter um momento com essa idosa, não pela relação de, de dinheiro assim, não por esse dever, por outro lugar assim, que eu acho que a gente tem que conquistar esse espaço com essas famílias assim. É porque a família administrar o dinheiro pra mim, as vezes é muito mais problemático pra mim do que o contrário porque por exemplo o Dr Mauro veio ontem, que é o médico, vamos supor que ele prescreveu um antibiótico pra essa idosa. Um antibiótico não é uma coisa que vai dar pra esperar, um antibiótico é uma coisa que eu tenho que começar a dar imediatamente. Então às vezes eu vou ligar pra família pra eles trazerem a medicação e vai demorar dias pra mim e eu não tenho dias, e eu não tenho dias e às vezes eu não tenho dinheiro. Então hoje, o movimento que eu faço às vezes é tentar administrar mesmo o dinheiro da idosa por aqui, por que aí quando eu preciso tem uma demanda que eu mesmo consigo suprir ela, o mais rápido possível né?! Essa administração hoje é feita pelo escritório.

4) Pesquisador 1: **Você considera que os idosos dessa ILPI têm condições de tomar decisões sobre as coisas que precisam fazer em seu dia-a-dia? Por quê?**

EP 11: Alguns sim outros não.

4) Pesquisador 1: **Por que?**

EP 11: Aí vamos falar de grau, eu nem gosto de falar de grau porque aí a gente numera as pessoas né?! Mas pra gente entender. As idosas de grau I total, as idosas de grau I e

grau II algumas, total condição de, de, de responder é... pela maioria das coisas, algumas não que é o que eu tava falando sobre a medicação por exemplo. Eu acho que tem idosa que tem a total condição aí de, de, de tomar as medicações nos horários propostos, mais... eu não vou correr o risco! Algumas não. Vou te dar o exemplo da Yara, a Yara é super lúcida não é?! É uma pessoa que apresenta pra gente com uma lucidez incrível não é?! A vigilância sanitária veio aqui duas vezes, as duas vezes que veio a medicação tava no quarto dela e ela não tinha tomado no horário proposto. Sabe aquela coisa do esqueci?! Oh, esqueci! E aí com o tempo o esquecimento, sabe isso?! Então hoje, por exemplo, talvez eu teria condição de administrar isso, que eu vejo que é uma queixa delas, no inicio foi muito, mas que hoje não vão correr o risco.

*Pesquisador 1: E você associa isso à lucidez?

EP 11: Sim! (silencio) Sim! (silencio) No caso da Yara cê vê que tem aí às vezes tem uma lacuna mesmo assim. Ela brinca mesmo, às vezes acho que eu tô é ficando caduca mesmo (tosse)... Nem acho, eu tenho esquecido tanta coisa assim às vezes, mas, mas acho que sim... acho que sim. É muitas vezes não só a lucidez né?! Tem vez, por exemplo, o caso da Nercina, há pouco tempo a técnica me procurou desesperada, ela não quer tomar uma medicação, aí ela tem que tomar, eu falei: tem? Perguntei pra M... Tem! Ela como técnica, ela tem o dever de ministrar medicação e eu e ela como cidadã né?! Como pessoa autônoma, ela tem direito a recusar qualquer tipo de medicação. Aí é uma discussão que a gente vai ter mesmo assim pra ver como que a gente vai proceder nesses casos né?! E aí não é, e aí é muito doido né?1 Porque cê tá dentro de uma instituição e as vezes cê vai perder os direitos porque você tá dentro de uma instituição. Eu não posso ter o direito de negar uma medicação porque eu moro dentro de uma instituição, será que não?! Eu começo a balançar isso um pouco na equipe. Eu acho que enquanto instituição a gente tem que se precaver né?! Do que pode ter como consequências legais pra gente, então por exemplo, a gente pode fazer um instrumental, que quando ela se recusar, ela vai assinar um documento se responsabilizando, só pra gente se respaldar porque ela tem direito de negar, ela podia descobrir que tá com uma doença grave e não quere fazer o tratamento, isso é um direito dela. Ela tá lúcida? Sim. Então ela responde por ela. Ela não tá interditada né?! Então ela tem direito aí a... a, a responder... a... e muito assim, as de grau I assim por exemplo elas, elas decidem, elas que vão fazer gestão financeira delas, elas que vão organizar a rotina delas né?! Então hoje eu quero ir ali, hoje eu vou encontrar com isso, hoje eu vou, hoje eu quero sair e

comer fora, quero, elas vão, isso por conta delas. Vai pedir auxilio quando é algo aí que extrapola, que elas precisam mesmo de algum é, é aparato aí ??? enfim...

*Pesquisador 1: E Em relação as grau II e grau III?

EP 11: Algumas sim, outras não. A gente tem muita, muitas moradoras aí que já tá num processo de demência mesmo... Isso a gente vai avaliando pouco a pouco sabe?! Então é uma coisa que a gente sente muito assim?! A gente tem percebido que a moradora X, ela já tá tendo dificuldade de, de, de, é de cuidado da higiene pessoal. Então assim, vamo coloca ela na escala?! Pra acompanhamento?! Então, às vezes o cuidador nem vai ajudar no banho como ajuda um grau III, mas vai tá ali presente no momento do banho pra certificar que ela não precisa de ajuda, até risco de queda ou pra ver se, se tá indo da forma como deveria, entendeu?! É... mais auxilio. Como eu tenho moradora também, moradoras que já vai ter um comprometimento cognitivo que não vai aceitar ninguém estar no banho com ela e que a gente vai pensar mesmo em alternativas que sejam menos agressivas assim. Que na casa tinha isso assim: Ah! Vamo pegar, hoje é dia de pegar fulano pra dar banho, aí eu lembro que as meninas chegaram pra mim e falaram assim: a gente pode proceder como a gente procedia? O que? Pegar a fulana pra dar banho. Uai, tcho ver como é que é isso? Eu não esqueço dessa cena... Aí pegava a moradora, ela tomando banho, aí vinha duas cuidadoras, uma técnica e uma cuidadora, elas abriam a porta, trancava e já começava aquela cena de guerra ali dentro né?! Eu só ouvia huafegdiahfg (imita gritos e berros). E eu que isso? Olha o detalhe, essa moradora tem um esquecimento muito grande, ela ficou o dia inteiro atrás de mim. Cê que mandou né?! Dia inteiro! Então isso que foi traumatizante pra ela, ela não esqueceu, ela ficou com aquilo na memória o dia inteiro e durante dias. Eu falei com as meninas: cara, eu acho que, eu acho que o preço é muito alto, eu falei com as meninas assim, né?! Será que tem isso mesmo, de ter que, enfim... Aí a gente começa a pensar em outras saídas, nunca mais pegamos ela a força. Nunca mais, tem pelo mens uns 8 meses que isso não acontece.

*Pesquisador 1: Isso é por causa das rotinas da casa assim?

EP 11: Então como que nós vamos fazer? Então, quando a gente perceber que o banho não tá lá mil maravilhas, vamos incluir a família. Foi o que a gente fez, então o que que a gente faz hoje, quando a gente, quando não tá legal, a Ângela sobrinha dela, pega ela e leva ela num salão pra lavar o cabelo top, no salão, entendeu?! E a gente começa tipo,

como é que tá esse banho aí? E aí essa moradora, ela, ela tem uma questão com a nudez assim, com essa coisa do pudor, que é muito pra ela assim, é uma coisa que não dá, que é meu, meu, seu limite tá ali, então não acho que vale a pena invadir. Como eu já tive uma outra também que, se o pessoal não chegasse pra dar banho ela não tomaria mesmo. Então, a estratégia inicial assim, gente quem foi que falou que todo mundo tem que tomar banho todo dia. Aí começamos a pausar, foi aumentando, só que tinha uma hora que não tinha jeito, que era pegar mesmo. Hoje ela tá tranquila, hoje a gente foi entendendo ali o ritual dela, ver o que que era possível fazer né?! Pra não ser tão invasivo e hoje ela já tá, acho que ela tá permitindo muito por causa do verão, eu acho que o banho até ela tem gostado, no inverno a gente vai tirar a prova dos 9 aí. Mas algumas não têm mesmo assim, não dá pra deixar no time delas. É... outras não. E aí que é isso né?! Relação de equipe, por exemplo, a nossa forma de ver, é uma forma de ficar tentando estratégias o tempo inteiro pra ser menos invasivo e respeitar a privacidade dela. E às vezes o pessoal, da equipe do cuidado, então assim a gente tem que fazer, então vamos lá fazer né?! E o que faz o negócio ser difícil, não é sua capacidade de chegar e pegar a força, isso aí é fácil! Vamos dizer assim. Difícil é cê conseguir fazer isso de forma que seja bom pros dois, então eu escuto queixa todos os dias, por exemplo, do quarto da Cristina e quarto da Tereza. Que as cuidadoras chegam pra mim e falam: Ow, o quarto da Cristina é uma coisa que tem um ano que eu tô trabalhando. Então toda hora que vou lá e ponho uma coisinha lá: Cristina o que que cê acha da gente... Aí eu faço, normalmente eu faço no coletivo, faço uma Assembleia pra todas dizendo: gente! Natal chegando, eu lembro que fiz isso esse natal, vamo, vamo dar uma olhada aí no nosso quarto, ver aí como é que tão as coisas, o que que a gente pode doar, o que que a gente pode retirar, pra ver se... Ela é a primeira a falar: Eu não tenho nada pra doar! É a primeira, e realmente o quarto dela, chega num ponto que fica insalubre, aí que entra o institucional, insalubre não pode! Aí a gente vai ter que começar a pensar mesmo como que a gente vai fazer. Questão da higiene dela também não tava legal... aí a gente tem que pensar estratégias nijfids... falei com as meninas, então foca todo mundo na Cristina agora, vamo tentar limpar o quarto, vamo tenta é... melhorar a higiene dela. Mas eu já tive cuidadora, por exemplo que entrou no quarto dela e pegou todos os produtos de higiene pessoal que ela guardava, que as vezes elas ganham de voluntário e pôs num saco e levou embora. Lindo! Cê vai junto com o saco!... Não dá!...Não dá! Pra ser desse jeito não dá, porque senão a gente já tinha feito, entendeu?! Então a proposta é essa... mas muito assim, e a gente tenta preservar mesmo,

esse, esse lugar de, de, delas... é, é hoje eu falo isso muito com as meninas assim, é, não é fazer por elas, é auxilia-las no que elas interessam assim. A gente tem que criar uma estratégia pra dizer assim: vão lá, check list, fulano, tá tudo ok? Tá tudo suprido, né?! Tá tudo, não. É um movimento também delas ali. Então às vezes, por exemplo, chega, o almoço atrasou, aí vem muito pra esse lugar de cliente. Nossa, mas que absurdo o almoço. Mas porque absurdo, gente? Casa da gente também atrasa, não atrasa? Então é isso assim, aprender a dar conta disso também né?! Dessa coisa do setor emergencial ou então o que que eu posso contribuir para que não atrase, é, é fazer esse processo aí também de, de pertencimento assim aqui. Então as vezes eu recebo doação e eu falo gente, quem que pode me ajudar aqui a descascar 10kg de batata? ... A horta?... Sabe?! Quando tem férias e muda a escala da, da cozinha, eu às vezes peço uma moradora pra ajudar. Quem pode me ajudar a lavar prato? Porque a auxiliar vai embora mais cedo e ai fica muito pesado pra cozinha... É, a gente passou por uma situação agora, por exemplo, da Tereza que uma cuidadora ajudava ela com a questão financeira e a gente mudou um pouco isso... e ela tá passando aperto! E aí, ela demanda pra casa assim, é... como se a gente tivesse mesmo que suprir tudo pra ela, e aí eu falei com as meninas principalmente porque é um quadro de depressão, eu acho que o trabalho não é esse, o trabalho é exatamente a gente... deixar ela fazer um movimento aí pra achar saídas dela. Vai sofrer mesmo gente, a vida é isso pra todo mundo, mesmo ela sendo idosa assim, como que cê vai sair disso. Então às vezes ela me procura, ah, porque fulano... é e aí, como que cê vai fazer? Entendeu?! De, de ficar aqui, vendo se vai cair, mas de deixar ela andar mesmo porque senão fica muito. A tendência de entrar numa ILPI, é o que eu falo as vezes com as meninas, por isso eu acho que ILPI pra grau I as vezes não é, não deveria. A tendência, é ela perder a autonomia e a independência cada vez mais! Isso é fato! (enfática) Isso é fato! Tem relatos aqui que me surpreendem, de morador que entrou aqui com controle de esfíncter, com controle urinário e que usa fralda hoje porque a rotina não permite que ela vá ao banheiro a hora que ela quer. Isso é serio! Entendeu?! O que tô te falando? Ela é cadeirante! Então pra ir ao banheiro ela precisa de alguém! Isso é cruel docê pensar! Que ocê vai começar a usar fralda porque cê não pode ir ao banheiro a hora que cê quer! Cruel! Quando eu cheguei isso me matava! Mas sinceramente falando, vai ter dia que não vai ter jeito (diminui o tom de voz)... mesmo... entendeu?! Então, a rotina vai tá aquela loucura, horário de banho tal, se ela falar: quero ir no banheiro agora! Não vai ter alguém pra fazer a transferência dela pro banheiro. Então, a tendência de uma ILPI é praticidade, é isso, não tem jeito né?! São 28

moradoras, mais ou menos 15 vai depender aí do auxilio das cuidadoras, hoje eu tô com duas cuidadoras no plantão do dia. (silencio) Entendeu?! Então, às vezes vai entrar nesse momento aí do, vai entrar no automático. E olho que luto muito pra isso assim oh, pelo, pelo meu posicionamento, pela minha profissão, então assim, a tendência é eu lutar por isso o tempo inteiro. Então por exemplo, a Terezinha, que é uma moradora que ela tá num momento aí de: eu quero ficar na cama. A gente discutiu muito sobre isso né?! Se a Terezinha deveria ficar ou não, se a gente devia intervir de uma forma, como que a gente ia intervir. Pra dizer pra equipe do cuidado que a Terezinha ficaria no quarto e elas deveriam servi-la no quarto, foi um trabalho aqui de tempos, pra elas entenderem, pra eu sentar com elas e falar com elas, por que não? Ah porque, tratamento vip, tratamento vip, tratamento exclusivo?! Tratamento vip e exclusivo porque ela demanda isso e as outras não demandam. As outras tem condição de ir pro refeitório almoçar ou as outras desejam ir pro refeitório almoçar e ela não deseja. Entende?! Então o trabalho assim que cê vai ter pra colocar ela fazendo uma transferência na cadeira pra levar lá, você vai gastar menos levando o prato e a gente ainda respeita a vontade dela, mas foi um trabalho que demorou. Pra elas entenderem que é possível fazer isso aqui sim né?! É possível um dia, alguém não querer determinada comida e comer outra comida, é possível sim. Então enquanto isso não for um problema pra gente, a gente vai fazer. Vai ser problema no dia que várias... tiver desejos diferentes. Aí eu acho que homogeneíza mesmo, não tem jeito. Coletividade não dá pra pensar em cada um o tempo inteiro, mas dá pra gente fazer exceções sim, tem que ter...
