# Supplementary material for: Care for older adults with disabilities in Long Term Care Facility
Source: Rev Bras Enferm. 2023 Dec 8;76(Suppl 2):e20220767. doi: 10.1590/0034-7167-2022-0767 (PMC10704689; doi:10.1590/0034-7167-2022-0767)
Supplement: 0034-7167-reben-76-s2-e20220767-suppl09 [file 0034-7167-reben-76-s2-e20220767-suppl09.pdf]

EP 12

1) Pesquisador 1: **Como é, pra você, trabalhar em uma ILPI?**

EP 12: Olha, foi uma experiência tardia, mais muito proveitosa, quando eu cheguei aqui, há três anos atrás, eu falei pra irmã assim: que era, eu vinha de uma outra instituição, que chamava Aconchego, ali da Cidade Jardim, tinha sido muito bom, mais foi uma carga horária muito pesada e trabalho muito pesado. Então quando eu cheguei aqui, pra mim foi uma realidade muito diferente e gratificante, porque aqui, as irmãs me deram a oportunidade de fazer o que era o meu objetivo anterior e eu ainda disse pra ela, que se eu tivesse condições de fazer, de prestar esse serviço voluntariamente, era o que eu gostaria de fazer, mas eu não podia ser hipócrita, que eu precisava do salário, então tava tendo o privilégio de unir as duas coisas, era um trabalho que eu gosto muito de fazer, estou tendo ainda essa oportunidade de fazer e recebendo o salario por isso. Então tá, é muito bom, é muito prazeroso, cada dia a gente descobre assim um universo diferente sabe, muda muito a mente da gente, depois que cê passa a trabalhar com idoso, você descobre que não é um velhinho que não presta pra mais nada, não é uma pessoa que viveu, tem uma bagagem enorme na vida, que traz pra gente essa oportunidades, eles tem limitações, aí aqui entra nossa oportunidade de prestar esse serviço pra eles, você vai ser os olhos de quem não tá vendo, a perna de quem não anda, né?! E o ouvido de quem não tá ouvindo, então é muito prazeroso, muito prazeroso e eu tive, tive essa felicidade de chegar aqui nessa, nessa visão de poder servir as pessoas que era, tinham as limitações e até hoje estou fazendo com muita, muita alegria de fazer, porque realmente é muito prazeroso, muito proveitoso, você saber assim, que as pessoas dependem de você, mas são muito gratas pelo que você fazem por elas, quando elas já não podem mais, muito bom.

*Pesquisador 1: Bonito.

2) Pesquisador 1: **Me fale um pouco sobre seu relacionamento com os idosos que residem aqui.**

EP 12: Bem, meu relacionamento com as idosas que moram aqui, é um pouco assim, é, acho que assim um tratamento é excessivo, porque se eu me envolvo demais com elas e elas ficam muito, uma dependência muito grande por mim, elas ficam o tempo todo assim, cê sai elas já querem saber a hora que cê vai voltar, aí põe na cabeça delas que você sabe fazer as coisas pra elas e começam criar até uma, uma preferência delas por, porque assim,

eu paparico demais, na verdade é essa, minhas colegas, algumas até questionam muito isso, mas eu falo desde que eu não esteje atrapalhando a rotina dos outros colegas e dos outros profissionais da casa o que que tem você paparicar, o que que tem você da um, porque eu paparico mesmo, eu faço todas as vontades delas na medida do possível, quando eu não posso fazer, eu morro por isso, porque assim, eu vejo pra mim, assim são as minhas mães, são as minhas irmãs, minhas filhas, tem umas aqui que elas chamam a gente até de mãe, tamanho o afeto que cria com a gente, então assim as vezes eu acho que eu ultrapasso até o meu trabalho de cuidadora, sabe?! Eu sei que eu excedo muito nesses cuidados meus que acaba criando esse vinculo de afeto muito grande com elas, mas elas são muito agradecida por todo atenção que eu dou a elas, elas são responsivas, elas respondem, e eu acho até muito interessante quando tem alguma colega que chega que eu vou treinar, eu lembro elas, olha aqui nunca é, menospreze a idosa, por mais que cê ache assim que ela não tá ouvindo, que ela não tá de entendendo, que ela não tem consciência, você não sabe até onde que vai o nível da consciência. Então respeite a idosa, quando você vai tocar nela, pede ela licença, fala com ela quem é você, se apresenta, eu até chamo muita atenção das minhas colegas, fala pra ela, quem é você, o que que você vai fazer, que elas gostam disso sabe, e falo muito assim, nunca menospreze, que cê não tem ideia até onde que vai a consciência das pessoas e elas respondem, as vezes você acha assim, a pessoa num tá na fase assim que num responde a nada, pode ser considerar até uma fase vegetativa, de repente ela abre o olho assim pra você quando ouve sua voz e demonstra, dá um sorriso pra você assim pelo tom da sua voz, aí cê fala assim sabe que a pessoa tá te entendendo, sabe que é você que tá cuidando sabe?! Então é muito, muito agradável, é claro quando, as vezes as idosas que são independentes eu tenho dificuldades sim, inclusive eu converso muito isso com a equipe técnica é a respeito disso, se você for ver com elas assim eu fico o tempo todo atrás delas peço informação quando não tô conseguindo entender atender ou uma idosa, quando eu percebo qualquer comportamento nelas e falo assim me ajude, faz uma intervenção pra mim, mostra, fala pra gente como que vai lidar com essa situação ou outra e, porque as idosas que são independentes muitas vezes assim me faz não sei se é todas as pessoas me faz pensar assim que tá fazendo aqui, porque quantas pessoas que precisam verdadeiramente e assim eu as vezes saio, peco até nesse ponto eu fico achando que elas não precisam dos cuidados que eu posso outra colega minha da, porque elas se tornam umas pessoas assim muito é grosseiras com as outras, é, moradoras, porque as outras moradoras babam, as outras moradas usam flalda, num andam, são dependentes, a comida e toda higienização delas nos que fazemos. Então as

vezes se sabem por elas, ainda não estarem nesse grau III, elas serem privilegiadas, torna as vezes assim uma postura muito arrogante com as outras idosas e eu fico muito incomodada com isso tenho muita dificuldade de lidar com essas idosas independentes, que são pessoas assim que andam e pra atrás das coisas que precisam que tem as coisas, não tem um pingo de respeito sabe?! As outras moradoras que estão aqui, então eu não consigo lidar entre aspas numa boa, eu como cuidadora, como funcionaria se elas precisarem de mim eu tento prestar cuidados a elas que é pra isso que eu estou aqui, minha profissão é essa, cuidadora, então se precisar dos meus cuidados estou, não faço isso com boa vontade pra elas, não adianta falar pra você que faço que eu não faço, faço porque é obrigação minha como funcionaria da casa, mas as outras que são totalmente dependentes e semi independentes eu acho maravilhoso, maravilhoso trabalhar com elas e é assim eu trabalho, eu falo assim o meu trabalho aqui ele é diferente, tem um diferencial assim que eu num consigo é por mais que seja por exemplo, nos tivemos nos banhos que é nossa atividade mais pesada, eu dei dez banhos, mas eu não consigo falar pra você assim que eu estou cansada, se eu tiver que da banho nas vinte oito eu vou da, porque assim eu sinto assim um privilégio muito grande quando estou fazendo esse trabalho eu sinto assim, aquele retorno tão grande, eu vou pra casa leve, livre e solta e feliz da vida. Então o meu trabalho aqui é muito bom, pra mim e pra elas que eu cuido também, eu acho que assim.

3) Pesquisador 1: **Qual a sua percepção sobre a relação dos idosos institucionalizados com seus familiares e amigos?**

EP 12: Qual que é a percepção?

*Pesquisador 1: É.

EP 12: É muito interessante, elas assim, elas participam da vida da gente de uma tal forma, por exemplo, se sabem que eu sou casada, pergunta pelo meu marido, pelo meu filho, as vezes um filho meu vem aqui, aí eu faço questão levo pra apresentar, depois elas perguntam de novo, elas sabem que, que, e gostam disso, dessa de quando a gente traz noticia, quando a gente leva daqui também delas noticia pra eles, é muito, elas percepção muito boa.

*Pesquisador 1: E cê acha que elas recebem visitas, recebem amigos, familiares aqui na casa ou não?

EP12: Poucas, ou aqui cê pode assim, é enumerar, das vinte e oito idosas, eu te digo assim que menos de dez por cento receba visita de familiares principalmente. Talvez assim, das vinte oito eu tiraria umas seis, cinco no máximo que recebam visita de familiares e de amigos, muitas delas as visitas são pessoas anônimas que vão se tornando amigas e a partir do momento que começa a prestar trabalho voluntariado, né?! Principalmente quando vem rezar aí visitam assim ou a capela ou a instituição mais se tornam amigas, mais assim, familiares e amigos a maioria delas que estão aqui não tem vínculo, vínculo com parentes. São mulheres a maioria solteiras, que não casaram, que não tiveram filhos e muitas delas tiveram a opção até de nunca ter tido nem um contato físico com um homem, nunca tiveram essa experiência e não tem mais os pais que elas já são velhas, muito velhas e não tem irmãos. E as algumas que a gente fala assim que sabe, elas contam pra gente quando tem irmãos ou coisa, são a minoria, minoria muito poucas mesmo, agora essas poucas que tem a relação delas com a família é bem bacana, sabe?! Bem bacana.

4) Pesquisador 1: **Você considera que os idosos dessa ILPI têm condições de tomar decisões sobre as coisas que precisam fazer em seu dia-a-dia? Por quê?**

EP 12: Sim, a minoria, essas que são independentes sim, que nem é perfil pra estar em uma ILPI, né?! E isso assim, essas têm, mais a maioria.

*Pesquisador 1: E as outras?

EP 12: Não, nenhuma, elas não tem autonomia nenhuma, elas são totalmente assim, desprovida de, de conhecimentos e são leigas, muitas são analfabetas, outras são é tem uma, uma deficiência mental, já tem um a parte do intelectual delas tão bem comprometidas, elas não tem gerencia, não sabe, não dão conta de gerenciar a parte financeira, não tem controle sobre isso, não conhecem mais valor de dinheiro, não conhece mais ruas, tem até o caso de uma dessas idosas que, eu não sei se eu tô te respondendo direito, porque assim, quando eu te falo assim, da dessa resposta que eu tô te dando pras idosas que são dependentes.

*Pesquisador 1: Então vou te fazer uma pergunta: você acha que a lucidez que define, que orienta a tomada de decisão?

EP 12: Sim, sim, pessoa lucida ela é capaz de decidir.

*Pesquisador 1: E de coisas simples, como comer, tomar banho?

EP 12: Não, não precisa ser lucido pra poder ter, a questão de hábito de costume, aí a gente vê assim, temos até, assim aí cê lembra de cada casim assim, pra você exemplificar, por exemplo nos temos idosas aqui que, elas até lembram “ah quando fazia, minha mãe fazia”, “você faz igual minha mãe”, desde pequena elas lembram da época da infância, que aprendeu com a mãe, então elas tem aquele cuidado, a higiene de cuidado, de tal coisa que aprendeu na vida e mantem. Tem umas aqui, idosa que é a mais nova de todas elas, apesar de ser a mais dependente, ela não tem mais, ela não sabe nem a resposta pra você de tamanha é a parte do celebro dela, já não reponde e no entanto assim, é uma pessoa, uma classe, uma elegância, pra comer, mastigação dela é muito lenta devagarzinho, no tempo dela, ela senta, ela se ajeita nas roupas, ela preocupa, ela quando ela tá suja, que ela sabe assim, que ela num fica incomodada. Não tem nada a ver com a lucidez, que ela não é lucida, elas me, tem várias, essa em especial percebe que não tem a ver com a lucidez, só que não sabe dar resposta pra uma coisa, não sabe, fica, é dois mundos, eu não sei te responder claramente assim, mais claro do que isso, a gente percebe no dia a dia essas dificuldades. Agora já tem umas que são lúcida aqui, que são pirracentas, por exemplo a Eugênia, não gosta de tomar banho, não gosta de trocar de roupa, num gosta de, que tem mania de acumular, são acumulativas querem acumular coisas sujas, e são lucidas, então não tem nada a ver, né?! É bem complexo.
