# Supplementary material for: Care for older adults with disabilities in Long Term Care Facility
Source: Rev Bras Enferm. 2023 Dec 8;76(Suppl 2):e20220767. doi: 10.1590/0034-7167-2022-0767 (PMC10704689; doi:10.1590/0034-7167-2022-0767)
Supplement: 0034-7167-reben-76-s2-e20220767-suppl11 [file 0034-7167-reben-76-s2-e20220767-suppl11.pdf]

EP 1

1) Pesquisador 2: **Como é, pra você, trabalhar em uma ILPI?**

EP 1: Pra mim é bom, eu gosto do que eu faço. É um serviço assim, que cê tem que ter muito cuidado. Mas eu gosto do que eu faço.

*Pesquisador 1: E porque que tem que ter muito cuidado?

EP 1: Porque mexer com idoso é muito cuidadoso, porque a pele é sensível, cada um é diferente um do outro. Né?! Cê tem que, principalmente o cadeirante, você tem que ter cuidado de manusear ele na cadeira, tirá, colocá. Qualquer esbarrão dá hematoma. Então tem que ter muito cuidado.

2) Pesquisador 2: **Me fale um pouco sobre seu relacionamento com os idosos que residem aqui.**

EP 1: Graças a Deus tranquilo. Nunca tive problema com nenhum dos idosos. Me dou super bem com todos eles. Desde que eu entrei, é tranquilo. Nunca tive uma reclamação, nunca machuquei nenhum deles. É tranquilo.

3) Pesquisador 2: **Qual a sua percepção sobre a relação dos idosos institucionalizados com seus familiares e amigos?**

EP 1: Sentem muito abandonado. A maioria, tem uns que tem família que vem ver, que são uns quatro mais ou menos. Mas a maioria se sente abandonado, pelos familiares, pelos amigos. Eles reclamam muito, questiona muito isso. São choroso nas data festiva, porque ficam aqui não tem visita, não vai pra casa de parente.

*Pesquisador 1: A maioria delas não casou nem teve filho né, pela história de vida delas. Então assim, isso é uma coisa que afasta elas, um pouco, dessa vida social mesmo, de ver família, as vezes de família... Porque uma coisa é visitar a mãe, a outra é visitar a tia.

EP 1: Com certeza (enfática), isso daí influencia muito. Porque a maioria não tem filhos, as que têm filhos já faleceram. Tem umas, algumas tem filhos, mas já são falecido. Mas isso também ajuda muito por ela se sentir sozinha. Porque já tão de idade, as irmãs também, muita das vezes, seriam até mais velhas e sem condições de locomoção também pra poder visitá-las.

*Pesquisador 1: Hummm. Entendi. Eh, acaba que com isso elas ficam muito sozinhas...

EP 1: Fica muito sozinha mesmo... Nem é por maldade, é por condições mesmo.

*Pesquisador 1: Entendi.

4) Pesquisador 1: **Você considera que os idosos dessa ILPI têm condições de tomar decisões sobre as coisas que precisam fazer em seu dia-a-dia? Por quê?**

EP 1: A minoria.

*Pesquisador 1: Que minoria seria essa?

EP 1: Não vou dizer minoria, a maioria tem condição de decidir. As que andam mesmo, ah a maioria das que andam, que são independente. Tem condição de dicidir, de sair, deee. Querer passear, querer ir lá fora, elas tem condições pra isso. Porque aaas outras não, as outras depende da gente, não tem tanta lucidez assim. Tem algumas que tem problema de memória, então não teria condições.

*Pesquisador 1: E cê acha então, que essa condição de poder fazer as coisas sozinhas e as que não podem fazer as coisas sozinhas, é o que define assim se elas, se elas decidem ou não sobre as coisas delas? Só o fato delas andarem?

EP 1: Não, o fato da lucidez delas.

*Pesquisador 1: Da lucidez?!

EP 1: Da lucidez. De saber ir e vir. Saber o endereço de como voltar. De pegar um taxi, de dar um troco. Né?! De pagar um Uber. Deee... desse sentido. Não só por andar, mas da lucidez.

*Pesquisador 1: E tem, eee muitas delas né que assim, as vezes são cadeirantes, mas que são lucidas ou nem precisa ser cadeirante, mas que consegue decidir sobre a própria vida. Cê acha que é dado liberdade pra elas?

EP 1: Ahhhh, eu... ahhh eu, no meu modo de ver, eu acho que não. (Silêncio) Pelo que eu vejo não. Porque tem uma mesmo, a Fia, vive falando que queria sair daqui, ela não sai. Eles não tira, a família não tira, então vou tirar base por ela. Que pede pra embora, que quer e ela não...

*Pesquisador 2: E ela é lúcida?

EP 1: Ela é lucida, ela é só é cega, mas ela é lucida. E ela pede pra ir embora e não vai.

*Pesquisador 1: Entendi. As vezes a decisão não depende só delas né?!

EP 1: Não, eu ah..., eu pra mim, o que eu vejo, é a decisão depende da família. A família tem que decidir e a família decide por deixá-la aqui.

*Pesquisador 1: Entendi.

OBS: (Mas se a grande maioria não tem família. Negação – não acham que a forma como elas profissionais e instituição tratam, interfere na autonomia. Só a família.)
