# Supplementary material for: Care for older adults with disabilities in Long Term Care Facility
Source: Rev Bras Enferm. 2023 Dec 8;76(Suppl 2):e20220767. doi: 10.1590/0034-7167-2022-0767 (PMC10704689; doi:10.1590/0034-7167-2022-0767)
Supplement: 0034-7167-reben-76-s2-e20220767-suppl12 [file 0034-7167-reben-76-s2-e20220767-suppl12.pdf]

EP 2

1) Pesquisador 1: **Como é, pra você, trabalhar em uma ILPI?**

EP 2: Trabalhar... oh quando eu formei o curso, vou explicar do meu jeito se não for isso depois. Quando eu formei o curso, eu não pensava em trabalhar não, eu cai... ganhei o curso e comecei a fazer. Aí quando eu formei ate eu formar, eu achei que pra mim, aí eu já gostei da área e falei assim: não, então eu quero trabalhar, eu falava asilo né, quero trabalhar num asilo. Aí até comecei a trabalhar na casa de um pessoal particular e saiu aqui, eu vim. Particularmente, eu gosto muito daqui, tô gostando, ni outros lugares eu nunca fui, não sei, mas eu gosto muito daqui, pra mim assim, tá sendo muito bom, entendeu? Eu assim, tem seus lados ruins, mas ta sendo bom pra mim, porque eu terminei o curso, vim pra cá e gostei bastante.

*Pesquisador 1: E assim, antes você trabalhava na casa de uma pessoa, né?

EP 2: Não, eu formei em 2015, quando foi inicio de 2016 eu comecei a ficar na casa de um casal de idosos, só final de semana, entrava no sábado e saia na segunda. Aí me chamaram aqui, aí eu vim pra cá.

2) Pesquisador 1: **Me fale um pouco sobre seu relacionamento com os idosos que residem aqui.**

EP 2: Ah, eu acho que é tranquilo, apesar que tem hora que a gente tem que dá uns, assim, não é ser grossa, mas a gente tem que ser mais né, tem uns também que são mais tranquilos, tem outros que tem hora que já, se deixar mete a encrenca. Por exemplo, a Eugenia, Eugenia tem momentos que ela é tranquila, tem momentos se deixar ela vem pra cima da gente, entendeu? Então assim, eu acho tranquilo, em termos assim, de conflito, brigar nunca tive problema assim não. Entendeu?

*Pesquisador 1: Você fala que as vezes tem que ser mais brava com alguns, mas pelo comportamento deles?

EP 2: É, porque que nem a Eugenia, tem hora que a gente chega perto da Eugenia e ela é muito assim, ela gosta muito de ficar ali na beirada do portão, aí você vai na paciência: "Eugenia vão bora!" e ela: "Não." Aí tem hora que ou vai num arrancão mesmo: "Vão bora" ou se não você tem que chegar e falar bravo mesmo: "Eugenia vão sair daí' porque se não ela não vem. Entendeu? No mais é tranquilo.

3) Pesquisador 1: **Qual a sua percepção sobre a relação dos idosos institucionalizados com seus familiares e amigos?**

EP 2: Ah, tem uns que são presentes, aparentemente presente, parece que é tranquilo. Tem outros que eu nunca vi parente, aqui não. Entendeu? Nem sei se tem, já ouvi falar que tem uns que tem, mas nunca vi. Entendeu? Então assim, tem uns que são muito presentes, que nem daquela senhora da Fia, entendeu? O pessoal dele é bem presente.

*Pesquisador 1: E você acha que elas convivem bem com isso, assim?

EP 2: As idosas?

*Pesquisador 1: É.

EP 2: Vive, com o pessoal delas?

*Pesquisador 2: Não, você não acha que elas não sentem falta, de ter mais contato?

EP 2: Eu acho que sente.

*Pesquisador 2: Elas comentam, falam alguma coisa?

EP 2: Não, não são muito de comentar não, mas da pra gente ver que sente, entendeu? Que nem tem uma mesma que ficou aí final de ano, dezembro e janeiro, no final de dezembro, Natal e Ano Novo, na expectativa da família dela aparecer, falou que, falou que eles iam levar e não apareceu, entendeu? Então sentem, entendeu?

4) Pesquisador 1: **Você considera que os idosos dessa ILPI têm condições de tomar decisões sobre as coisas que precisam fazer em seu dia-a-dia? Por quê?**

EP 2: Digamos assim, que são lúcidos sim, né, mais a maioria não, a maioria eu acho que não.

*Pesquisador 1: E você que a lucidez é que define isso?

EP 2: Ah, eu acho que sim, porque por exemplo, a Fia mesmo, a Fia quem vê a Fia fala que ela é lucida, mas a Fia é muito desorientada, entendeu? Então assim, pra mim ela fala que quer ir embora, mas na casa dela eu sei que não tem ninguém pra cuidar dela, quando trouxe ela pra cá, numa situação meia vamos dizer assim, abandonada né, não sei assim como que era certo não, mas pelo menos eu já ouvi. Então não tem como ela falar que quer ir embora, como que ela vai tomar uma decisão que quer ir embora se não tem ninguém pra cuidar dela.

*Pesquisador 1: Então você acha que as vezes, essa decisão passa pela família, também?

EP 2: Eu acho que passa, eu acho que sim né, porque pra ela ir ficar sozinha em casa, também como que ela vai? Entendeu? Então, eu acho que por mais que ela tenha uma lucidez, mas assim, pra ela falar assim: “eu quero ir embora” que nem você virar e falar: “pode ir”, eu acho que pra ela num é viável, entendeu? Porque né, pela situação que eu já ouvi falar de muitos aqui, por mais que cê tenha uma lucidez, perceba que tem uma lucidez, mas pra resolver, “eu vou”, não tem condições de falar “tô indo embora” assim não. Então eu acho que tem que sentar, rever, conversar com a família, entendeu? Então eu acho que nem assim, lucido pra mim mesmo, que tem condição de decidir as coisas, é que nem a Gilberta, a Graça que são pessoas independentes, que saem, vai né.

*Pesquisador 1: E decidir sobre as coisas aqui da casa? Coisas as vezes que elas querem fazer, você acha que existe essa abertura?

EP 2: Na minha opinião, não.

*Pesquisador 1: Porque não?

EP 2: Ah, porque eu não vejo elas fazendo isso, entendeu? Assim, se virar e falar assim: “ah eu quero fazer isso”, eu acho que eu nunca vi essa oportunidade pra elas, terem essa oportunidade de fazerem isso.

*Pesquisador 1: Você acha que elas fazem mais o que todo mundo faz?

EP 2: É.
