# Supplementary material for: Care for older adults with disabilities in Long Term Care Facility
Source: Rev Bras Enferm. 2023 Dec 8;76(Suppl 2):e20220767. doi: 10.1590/0034-7167-2022-0767 (PMC10704689; doi:10.1590/0034-7167-2022-0767)
Supplement: 0034-7167-reben-76-s2-e20220767-suppl13 [file 0034-7167-reben-76-s2-e20220767-suppl13.pdf]

EP 3

1) Pesquisador 2: **Como é, pra você, trabalhar em uma ILPI?**

EP 3: (Pausa) olha, é um trabalho que eu gosto, de trabalhar com idoso. Eh.. trabalhei na fundação com criança, foram duas, dois lados bem opostos, né?! Que é o idoso e a criança, e é um trabalho que eu gosto, assim não tem, não sei te falar assim, mais é uma coisa que eu gosto mesmo, tipo, de trabalhar com idoso.

*Pesquisador 1: Você acha que é uma vocação?

EP 3: Não, acho que é gostar mesmo. Porque, eu gosto de outras áreas também, então, não acho que seja vocação não, eu acho que é porque eu gosto mesmo.

Pesquisador 2: E aqui foi sua primeira experiência, com idoso?

EP 3: Sim.

2) Pesquisador 2: **Me fale um pouco sobre seu relacionamento com os idosos que residem aqui.**

EP 3: São 7 anos aí, vamos colocar, então é bom, tipo, é ... é claro que eu entrei, era um outro perfil de idoso, é... de quando eu entrei pra hoje, tem poucos né?! Então hoje, uns saíram, outros faleceram, mas o relacionamento é bom, eu não tenho, eu gosto de conversar com elas, de sentar com ela, sei que elas gostam também, que elas me procuram. A gente conversa, brinco com elas, então assim, é bom. Não tenho ... muito assim, o que falar.

3) Pesquisador 1: **Qual a sua percepção sobre a relação dos idosos institucionalizados com seus familiares e amigos?**

EP 3: Então, a maioria que tá aqui, não tem família né?! Alguns tem, é ... com ... uns com vínculos muito fragilizados, outros não, se cê for pegar hoje a gente tem 28 idosas, se cê for pegar, as que tem família ... é ... na verdade assim a família que não é presente hoje, cê pode reparar que é por algum motivo financeiro, de doença que não tem condições de cuidar do idoso. Mas por exemplo, tem a ... uma idosa aqui, é, ela, na verdade tem dificuldade de aceitar morar numa instituição, mas o filho dela é extremamente presente, mas ele não tem condições financeiras de cuidar sozinho, ela tem outros filhos, mas os outros filhos também não têm condições, que são todos idosos também e ... mas a maioria aqui não tem família assim. Não teve aquela briga, aquele,

aquele, aquele vínculo assim, elas são sem famílias , que tem tipo, ou que perderam família.

Pesquisador 1: Normalmente elas não têm filhos, né?

EP 3: A maioria não tem, a maioria não tem filhos, é ... que tem filho aqui, são duas ... é duas, que tem filhos vivos, né?! Que tem filhos de outras, que já faleceram, então a gente tem uma que tem filho é ... que é esse né?! Que não tem condições de cuidar, de levar a mãe pra ficar. A gente tem uma outra também, que tem um filho, mas ele tem, ele é doente, ele tem uma esclerose múltipla que, a esposa dele, tipo, não tem condições de cuidar da sogra e dele, então, é por isso que ela veio pra cá. É, mas a maioria é essa, solteira, é que não casou, que não teve filho, ou então as que tiveram filho que já faleceram, que não, a gente tem uma que, ela teve dois filhos, eles faleceram, ela perdeu o contato com os netos. Então assim, não tem vinculo assim não, agora, é ... elas têm muitos amigos, assim, os amigos são bastante presentes, quando a gente liga, pra chamar pro Chá e Prosa, aí vem, amigo, tem é ... sobrinho também, né?! Na verdade, assim, a maioria tem sobrinho, os sobrinhos vêm, a gente sabe que sobrinho não tem a obrigação, mas ele vem, eles cumprem esse papel né, de família. A casa Santa Zita, na verdade, é um pouco diferente né, das outras assim, se você for procurar um de vinculo rompido, de confusão, de possibilidade de reinserção, não tem, porque elas são sozinhas na verdade, aí você não acha.

*Pesquisador 1: Pela história de vida delas?

EP 3: É, pela história de vida delas.

*Pesquisador 1: De não terem casado, não terem filho.

EP 3: É, ou então as que casaram e filho morreu, marido morreu, é... não teve contato né?! Com neto, com sobrinho, a gente tem uma aqui, que ela tem dois netos, mas assim, os netos passam por dificuldade, não tem condições de cuidar da vó. Então assim, é cada história assim, é diferente assim.

*Pesquisador 1: E como cê acha que isso interfere, assim? Na relação delas, não só com a família, mas com o mundo, cê acha que elas participam, você acha que isso isola elas? Você acha que isso faz com que elas queiram mais atenção?

EP 3: Não.

*Pesquisador 1: Você acha que elas sofrem isso, ou elas vivem bem com isso?

EP 3: Tem umas que aceitam bem, que reconhecem que foi uma escolha mesmo, né?! Que a vida é uma escolha, que foi uma escolha, que não casaram, não tiveram filho, que isso é uma escolha e estão certa da escolha. É tem, a gente tem caso aqui da idosa que não casou, não teve filho, mas arrepende amargamente de não ter casado, hoje vive com uma frustração muito grande, essa que principalmente vive com essa frustração, ela requer uma atenção, tipo, ela é muito depressiva, ela, ela exige uma atenção assim, que por mais que cê dê atenção, ela quer mais, mais e mais e mais. É ... tem a ... as outras que tão aqui, sabem que precisa estar aqui, porque a família não tem condições, mas num, não são revoltadas, assim, de tá aqui, entendeu? Talvez queriam ficar com a família, mas reconhecem que não tem condições. Aí difícil falar assim, não sei, não acho que interfere assim não, não acho que elas ficam é ... muito tristes assim, tipo, a ponto de entrar em uma depressão, por estar aqui não, num acho que isso acontece não, eu acho sim, que ficam triste talvez, por saber que não tem condições de tá lá pra ajudar, o filho, um neto, naquele momento, mas não a ponto de ficar muito deprimida, de não querer socializar com as outras, de não querer participar de alguma coisa, acho que não.

Pesquisador 2: Cê falou que a instituição liga pros amigos e tem um Chá e Prosa, né?!

EP 3: A gente tem um evento, que ele é três vezes ao ano, e a gente ... é chama Chá e Prosa que é junto com as Meninas do Lar e os familiares ou amigos, porque né?! Não são todas que tem familiar, então aí assim, a gente liga pra convidar, pra vim ficar, passar esse momento com a idosa e assim, é sempre bem recebido, todos vem, sabe?! Os que não vem é que não podem vir mesmo, mas aí a família vem, os que não tem família, vem amigo, traz amigo. Então assim, elas, eu acho que na verdade elas acostumaram, quando eu entrei aqui, é, como eu falei antes, não era esse perfil, era um outro perfil, é não tinha o publico da prefeitura aqui, apesar de ter o convênio, mas não tinha, era mais é por demanda espontânea, que por demanda espontânea aqui era aquela coisa, tipo, eu não casei, não tive filho, quero morar aí, então era uma coisa bem, bem resolvida delas assim. Agora que a gente vem com o público da prefeitura que é um público diferente, que tem família ou perdeu família né, que vem, mas assim, é ... eu acho que de tudo assim, não tem ... eu acho que não tem assim, elas não sintam falta, não sentem tipo: “no que tristeza, tô aqui”, eu acho que não sabe?! Ou se sentem, de todos os atendimentos que eu já fiz nenhuma me passou isso, tipo: “nossa não queria tá aqui”, só essa que é depressiva, que não casou que ela fala, tipo: “eu queria ter casado,

mas não casei”, é, mas enfim foi escolha dela, ai ela é a única aqui que fala isso, tipo é: “se eu tivesse filho, meu filho taria me olhando” e “não sei o que”, a única assim, as outras não, tipo, são bem, eu acho que aceitaram assim, a escolha assim e os amigos são bastante presente, a casa é muito visitada também né?! Então tipo, é elas não se sentem tão sozinhas por causa disso, é central que vem muita gente pra cá, vem muitos voluntários pra cá. Então, tem uns outros voluntários que já conhecem muito tempo muitas idosas aí, que leva pra casa, né?! Tipo tem uma voluntaria aí, que ficou superamiga das idosas, que faz questão de leva algumas idosas na casa dela, oferece um café lá, então assim, eu não acho que elas sentem assim, se sentem nunca, nunca, manifestaram assim, essa tristeza delas, assim não, eu acho que elas aceitaram assim, de boa.

4) Pesquisador 1: **Você considera que os idosos dessa ILPI têm condições de tomar decisões sobre as coisas que precisam fazer em seu dia-a-dia? Por quê?**

EP 3: Alguns, outras não.

*Pesquisador 1: Por que?

EP 3: É pela aquela, aquela avaliação mesmo, do grau de dependência, vai vendo que perde cognitivo né?! A fala/falha vai apresenta alguma demência. Eh, vai ficando confuso, não consegue decidir, mas aí é claramente, tipo assim, qua... quando você convive com eles, você consegue perceber. O idoso que tá, que tá confuso, aquele que tá iniciando uma demência. Aquele que é lucido, que sabe o que quer. Então assim, não são todos não, que consegue fazer isso não. Só os mais independentes.

*Pesquisador 1: Então você acha que é a lucidez é que define se eles conseguem tomar decisões ou não?

EP 3: Não! Talvez não. Por exemplo, nem sei. Vamos supor, vamos supor a gente vai fazer uma atividade. Eh. A gente tem uma idosa que ela, eh, que o cognitivo dela é muito baixo e cê pergunta pra ela: cê quer participar? Aí ela fala: não, hoje eu não quero! A gente sabe que ela não quer, que ela manifestou a vontade de não querer, então a gente não obriga ela a participar. Agora se for tomar alguma, por exemplo se ela for tomar alguma decisão da vida civil dela, ela não consegue. Então assim, depende assim. Alguma coisa aqui dentro, elas conseguem manifestar, outras não. Aí tipo, por exemplo, a Bel... que não manifesta nada, não verbaliza nada, então assim, ela não, se ocê colocar

ela numa atividade, tirar da atividade, pra ela vai ser indiferente. Assim, na verdade eu não sei se ela sente alguma coisa ou se ela percebe alguma coisa, mas ela não manifesta nada. Então assim, não sei, acho que depende muito da idosa assim.

*Pesquisador 1: E cê acha que pra essas que são lúcidas, elas, eh, porque, ela pode ser cadeirante, mas pode ser lucida. Cê acha que ela tem essa opção de escolha?

EP 3: Tem! Tem sim! Todas têm! Eh, por exemplo, quando a gente vai fazer algum passeio. Cê pega uma cadeirante que não é lúcida por exemplo. Eh, e no dia do passeio, ela fala: eu não vou! Eu não vou! Eu não vou! Eu não vou! Eu não vou! Aí a gente respeita, ela não vai. Aí tem vez que ela fala: não, eu quero ir. Eu não vou, entendeu?! Então assim, a gente respeita, essa parte delas. Só, eu acho que, é, é (gaguejou) ma mais difícil elas conseguirem tomar uma decisão da... da vida civil delas. Né?! Em questão de dinheiro, de, de eu quero comprar isso, eu quero compra aquilo. Por exemplo, (aham) no Natal do ano atrasado se eu não me engano. Veio um grupo perguntar as idosas o que que elas queriam, então tipo, perguntou pra uma cadeirante que ela foi cozinheira e tal, o que que cê quer ganhar? Ah, eu quero um copo de liquidificador, então tipo assim cê, cê consegue sabe. Só que ela precisava de outras coisas, então cê vê, e ela, as vezes ela fala: eu não quero participar dessa atividade. Mas ela não consegue tipo falar: nossa, tipo tô precisando de uma camisola, tô precisando de um chinelo. Ela não consegue falar isso. Entendeu? Mas aí ela pede um copo de liquidificador de presente. Que ela nem vai usar né?! Então assim, acho que depende muito da situação assim. Em relação à atividade assim, eu acho que elas manifestam sim. Mesmo as não lucidas assim, elas manifestam: não quero ficar, me tira daqui, quero sair, tô cansada. A gente tira, só aaacho, o que pega mesmo é a questão de resolver alguma coisa da vida civil mesmo, que eu acho que é mais difícil assim.

*Pesquisador 1: E pra assim, rotinas da casa, você acha que, por exemplo comer, tomar banho, rotinas da casa. Elas decidem ou não decidem, elas escolhem?

EP 3: Nossa mãe (suspiro)! Vamo vê... Eu sei que tem uma que não gosta de tomar banho, mas precisa tomar banho. Ou aquela que vai lá e molha o corpo e fala que tomou banho, mas cê sabe que se ela não tomou banho. Eh, mas assim, na hora que cê vai falar com ela, ela acha ruim porque ela fala com cê que ela já tomou banho, mas cê sabe que ela não tomou. Eh, tem as outras que aceitam, eu acho que as que aceitam, sabe que precisa do banho. Eu acho que..., na verdade todas sabem né, que precisa do banho. Só

que tem uns que não, tipo a que vai pro banheiro, entra debaixo do chuveiro e fala que tomou banho. Na cabeça dela, ela tomou banho memo, que ela enfiou debaixo do chuveiro, ela tá de banho tomado. Aí acho que ela não consegue perceber aí, que ela não tomou banho, que ela precisa (aham/limpando a garganta) passar o sabão, limpar (aham/limpando a garganta). Aí eu acho, que ela não consegue, mas aí também se ocê vai falar com ela, ela acha ruim. Acho difícil! Ah talvez as cuidadoras podem... falar melhor porque elas tão ali com elas todo dia né, tipo, dando banho né?! Então, elas acompanham ali o banho. Eh, o que chega pra gente ali na verdade de de problema de banho, são só duas: é uma que enfia debaixo do chuveiro e fala que tomou banho e a outra que grita porque não gosta de tomar banho. As outras, a gente não, a gente não chega até a gente reclamação que não quer tomar banho, que não gosta, que tá batendo, que tá brigando, não chega. São só essas duas aí, então assim, eu acho que elas não importam assim de fazer higiene, acho que elas não, as não lucidas né, as lúcidas mesmo as cadeirantes, elas incomodam??, elas gostam, elas pedem, até...
