# Supplementary material for: Care for older adults with disabilities in Long Term Care Facility
Source: Rev Bras Enferm. 2023 Dec 8;76(Suppl 2):e20220767. doi: 10.1590/0034-7167-2022-0767 (PMC10704689; doi:10.1590/0034-7167-2022-0767)
Supplement: 0034-7167-reben-76-s2-e20220767-suppl14 [file 0034-7167-reben-76-s2-e20220767-suppl14.pdf]

EP 7

1) Pesquisador 1: **Como é, para você, trabalhar em uma ILPI?**

EP 7: Olha, pra mim tá sendo uma experiência muito grande e válida. É... eu nunca tive experiência nessa área, tô há quase 2 anos né, que eu tô tendo a oportunidade de ter essa experiência. É... é uma experiência muito desafiadora porque assim não é uma coisa que eu pensava em trabalhar, eu venho de serviço de urgência e emergência. E... é desafiador pelas particularidades mesmo da saúde do idoso né?! Relacionadas a... a tudo que a gente aprende mesmo que são, é, tem que levar em conta o histórico de vida delas, as particularidades mesmo de... de fragilidades dela, então pra mim tá sendo desafiador. Hoje claro que eu sei mais do que ontem, mas é uma busca constante por um aprendizado, uma aprendizagem maior e um desafio a cada dia que eu chego aqui.

2) Pesquisador 1: **Me fale um pouco sobre seu relacionamento com os idosos que residem aqui.**

EP 7: Meu relacionamento é supertranquilo com todos os idosos, uma relação de parceria que eu né, estipulei com eles, nesses quase dois anos de trabalho. Eu deveria ter uma relação mais próxima, mas a questão burocrática toma muito meu tempo. Então assim, se for parar pra analisar nesses quase dois anos que eu trabalho aqui, é... pode se considerar que de uns quatro meses pra cá que eu tô tendo um relacionamento do jeito que eu deveria ter com os idosos. Porque a questão burocrática sempre me consumiu muito e na carga horária que eu executo, tava muito complicado deu saber dividir. Então hoje, eu consigo dividir e... e... ter noção do que se passa na casa, noção da saúde, dos acontecimentos com todos os idosos e todos os dias eu me programo pra atender um em... fora de atendimento de intercorrência. Então assim, um em atendimento eletivo mesmo e aí, as intercorrências eu vou apagando incêndio no meio das 4 h por dia. É... então tô me organizando mais de um tempo pra cá, maio do ano passado eu me tornei referencia técnica, então assim a burocracia já era muito, é grande e com o RT ficou maior ainda. Então assim, isso me consome muito e estava me deixando muito triste porque a parte burocrática exigia muito de mim e eu enquanto enfermeira não tava dando o que eu poderia dar pras idosas. Hoje estando mais próximas, não tanto quanto eu deveria, mas mais do que antes, eu sinto que a minha atuação já faz toda diferença.

3) Pesquisador 1: **Qual a sua percepção sobre a relação dos idosos institucionalizados com seus familiares e amigos?**

EP 7: A relação de... dos idosos com... vínculo entre amigos e familiares né?! Muito escassa! É... das 28 idosas que tem aqui, pode-se olh... observar ali pelo livro de visita, porque eu não fico... Meu horário é de manhã, então não pego muito os horários de vista, nem final de semana, mas quando eu pego pra ler o livro de registro de visitas, são pouquíssimas que recebem visitas assim, constantes de amigos ou familiares. Eu posso tá chutando aqui um número muito equivocado, mas das 28, se umas 8 tiver vínculo assim efetivo, gente que vem todo mês, que cê vai ali e vê o nominho deles mesmo constantemente, é no máximo 8. As demais, ou não recebem, não tem esse vínculo mesmo, o familiar já tá totalmente quebrado, já não existe mesmo família pra tentar reconstituir, e quando tem, é... não é fortificado esse vínculo. E amigos, muitas vezes são amigos já idosos também, então assim, que não tem condição de tá vindo na casa pra tá visitando. Então, vínculo com amigos e familiares é totalmente precário já, totalmente estremecida essa relação. Assim que eu vejo...

*Pesquisador 1: Tem muitas que saem né?! Da casa?

EP 7: Isso!

*Pesquisador 1: Você acha que... isso é uma coisa que... por exemplo a Yara é uma que...

E7: tem um vínculo muito bom familiar!

*Pesquisador 1: muito bom!

E7: Isso!

*Pesquisador 1: Você acha que assim, ela é uma das que tem mais vínculo ou cê acha que outras também tem?

EP 7: A dona Elvira tem muito vínculo familiar, o filho vem assim, por semana, que eu vejo, o horário que eu tô aqui, pelo menos umas duas a três vezes por semana. Ah... Isabel Bernadete tem uma amiga que toma conta da finança e resolve todas as questões dela, que pelo menos, uma vez por mês ela vem aqui, que ela me pergunta né?! Vem

trazer dinheiro pro espessante, pras coisinhas particulares da Isabel. Ah... Dona Elvira vai pra casa dos familiares, não com, não é por mês assim, mas em datas, aniversario, Natal, eles sempre procuram tá buscando ela. É... a Eugênia recebe visita da sobrinha, não tão constantemente, mas eu vejo algumas vezes...

*Pesquisador 1: Cê acha que tem algum motivo para não existir esse vínculo?

EP 7: Olha, eu acho que a institucionalização, como a gente aqui dá conta de resolver a maioria dos problemas dos idosos e o familiar entraria nesse, nesse lugar que a gente não substitui né?! Enquanto família, a gente substitui enquanto equipe, cada um com seu saber ali multiprofissional, nos cuidados diários né, na ajuda ao banho, ajuda na alimentação. Então, como a gente supre a maior parte da demanda dos idosos, a família se coloca numa posição assim, de distanciamento porque entende que tudo tá sendo feito. Só que o que eles podem fazer a gente não tem como dar pros idosos e eu acho que eles se colocam nessa posição de distanciamento, p... porque a gente tá suprindo isso, as demandas dos idosos. Eu entendo dessa... quem tem vínculo e a família não é presente, eu vejo dessa forma. Que quando acionada, eles vão vir aqui, é como, é... na pressão, acho que eles vêm sim. Se pegar o telefone, ligar e falar: olha, fulano tá falando muito de você, acho que cê deveria vir aqui fazer uma visita. Acho que sensibiliza pra eles virem, mas aguardar a vontade própria pra tá vindo, é... não vem por causa das, das ocupações do dia a dia deles e por que aqui as necess... (inaudível) dos idosos tão supridas, a gente consegue atender a maioria delas. Eu vejo o distanciamento dessa forma...

*Pesquisador 2: E a instituição, ela, ela promove ess... a socialização do idoso em outras áreas, assim... se o idoso sai, se vem gente fazer alguma coisa com o idoso...

EP 7: Oh, aqui, como uma instituição filantrópica, a gente recebe muitos voluntários na casa. Então, tem atividades já programadas que são fixas do mês, então a gente recebe Carinhólogos Solidários que vem no segundo sábado, a Turma do Tio Flávio que liga agendando, turma de jovens da CDL, de curso. Então assim, várias atividades são promovidas, no meio dessas atividades envolve bingo, atividades de lazer mesmo, tarde da beleza, é lanche especial, comemoração de aniversários do mês. Então assim, muitas coisas são promovidas por essas turmas de voluntários fixas. É, a instituição tá com um trabalho que tá em desenvolvimento, aí a assistente social vai sa... saber falar melhor com vocês, mas de.... buscar essas famílias. Quem tem um vinculo familiar, a gente

entrar em contato, marcar um final de semana pelo menos a cada 2 meses, pras idosas estarem indo, custeados pela própria idosa porque elas tem a renda, que as vezes né, o familiar pode falar: ah não, a gente não tem condição de receber porque né, tem que preparar, as vezes, uma comida mais diferente, com, menos, assim fazer um preparo mesmo, tem que ter um tempo pra tá recebendo o idoso. A assistente social tá trabalhando essa questão com as famílias, de tarem recebendo essas pessoas, que é uma demanda das próprias idosas, que sentem saudade das suas casas. A maioria dos familiares ainda moram onde a idosa residiu, então pra tá proporcionando isso. Dessa questão do encontro familiar com quem ainda tem o vinculo e essa questão de... de socialização, esse pessoal que vem na casa promove e como a Prefeitura de Belo Horizonte tá muito ligada aqui. A esposa do prefeito veio aqui na casa no inicio do ano, se não me engano, no final do ano passado, ele tá muito ligado a essa questão social, aí, é, promovendo... Ela deu ingressos pra... como... é um evento, não me lembro exatamente o que é, mas acho que foi uma orquestra que veio aqui no Palácio das Artes incluindo até camarote pras idosas. Então assim, algumas atividades via prefeitura, com essas parcerias que a gente tem feito e fortalecido ultimamente, elas têm se socializado bem mais do que quando eu entrei aqui. Então isso, essa questão tá melhorando... né?! Essas atividades promovidas, a assistente social entrando em contato com a família, eu vejo um movimento que não existia quando eu entrei aqui.

4) Pesquisador 1: **Você considera que os idosos dessa ILPI têm condições de tomar decisões sobre as coisas que precisam fazer em seu dia-a-dia? Por quê?**

EP 7: Essas grau I que a gente fala, são as que andam, as que conseguem gerir totalmente sua vida, sim!

*Pesquisador 1: E as outras?

EP 7: As outras, parcialmente! Então assim, tanto que a, a maioria, quase que absoluta das demais, o dinheiro delas é gerido ou por alguém da família, ou por alguém muito próximo que tenha a questão de curatela da, do idoso ou pelo escritório central que tem uma pessoa que é responsável por tá com o cartão das idosas, tá recebendo e comprando os insumos necessários. Então assim, as demais porque aí, as grau I são 7, 8 se não me engano, elas tem total autonomia e independência pra tá resolvendo essas e outras questões relacionadas à vida delas. As demais, não, parcial, mas a maioria não. Então

assim, pra receber, pra fazer a compra de medicamento, é... essas ques... pra, pra comprar uma fruta de preferencia, um medicamento que foi passado numa intercorrência, tudo isso é a gente que tem que fazer por elas. Então, só as grau I que a gente fica tranquila que elas dão conta de resolver essas questões. As demais, em algum determinado momento, a gente tem que entrar com algum auxílio porque vai falhar em algum momento.

*Pesquisador 1: E sobre atividades, assim, do dia a dia mesmo, comer, tomar banho, é... escolher se quer assistir televisão se não quer... Alguma dessas coisas, cê acha que elas, que elas escolhem aqui dentro, as grau II ou grau III.

EP 7: Na maio... a grau II ainda tem um pouco dessa percepção de, de, de, de escolha né?! De poder de decisão, as demais não. Se você colocar sentadinha vendo um programa, aí ela vai ficar, não sei quando começar a incomodar, ou seja, uma coisa que não era do hábito mesmo ou que ela não tá gostando, ela vai sinalizar e a gente vai retirar daquele ambiente. Mas a maioria segue a rotina institucional sem reclamar, sem ponderar, porque tá acostumada a ser dessa forma, porque não tem esse poder de decisão, ou já tem algum grau de demência que não vai... fazer diferença aquilo na vida delas. Não vai saber decidir sobre o que ela quer naquele momento. A maioria, não.
