# Supplementary material for: Care for older adults with disabilities in Long Term Care Facility
Source: Rev Bras Enferm. 2023 Dec 8;76(Suppl 2):e20220767. doi: 10.1590/0034-7167-2022-0767 (PMC10704689; doi:10.1590/0034-7167-2022-0767)
Supplement: 0034-7167-reben-76-s2-e20220767-suppl15 [file 0034-7167-reben-76-s2-e20220767-suppl15.pdf]

EP 9

1) Pesquisador 2: **Como é, pra você, trabalhar em uma ILPI?**

EP 9: (tempo pra pensar). É muito bom, é desafiador, pessoas de famílias diferente, e problemas diferente, mas eu sinto bem, eu ajudo pessoas que precisa, né?!

2) Pesquisador 2: **Me fale um pouco sobre seu relacionamento com os idosos que residem aqui.**

EP 9: As que são de grau I, a gente conversa muito, né?! Batendo papo, assim, e o dia que eu tô de plantão, é os cuidados normais, profissional e como técnica de enfermagem, prestar cuidados, ajudar no que que precisa. E o dia que eu não tô também, como eu moro na casa, no dia que eu não estou em serviço, o meu tempo eu passo na casa e as vezes a noite a gente fica conversando, vendo televisão. Então é como eu fico 24 horas na casa, então, mais assim numa casa como minha família. Como se fosse fazer parte da família, porque eu moro aqui, eu fico 24 hora dentro da casa.

*Pesquisador 1: A senhora mora aqui?

E9: Eu fico 24 hora, então fica, quando vai chegando uma certa tempo, parece que já sente parte da família da gente, porque é, eu fico sabendo de tudo que acontece na casa, o que condiz lugar propicio na verdade, ah vou descansar porque eu tô fora da casa, mas eu fico 24 hora dentro da casa, os problemas que acontece de dia, de noite plantão meu ou não, eu tomo conhecimento de tudo, diferente das outras funcionárias, que faz 12 horas, vai pra casa e não tem mais contato com elas, até a volta, quando chega já passou muita coisa e elas não tão sabendo, no meu caso é diferente, eu fico 24 horas.

*Pesquisador 1: Senhora acha isso bom?

EP 9: Eu acho, é, principalmente quando é, as vezes as pessoas questionam porque eu sou irmã, trabalhar de enfermagem, falei eu prefiro, vou sair, ficar fora disso, pra mim já não faz, porque aqui eu vejo que eu consigo ajudar elas melhor no meu cargo de enfermagem, pelo, a cada dia o meu conhecimento, aquilo que eu vou aprendendo com elas, né?! Com os profissionais, é me ajuda também, a ajudar quando, por exemplo, quando não tem uma técnica de enfermagem, sou eu que sou, fins de semana que não tem, só tem cuidador a noite, eu que sou a técnica da casa. Então acaba eu, acabando respondendo por isso.

3) Pesquisador 2: **Qual a sua percepção sobre a relação dos idosos institucionalizados com seus familiares e amigos.**

EP 9: Isso varia muito, né?! De idoso pra idoso tem umas que vem alguém pra visita outras já menos, né?! Então isso aí é um caso muito, muita diferença um do outro, tem umas que vem mais visita, outros já vem menos visita, outros quase não tem, né?! Tem, a Izabel por exemplo, ela, só vem uma senhora, que cuida das coisas dela, mas até então ainda num vi parente dela aqui. A Luzia Guedes só tem amigas, porque não tem família, a família fica distante, então ela não recebe de parente mesmo nunca teve nenhum parente dela aqui.

*Pesquisador 1: Cê acha que elas sentem isso, de alguma forma?

EP 9: Com certeza, com certeza, instituição pode ser o melhor tratamento possível, um hotel 5 estrelas, não é família, pessoas sente diferença sim, elas estão porque não tem outra alternativa, é, cê vê numa situação, as vezes a pessoa ta consciente sabe, só que não tem família, então já vem pensando que um dia pode precisar de cuidados, lá fora elas não vão ter, porque não tem família, é, já perdeu irmão, perdeu pais, então, vem porque não tem outra alternativa, porque se sente é, eu costumo dizer que a família você pode ter o seu pior lugar, mais ainda é melhor do que uma instituição, que tem todos os cuidados, porque é um calor de família, é um calor humano, né, familiar. Como aqui tem muita gente, uma é de uma família, um reage de uma maneira, outro reage de outra maneira e elas tem que acabar se acabar se ajeitando do jeito que elas podem, sendo que uma é de uma família outra é de outra, um veio de um lugar o outro veio do outro, mas elas tem que se ajustar naquele espaço, um dia um funcionário, outro dia é outro, outro dia é outro, uma trata com muito carinho, a outra já na rotina, já deixa um pouco a desejar e elas tem que aguentar, umas tem problema psicológico, outras não tem, umas a gente tem que ter muita calma porque, não sair do controle, elas são bastante, né?! Assim agitadas, né?! No dia a dia delas, e assim a gente tem que lidar com tudo isso, no decorrer do dia.

*Pesquisador 1: É realmente desafiador, né?!

EP 9: É um desafio, aqui de vez em quando você levanta é um desafio até o dia que eu fico, eu tô na reponsabilidade, aí muita gente fala assim, que eu fico diferente, né?! No dia de amanhã. Aí eu fico pensando eu tenho que fazer aquilo, tem o remédio depois, tem uma coisa pra fazer, um curativo que tem que fazer, tá chegando alguém, entendeu?! É uma rotina, então as vezes eu tenho essa mania, de fazer isso e fico diferente, é, que

amanhã eu tô livre, sossegada, não tenho responsabilidade com nada, o dia de amanhã, também, por exemplo se ocês vem aqui, eu tô livre, posso ficar aqui horas, agora hoje com o plantão corre o risco de tá me chamando, né, uma vai comer, engasga e tá atrás de mim me chamando, uma outra caiu lá. Outro dia, até o rapaz que teve aquele problema também, que foi no meu plantão, cê ficou sabendo? Então caiu justamente no meu plantão. Então essas coisas a gente acaba tendo que atender e acaba sendo bem desafiador, né?! Não é tão simples assim, chegar, a uma maravilha, é muito bom, mas tem os desafios, aí vem as consequências daquilo que a gente assumiu.

4) Pesquisador 2: **Você considera que os idosos dessa ILPI têm condições de tomar decisões sobre as coisas que precisam fazer em seu dia-a-dia? Por quê?**

EP 9: Algumas sim, outras não, tem umas que são conscientes, as que são conscientes, é, nada impede tá tomando decisões, principalmente que se a gente tira isso, de quem tem condições disso, de exercer, a gente tá tirando a própria liberdade da pessoa e isso as vezes eu acho que pesa muito em ILPI, por exemplo tem uma moradora que se dá conta de tomar um medicamento sozinha, só que por ser numa ILPI, a gente tem que tirar delas, aquilo que elas tava acostumada a fazer em casa, e aqui a gente chega e tem que tirar, porque tem as fiscalização, tem a vigilância. Então elas não sente humilhadas com isso, então a única coisa que elas fazia, elas mesma tomava o medicamento delas e aí elas sentem o que, isso tira também muitas coisas que as vezes elas poderia fazer sozinha e quando chega aqui, vai diminuindo essa possibilidade delas tá fazendo. É, hoje mesmo eu tava comentando com uma cuidadora questão de uma moradora que chegou aqui ela passava da cama, é passava da cadeira pra cama, na casa dela sozinha, hoje a mulher não tem condições mais de sair da cadeira e ir pra cama, porque, vai cair, vai machucar, aí depois vai cair sobre mim, então, eu te passo pra cama, beleza, eu vou passar pra cama, só que eu achei que ela regrediu muito depois que chegou aqui, esse é um caso ou outro caso. Eu comentei isso com a cuidadora, hoje de manhã, eu falei: “nossa ela chegou aqui, na casa dela se virava sozinha, ela fazia comida, se queimou, porque tava fazendo comida, se queimou porque tava fazendo comida, tava se virando sozinha, hoje aqui o pouco que ela fazia, como ela chegou numa instituição, hoje ela não consegue fazer mais, hoje mal ela consegue pegar numa colher, né.”

*Pesquisador 1: E a senhora acha que isso é uma consequência de tá institucionalizada?

EP 9: Porque ela se esforçava a fazer aquilo que ela dava conta, e hoje tem muita gente pra fazer coisas que aquilo que ela da conta de fazer, tem alguém pra fazer. E o ser humano, tudo que ele vai diminuindo assim, se já tem dificuldade de locomoção, quanto mais você ajuda, menos você, ele vai se esforçar de fazer e acaba prejudicando, é, as vezes deixa a moradora pegar a colher pra almoçar, o que ela não dê conta, que eu vou ajudar. Porque tudo aquilo que eu chego e falo pra me adiantar eu vou colocar comida na boca, então isso, eu tô prejudicando, né e as vezes tem umas moradoras, que pede: "faz isso pra mim" eu falo: "não, você vai fazer, você da conta, no dia que você não de conta, você me fala, que eu faço, agora você da conta, você vai fazer", então eu mudo a posição, diante de que, agora ela é uma paciente, a coisas mudaram, mas ela é uma paciente, então ela fala: "faz isso pra mim" "não você dá conta". Tem uma que ela fala, né?! Ah, mais ocês num me ajuda a fazer isso, falei "ajudo no que você dá conta, se você não desse conta eu faria, mais eu sei que você da conta", "você vai fazer", ela pega e faz.

*Pesquisador 1: Irmã você acha que todo mundo tem esse pensamento, que trabalha aqui?

EP 9: As vezes mais pela rotina, não vou dizer a você que tem assim, que na verdade assim, eu, é, não vou acompanhar, não acompanho todos os plantões pra falar: "deixa que ela faça", mais aí eu sempre oriento quem tá cuidando é, falo olha: "isso você não vai fazer pra ela, porque ela dá conta de fazer"" se você faz isso pra ela hoje, amanha ela não vai fazer mais, porque você fez". Ela virou pra mim e falou: "mas a fulana faz" eu falei: "mas eu não vou fazer" "irmã cê tá ruim demais pra mim" "tô, tô muito ruim, e você vai fazer" aí ela virou pra mim e falou: "você é teimosa hein irmã?!" falei: "sou, é pro seu bem" aí ela pega, vai e faz principalmente no horário do banho, porque eu ajudo e coloco o sabão direitinho, mas quem faz restante é ela, aí aquelas partes que eu sei o que posso ajudar, que é meu dever de ajudar, eu ajudo, mas aquilo que eu sei que é delas, aí é você. Mesmo que ela fala comigo, "eu não vou fazer não que a fulana faz pra mim" "não tem problema, amanhã ela ajuda, mais hoje eu não vou te ajudar. Eu acho sabe que quanto a pessoa puder fazer, vai tirando, tem muitas que são acomodadas, né?! Arruma isso pra mim, faz isso pra mim, principalmente tem umas que gostam de chamar a atenção né, me ajuda a fazer a isso, me ajuda a fazer aquilo, que a gente vê na cara que da tranquilamente de fazer sozinha, mas como tem alguém por perto, quer que alguém fica por perto, aí então tem esse jeito de falar me ajuda.

*Pesquisador 1: É as vezes elas nem tem essa consciência, de que isso de certa forma tira um pouco essa liberdade delas, essa autonomia delas.

EP 9: É, eu vejo muito isso, é, que quando alguém tá fazendo pra mim melhor né?! Que não precisa deu fazer e as vezes na rotina, ajuda a gente acaba, a hoje tem muita coisa pra fazer, então eu mesma faço porque já vai logo, porque se for esperar é lento, cê tem que ter uma demanda de tempo, pro cê tá esperando, quer dizer tempo da pessoa e aquilo que, quando eu deixo, quando eu faço, eu faço no meu tempo, que eu corro, rapidinho eu encero, agora quando eu vou esperar uma pessoa que tem dificuldade de movimento, é muito mais lento e aí as horas vão passando.
