# Supplementary material for: Care for older adults with disabilities in Long Term Care Facility
Source: Rev Bras Enferm. 2023 Dec 8;76(Suppl 2):e20220767. doi: 10.1590/0034-7167-2022-0767 (PMC10704689; doi:10.1590/0034-7167-2022-0767)
Supplement: 0034-7167-reben-76-s2-e20220767-suppl16 [file 0034-7167-reben-76-s2-e20220767-suppl16.pdf]

EP 13

1) Pesquisador 1: **Como é, pra você, trabalhar em uma ILPI?**

EP 13: Olha, pra mim está sendo algo muito novo né?! Porque eu vim duma área comercial, tô numa área humana né, de cuidado, de atenção, de minúcias com o ser humano, então pra mim assim, eu gosto, sempre tive um excelente relacionamento com idosos, né, então tô, tá muito gratificante pra mim.

2) Pesquisador 1: **Me fale um pouco sobre seu relacionamento com os idosos que residem aqui.**

EP 13: Relacionamento é muito bom, é eu sou paciente, sou ouvinte, sou carinhosa, sou dura também, mais assim, elas sabem diferenciar o meu ser, o que eu acho mais engraçado é isso, elas já sabem quem eu sou, sabe?! Consegui expor pra elas quem eu sou e como eu sou, então acho isso muito bacana, que elas já falam: "É a Tetê?" Pela minha voz, pelo meu jeito de entrar, pelo meu jeito de fazer as coisas, sabe?! Elas já sabem que sou eu.

3) Pesquisador 1: **Qual a sua percepção sobre a relação dos idosos institucionalizados com seus familiares e amigos?**

EP 13: Olha eles tem um relacionamento bom, é, familiares as vezes são muito ausentes, os que são mais presentes, assim você vê que eles sentem é, o sentimento deles é diferente dentro da instituição, não ficam revoltados, não ficam agressivos, né, num tratam a gente com indelicadeza, geralmente aqueles que, ou então tá com Alzheimer muito avançado, aí num tem como, independente de ter familiar ou não, ele não tem muita resposta né, mas a gente vê a diferença daquele que não recebe a visita, daquele que as vezes tem só a gente mesmo como familiar né, eles são mais tristes, olhar triste, né, as vezes tá mais queixoso, é as vezes é, pode gerar algum tipo de doença, algum tipo de mania, chama muita atenção, né.

*Pesquisador 1: Tem algum motivo pra essa ausência de amigos e familiares?

EP 13: Olha, eu não sei te dizer, porque eu tô recente, então eu não sei o longo período que a pessoa entrou né, que aqui tudo vem da entrada, né, então eu não sei, te dizer assim, ao certo o que causa isso, o porquê que, né, alguns a gente escuta alguns comentários, mas outros eu não sei te dizer.

4) Pesquisador 1: **Você considera que os idosos dessa ILPI têm condições de tomar decisões sobre as coisas que precisam fazer em seu dia-a-dia? Por quê?**

EP 13: Olha, algumas sim, outras não.

*Pesquisador 1: Por quê?

EP 13: Porque algumas querem coisas que são perigosas pra vida delas, pra saúde delas, né, e outras não, são coisas mais acessíveis: "muda isso" "faz aquilo pra mim" "confere isso" alguma coisa assim, algumas querem sair, querem fazer outras coisas que já não tem essa capacidade de ficar andando sozinha com grau elevado que elas têm de, né, dependência.

*Pesquisador 1: Então cê acha que a dependência que define se elas conseguem tomar decisão ou não?

EP 13: Eu acho que a dependência, num é só a dependência, mais assim, o grau elevado, principalmente do Alzheimer, é o que mais, né, prende, como se diz elas são muito mais presas.

*Pesquisador 1: E por exemplo as cadeirantes lucidas?

EP 13: As cadeirantes a gente atende elas na medida do possível, né, assim do tempo da, né, do que, que elas desejam, né, as vezes a limitação, as vezes não é nem no sair, as vezes na alimentação, num diabetes, é uma insuficiência, né?! Então assim, é isso que mais, como se diz restringe mais elas, sabe?!

*Pesquisador 1: Condição de saúde?

EP 13: Condição de saúde, mais condição de saúde.

*Pesquisador 1: E sobre as coisas simples, por exemplo, de comer, tomar banho?

EP 13: É, aí elas já são bem dependentes da gente, né, aí a gente que coloca pra elas o que estamos fazendo, né, e assim, tem umas que consegue uma boa referencia que tomou um bom banho, que ontem não foi legal, consegue ter dinâmica com a gente, sabe?! A nossa proximidade realmente é na hora do banho, sabe?! Ai elas já pedem, como se diz, já pedem as coisas ao longo do dia, entendeu?

*Pesquisador 1: Entendi. Mais alguma coisa?

EP 13: Não, só.
