# Supplementary material for: Care for older adults with disabilities in Long Term Care Facility
Source: Rev Bras Enferm. 2023 Dec 8;76(Suppl 2):e20220767. doi: 10.1590/0034-7167-2022-0767 (PMC10704689; doi:10.1590/0034-7167-2022-0767)
Supplement: 0034-7167-reben-76-s2-e20220767-suppl17 [file 0034-7167-reben-76-s2-e20220767-suppl17.pdf]

EP 14

1) Pesquisador 1: **Como é, pra você, trabalhar em uma ILPI?**

EP 14: Pra mim é ... é prazeroso, ao mesmo lado que é cansativo, é prazeroso, porque a gente aprende a dar valor a muita coisa, querendo ou não você lidar com pessoas que já viveram muita coisa e você é, adquire muito conhecimento através delas, você vê como era antigamente, como é agora, né, o que elas passaram, são historias assim que ficam, que marcam, igual do tempo que eu tenho essa caminhada tem muita coisa que me marcou, tem muita coisa que me guardou, eu sempre lembro de alguma coisinha de cada uma.

2) Pesquisador 1: **Me fale um pouco sobre seu relacionamento com os idosos que residem aqui.**

EP 14: Eu acho que é uma relação boa, né, igual eu falo que tem minha carrapato, que num me larga, eu gosto de fazer elas rirem, é uma coisa assim, oh, a questão que eu coloco é o respeito, é de você colocar no lugar dele, do idoso, querendo ou não você tá lidando com uma pessoa igual a você, não é porque tem mais idade que não seja igual a você, é um ser humano, então cê tem que se colocar no lugar dele, porque querendo ou não, daqui a pouco você pode chegar a idade dele, pode necessitar dele, igual ele tá necessitando da gente nesse momento agora.

3) Pesquisador 1: **Qual a sua percepção sobre a relação dos idosos institucionalizados com seus familiares e amigos?**

EP 14: Olha aqui, vamos falar assim são alguns que tem, né, que tem isso. Os que tem, eu acho que é o prazer de reencontrar alguém, quando fica muito tempo sem vê, quando vem visitar as amigas, a pessoa lembra, mesmo que tenha um déficit, né, seja Alzheimer, seja Parkinson, seja a demência mesmo de idade, sempre lembra de alguma coisinha, sempre acarreta a memória, a tá lembrando, a questão relacionamento é bom, graças a Deus.

*Pesquisador 1: Você acha que todas elas têm essa possibilidade de encontrar familiares e amigos, de receber familiares e amigos aqui?

EP 14: Algumas são sozinhas, né, então não tem como, colocar assim, são sozinhas no mundo, não tem ninguém por elas. E as vezes, a gente sabe que tem algum familiar quando vem a falecer, que aí eu não sei da onde surge assim sabe, é muito interessante,

assim, deixa a gente sem saber, uai, quando tava aqui ninguém aparecia, chega a falecer, aparece, porque que não veio em vida, né?!

*Pesquisador 1: Cê acha que a instituição de alguma forma ela estimula isso?

EP 14: O que?

*Pesquisador 1: O contato delas com familiares e amigos.

EP 14: Sim, estimula, querendo ou não cobra também, né?! É cobrado o sumiço, mesmo que seja pra, pra pedir algo né, pra, pro próprio idoso, tem também a questão de a presença, é tem uns se for o caso de, é, parente mais próximo de primeiro grau né?! Um filho, ou um sobrinho, aquela coisa toda, aí começa a cobrança também, aí tem que cobrar, mais graças a Deus os que tem são muito presentes, ou vem todo final de semana ou vem, igual o filho da dona Elvira vem durante a semana, a possibilidade que ele tem ele tá vindo, a filha dela teve aí final de no semana, não foi no meu plantão, mas tá escrito no relatório que teve, né, a filha trouxe café da manhã pra ela.

4) Pesquisador 1: **Você considera que os idosos dessa ILPI têm condições de tomar decisões sobre as coisas que precisam fazer em seu dia-a-dia? Por quê?**

EP 14: Algumas sim, outras não, rs.

Pesquisador 1: Por quê?

EP 14: Porque igual a questão, quem tem Alzheimer vai decidir por ela, né?! Algumas tem a questão da demência também, não fala coisa com coisa.

*Pesquisador 1: E de coisas simples do dia a dia, por exemplo como comer, tomar banho, onde quer ficar.

EP 14: Ah isso sim.

*Pesquisador 1: Você acha que todas tem, essa condição de decidir?

EP 14: Tem, eu falo assim aquelas que se responde por si, sim, as outras que não tem como responder que é um hábito da gente, igual eu falo uma questão quem é cadeirante, a gente tem que auxiliar, né, em tudo, o caso da Bel, a Bel a gente tem que a, ela não come sozinha, então a gente que da o alimento pra ela na boca, então ela não tem como responder, né?! Mas as outras sim.

*Pesquisador 1: Você acha que todas as outras sim, tirando a Isabel?

EP 14: Não, eu falo em relação as independentes, né, que as que tem a cabecinha também melhorzinha.

*Pesquisador: E cê acha que a decisão tá ligada a lucidez?

EP 14: Porque igual ela, ao mesmo tempo que tá, assim tranquila, ela tá agressiva porque ela tem Alzheimer, então as vezes você chama ela pra almoçar “eu não quero, que não sei o que” aquela birra toda, né, faz birra, faz birra, não faz birra, mais acaba indo, com jeito, né, chamando.
