# Supplementary material for: Care for older adults with disabilities in Long Term Care Facility
Source: Rev Bras Enferm. 2023 Dec 8;76(Suppl 2):e20220767. doi: 10.1590/0034-7167-2022-0767 (PMC10704689; doi:10.1590/0034-7167-2022-0767)
Supplement: 0034-7167-reben-76-s2-e20220767-suppl18 [file 0034-7167-reben-76-s2-e20220767-suppl18.pdf]

EP 18

1) Pesquisador 1: **Como é, pra você, trabalhar em uma ILPI?**

EP 18: Sob aspecto de vida, sob aspecto é, pessoal, sob o aspecto profissional, porque tem vários aspectos, né?!

*Pesquisador 1: Todos.

EP 18: Bem, sob o pessoal, né, como pessoa, como ser humano, é, eu gosto porque você tem contato com essas pessoas que são dependentes socialmente falando, sob o aspecto médico, físico, né?! Então da um prazer muito grande, de poder, vamos dizer assim, nos dar um pouco a essas pessoas, nem tanto por questão financeira, né, eu não precisaria estar aqui por questões financeiras, nem nunca precisei, mais sempre pelo lado de gostar de doar uma parte do conhecimento, né?! Pelo prazer do contato, são pessoas carentes, aliado também claro no aspecto técnico, né?! Então como geriatra, claro, tenho conhecimento dessa área, eu acho que eu posso contribuir sob o aspecto técnico, acho que associa essa questão pessoal, do prazer, né, de ajudar o ser humano idoso, carente e também prestar essa doação com o conhecimento técnico cientifico. Eu não trabalho só nessa ILPI, eu trabalho em outra ILPI, já trabalhei em várias, hoje eu tô em duas.

*Pesquisador 1: Todas filantrópicas? As duas filantrópicas?

EP 18: Essa filantrópica, outra filantrópica e já trabalhei em particulares também, mas as filantrópicas que me dá mais prazer de lidar, de trabalhar, tanto é que nessa outra também, já deve ter uns vinte cinco/trinta anos que eu tô lá também.

2) Pesquisador 1: **Me fale um pouco sobre seu relacionamento com os idosos que residem aqui.**

EP 18: Olha é, bem, da minha parte logico, eu gosto de todas, mas eu vejo que todas tem um carinho, uma atenção muito grande pra com minha pessoa, tá, no sentido de confiança delas na minha pessoa, um respeito pela minha pessoa, né?! E reciprocamente também, né, também a respeito, gosto muito delas, né, e não é, um dos motivo deu estar aqui nessa instituição filantrópica é justamente né, esse prazer de lidar com essas pessoas tão carentes, mas ao mesmo tempo extremamente carinhosas, né, um caso ou outro, igual da dona Fia que é um caso psiquiátrico, mas grande parte delas, aceita essa condição de vida que elas tem, né, e elas tem essa confiança muito grande em mim, geralmente nos médicos que trabalham nas instituições, essa relação é mais ou menos assim, né?! Então eu tenho

profundo respeito, carinho e de certo modo até pena delas também, né, por não terem familiares, né, e aqui ser o ultimo reduto da vida delas, e muito delas estão há décadas aqui dentro também, né, a vida, até antes deu chegar tinha uma delas aqui, algumas delas. Então essa questão de respeito, de carinho com elas, né, de sempre achar que pode fazer alguma coisa pra melhor, né?!

3) Pesquisador 1: **Qual a sua percepção sobre a relação dos idosos institucionalizados com seus familiares e amigos?**

EP 18: Olha, essas aqui especificamente, a maioria delas não tem família, grande parte delas, que elas, a casa Santa Zita, Santa Zita é uma santa, né, que era protetora ou enfim, né, dessas empregadas doméstica, Santa Zita tem essa origem. Então muito delas eram empregadas domésticas e que desde sempre nunca tiveram contato familiar, elas moravam em casa das próprias famílias, foram abandonadas pela família de origem, foram morar na casa das famílias e depois vieram pra cá, né?! Então eu vejo assim que grande parte delas, não só agora, nessas três quase quatro décadas que eu tô aqui, eu vejo que a presença de familiares aqui nessa instituição e também na outra que eu trabalho é muito pouca, né?! É, eu acho que o relacionamento social que elas tem, são com as próprias pessoas da instituição e uma ou outra que as vezes tem um sobrinho, a maioria é um sobrinho, né, grande parte delas assim e um amigo ou outro, né, um voluntario que vem, mas eu não vejo elas assim com um relacionamento social mais amplo, né?! Então a diferença assim, um pouquinho da instituição filantrópica para as particulares, é que as particulares, mesmo que a família não queira, sempre a um familiar por trás, filho, neto, sobrinho, até por questão financeira, nas filantrópicas, muitas vezes você já não tem essas famílias, uma ou outra que tem família, mas a grande maioria não tem, eu não vejo assim uma relação familiar delas aqui, nas outras instituições, né?! É, então elas são muito carentes nesse aspecto, né, geralmente são os amigos, assim, alguns amigos que ainda sobraram, que elas tinham contato no passado, né, mais geralmente são bastante ausentes, né?!

4) Pesquisador 1: **Você considera que os idosos dessa ILPI têm condições de tomar decisões sobre as coisas que precisam fazer em seu dia-a-dia? Por quê?**

EP 18: Depende do grau de independência, né?! E depende da questão cognitiva e da autonomia delas, nos temos aqui umas quatro ou cinco, seis talvez, que tem sua autonomia e independência, elas tomam as decisões delas, as vezes chego “ah dr. eu fui no Carlos

Chagas" "fui no médico" "fui ali" "vou passear, vou na Aparecida do Norte" num sei o que. Então algumas poucas, né, tem a sua autonomia e sua independência, as que podem, podem, acho até correto, a gente até libera pra que seja assim. Agora a grande maioria das outras são mais idosas, assim não no tempo cronológico, mas no cognitivamente comprometidas, funcionalmente comprometidas, aí elas não têm decisão de praticamente nada, né? Então algumas tem toda independência que quiser, não prendemos nem nada, mas outras não tem, então é a minoria que tem sua independência e autonomia.

*Pesquisador 1: E o senhor acha que essa autonomia tá relacionada a lucidez, cognitivos?

EP 18: Sim, sim, todas essas. É porque se a pessoa já é demenciada, né, ou seja, a autonomia dela já é comprometida, né?! Aí nos temos, até que ver a que ponto essa autônima pode gerar risco pra ela ou não se ela não tem discernimento das coisas né?! Mas as que podem, como as que tem aí, não tem problema nenhum.

*Pesquisador 1: Mesmo que essas decisões forem relacionadas a coisa simples, como comer, tomar banho, o cê acha que ainda assim a lucidez?

EP 18: Ah tá, essa é uma questão, principalmente com relação aqui, até pouco tempo a gente teve uma conversa sobre isso, sobre a questão por exemplo de medicação, é, nós não permitimos que nenhuma delas fique com, tá dentro da sua pergunta também, elas poderiam ficar com o remédio dentro do quarto delas? Essa decisão nós tomamos de não permitir, por quê? Porque mesmo elas tendo essa autonomia e discernimento, elas não têm conhecimento pleno, entendimento medico sobre a patologia que elas têm, como manipular aquelas drogas, a responsabilidade de tomar o remédio. Então, nós já tivemos problemas aqui de você, acreditava-se, né, que elas poderiam tomar remédio, depois você toma, passava uma semana, chegava lá uma lata de remédio lá, "ah não tomei porque tava frio, tava calor, tava assim, tava assado" então essa autonomia nós não podemos permitir que elas tenham, e nem mesmo em casa, geralmente quando eu converso com as famílias, é, eu oriento as famílias que chegam, mexer com medicação deve ser, não deve ser a própria pessoa, o próprio idoso, porque ele tem n julgamentos errados, que podem ser maléficos a ele, né?! Então, mas e outras decisões que não trazem prejuízo a eles, né, por exemplo hora do banho, acho que a hora do banho se ele tem autonomia "ah não quero tomar banho, vou tomar banho daqui a pouco" tudo bem, ou alimentação "eu não gosto disso, não quero comer aquilo" então assim, se são condutas que não trazem grande prejuízo e outra coisa também, da medicação também é a questão da dinâmica da casa,

porque as vezes, porque que nós, temos que por, a palavra foi essa, horário de medicação, porque se não, o remédio tá prescrito lá oito horas, aí ele não quer “ah não vou tomar agora não, vou daqui a pouco” não tem como a enfermeira ficar indo e vindo, um quer tomar as oito, outro oito e quinze, um dez, não tem como fazer isso. Então infelizmente, né, em todo grupo social tem que ter as normas e limites, então aí nós não podemos dar esse pleno direito de escolha, né?! “Eu quero ficar com remédio”, “eu tomo a hora que eu quero”, “eu não quero, depois cê traz pra mim”, “eu não quero” isso não tem como fazer. Agora com relação a essas outras situações, desde que não aja perigo, sair da casa, ir pra casa de família se quiser, fazer pequenas viagens, isso não tem problema, eu acho até que deve, como elas tem mesmo, tá.
