# Supplementary material for: Care for older adults with disabilities in Long Term Care Facility
Source: Rev Bras Enferm. 2023 Dec 8;76(Suppl 2):e20220767. doi: 10.1590/0034-7167-2022-0767 (PMC10704689; doi:10.1590/0034-7167-2022-0767)
Supplement: 0034-7167-reben-76-s2-e20220767-suppl19 [file 0034-7167-reben-76-s2-e20220767-suppl19.pdf]

EP 19

1) Pesquisador 2: **Como é, pra você, trabalhar em uma ILPI?**

EP 19: Bom, foi uma escolha, né?! Eu, eu, eu, eu escolhi trabalhar com idosos, então, é, ... é uma afinidade, né que eu tenho pelo público, eu gosto do público e, e resumidamente foi por afinidade, que eu escolhi trabalhar com eles.

*Pesquisador 2: E a sua primeira experiencia com idosos, é aqui nessa ILPI?

EP 19: Numa ILPI sim, eu já tinha trabalhado em CRAS e lá a gente tinha é, ace..., é grupos com idosos, só que especificamente em instituição foi a primeira opor... primeira vez.

2) Pesquisador 2: **Me fale um pouco sobre seu relacionamento com os idosos que residem aqui.**

EP 19: Aí, é, nesses quatro anos, hoje, porque passou por todas as fases, né?! De conhecer, de aproximação, é, de criar uma convivência, algumas mais próximas, outras, é, nem tanto, né?! É, então, é hoje já é uma relação, já estabelecida, é, de, de profissional, né, que passa pela amizade, né, que a gente tá aqui todo o tempo, né?! Antigamente eu trabalhava o dia todo, então, é, os dois primeiros anos eu trabalhava o dia todo, então me aproximei mais, né, delas. Hoje eu só trabalho meio horário, mais é de um profissional mais aproximado, né?! Porque você acaba conhecendo aquelas pessoas, então não dá pra ser diferente.

3) Pesquisador 2: **Qual a sua percepção sobre a relação dos idosos institucionalizados com seus familiares e amigos?**

EP 19: A minha percepção deles com os familiares e amigos? Bom, é, algumas pessoas que estão aqui, não tem mais ente familiar, né?! Então fica-se na recordação, na lembrança, é, algumas, outras que ainda tem família, é, algumas é bem próxima, bem próxima a, a relação entre eles, outras a gente vê, cada caso é um caso, né?! Porque cada pessoa que tá aqui traz uma história diferente, né?! Então cê vê um pouquinho de cada coisa, cê vê de proximidade, de participação, cê vê de, de, de ..... uma convivência, uma pouca convivência, tem isso também, mais aí cê vai pensar, porque pouca convivência? Ah, porque eles têm a vida deles, né?! E reservam um tempo, que pra eles é suficiente, né?! Pra vim fazer visita, é, é, ou levar pra passar o dia fora, né?! Eu vejo que, que, que, na realidade, se eu for avaliar na perspectiva da família, tá de acordo, pensando que

muitos tem que trabalhar, gerir as suas vidas, seus problemas. Na percepção do idoso, a gente percebe que ele acha que é pouco, né?! Então, tem algumas pessoas que gostariam de estar dentro do seio familiar, né?! Mas por alguma é, condição, né, o próprio da ILPI, né, o motivo de se ter uma ILPI, né, é, é, talvez os vínculos já estivessem frágeis ou é .... a vulnerabilidade chamou, é foi mais forte, o isolamento e acaba-se né, chegando a essa perspectiva de, de institucionalização. Então eu acho, que cada história é uma, né, então tem de tudo, tem a que, repito, aqueles que tem um bom vinculo, tem aqueles que o, a convivência é esporádica, eventual, tem, tem aqueles que não tem mais familiar, né, as vezes tem um amigo, tem aqueles que nem amigo mais tem, que já faleceu, né, ou perdeu contato, a gente tenta sempre estimular, vão ligar, vão procurar, é o nosso trabalho fazer isso, né?! Mas a gente legalmente só pode exigir certas coisas de familiar direto, né, principalmente filhos e a maioria aqui não tem filhos, né, só uma que tem filho vivo ainda, a outra que tinha filho, já faleceu. Então assim, diretamente uma só que tem, né, o resto tudo, ou é amigo ou é primo, ou é sobrinho ou é uma pessoa que trabalhou, uma pessoa que teve afinidade. Então vai depender muito de, da perspectiva, é muito geral, mais no geral, é isso.

4) Pesquisador 2: **Você considera que os idosos dessa ILPI têm condições de tomar decisões sobre as coisas que precisam fazer em seu dia-a-dia? Por quê?**

EP 19: A gente tenta estimular, né, é a participação delas, a ouvir a opinião, estimular elas dizerem a opinião, muitas aqui, pela sua história de vida, estão acostumadas só em receber ordens. Porque muitas aqui, são empregadas domesticas, né?! É, então elas têm um pouco de dificuldade, às vezes, de trazer demanda, até elas acham “ah não eu resolvo do meu jeito”, é, “não precisa incomodar”, que elas tão acostumadas a servir. E quando elas são chamadas, a emitir opinião delas, e a, a participar das decisões, da instituição, elas estranham. Mas a gente estimula, o tempo todo, aí ocê muda a forma de perguntar, a forma da conversa, o que você gostaria, como que, o que seria melhor, né, mas o perfil delas, é, é, eu percebo, que elas, elas muitas falam “o que vier tá bom”, “tá bom desse jeito”. É, mas tem outras, né, conforme eu falei, é uma diversidade, né, nós temos pessoas aqui com nenhuma escolaridade e tem pessoas com nível superior, né?! Então, isso, essa diferença a gente percebe até nessas relações, então, mais de uma forma geral, elas, elas as vezes se sentem é um pouco, ... como que eu vou falar, não é incomodadas, apreensivas, mas acham estranho, falar, dar a opinião e entender que pra gente é importante. Então a gente tenta estimular, né, são feitas assembleias, reuniões, é, tudo que é decidido a gente

tenta levar, não sei se seja alguma emergência e tem que decidir rápido, mais a gente tem tenta levar em reuniões, apresentar. Eu percebo que as vezes elas têm dificuldade de falar a sua opinião em grupo, né, porque depois, aí elas muda, vem procurar a gente no cantinho. Então, a gente percebe que diante do grupo algumas lúcidas tem a dificuldade de emitir a sua opinião, aí depois gera um grande problema pra instituição. Porque, é, as reuniões são pra deliberar assuntos né, cotidiano, assuntos é que muitas vezes num dá pra voltar atrás, né, e elas concordam e depois algumas ficam incomodadas. Então isso é algo que a gente ainda tenta trabalhar nelas, de ter esses espaços de, de tô falando das, das independentes, das lúcidas, de, de assumir esse lugar, né, de, de falar de trazer sua opinião, mesmo estando diferente da colega ou da, da casa, instituição, aos pouquinhos, né, mas até hoje eu percebo isso. Faltou alguma coisa da pergunta? Era uma pergunta grande.

*Pesquisador 2: Era sobre, se elas conseguiam tomar decisões, se você acredita que elas conseguem tomar decisões sobre as coisas que elas precisam fazer no dia a dia?

EP 19: Sim.

*Pesquisador 1: Cê acha que essa condição, é só pras lucidas ou pras não lucidas, também?

EP 19: As dependentes sempre participam da reunião, sempre estão, mas é, raramente, elas conseguem discernir o que tá sendo falado. É, é, cê as vezes pergunta, aí pela resposta, cê percebe que ela não entendeu, que ela, não tá claro. Por mais que cê tenta falar o português mais claro, mais simples, as que já tem um déficit cognitivo, uma dificuldade ou a demência propriamente dito, que é o que mais a gente tem, elas não conseguem perceber.

*Pesquisador 1: Cê acha que isso também se refere a atividades cotidianas? Por exemplo, de comer, de tomar banho, de escolher onde quer ficar, cê acha que esse tipo de atividade, elas conseguem escolher?

EP 19: Escolher eu não sei, porque uma vez que tá numa instituição, por mais que tenha sido é, é, vamos falar o banho, tem idosa que gosta de toma banho quatro horas da manhã, cinco horas da manhã. Mas tem idosa, que quer tomar o banho mais ao longo do dia, se ela for independente não tem problema, mas se ela depender das cuidadoras, ela vai ter até nove horas pra tomar o banho. Então pode ser que um dia ela, “ah hoje tô com preguiça, não quero tomar banho”, mas as vezes vai. Porque as vezes tá, já passou a noite

toda de fralda, a maioria das dependentes usam fralda. Então, já tá precisando de fazer a limpeza, a assepsia, aí vai, mas, tenta-se, é, é, avaliar a situação de cada uma, né?! Tenta-se, a orientação que as cuidadoras recebem, é de sempre estimular. Então mesmo no banho, as dependentes, pega, elas tentam estimular, pega, entrega o sabonete pra elas, pede pra elas passar, aonde elas consegui, a fazer a higiene do jeito que elas conseguem, ajudar no cabelo. Então, tenta-se, tem boa parte aqui que tem hemiparesia, então, já não consegue mexer o braço bem, ou virar né, virar já é difícil, precisa de ajuda. E isso, assim, é, as que já estão nesse quadro, até aceita melhor, as outras que, que tão pra, chegando nesse quadro né, as que seriam grau II, que com alguma dependência, cê ainda percebe muita resistência. Então, as vezes você percebe, que a idosa, num tá com higiene boa, não aceita, a cuidadora ajudar, né, que é, alguém vai deflagrar meu corpo, né?! Então isso, isso dificulta um pouco, as vezes elas não entendem, porque as vezes, não quer a cuidadora chegue, é, as vezes perto "oh vou te ajudar no banho", mas não com violência, né?! De violência que eu digo, é a violência mesmo de obrigar né "ah cê vai agora, eu vou te ajudar". Só se for alguma diarreia e tá deitada e suja se toda, obvio que vai pro banho sem, querendo ou não né, mas tenta-se sempre fazer falar. Quando as cuidadoras, vem conversar com a gente fala, explica pra idosa o que cê vai fazer, o porquê cê vai fazer, porque que tá de errado, o que, que precisa melhorar e assim elas fazem, né. Tem, tem assim algumas vezes a gente percebe que alguma coisa foge da, desse ideal, porque as vezes, elas têm, elas têm tanta coisa pra fazer, que eu acho, que eu imagino, eu que tô percebendo, nunca falaram que, que "ah não, deixa eu fazer", "porque eu tenho mais cinco pra dar banho", né. Então, eu imagino que isso aconteça, em algum momento, mas o que a gente tenta falar, é dentro das orientações, também não são todas né. Por exemplo as cuidadoras, que, que trazem isso pra gente, mais as que trazem, a gente sempre tenta orientar e algumas já tem isso muito certo com elas, né. De sempre estimular a autonomia, a independência, mesmo que seja, só pegar a bucha e ficar segurando, mas pelo menos a sensação, né, "eu tô me limpando". Então assim, vai ter um pouquinho de cada coisa, mais tem, dentro do das idosas, que já tão em grau de demência, chega uma hora que que elas não falam muito, a não ser assim, tem alguma que fala: "ah eu tô com o corpo doendo" mesmo sendo demente, tá mesmo uai, tá numa cadeira o dia todo, como é que não tá, né?! Porque fazer esse movimento aqui, pra algumas já é difícil, imagina ficar na mesma posição muito tempo. Então nesta hora, a gente percebe que elas é, tão falando um pouquinho, né, do que elas tão sentindo que tão vivendo. E são uma demanda, né, ou seja, elas tão com o corpo doendo, pra quem quer entender, eu preciso mudar de posição.

Eu posso deitar um pouco, claro que por ser uma instituição, tem as rotinas né, a casa, cês podem perceber, que a maioria das idosas não ficam no leito, a não ser que estejam com alguma indisposição, né?! Tá sentindo mal, aí sim a gente deixa no leito, pra evitar escaras, porque a pele é tão frágil, que se deixar pouco tempo, que pra gente é pouco, pra elas pode já ser o suficiente, pra da as ulceras, né, de pressão. Mas é, é dentro do possível, algumas são movimentadas antes da hora, né. Nós temos idosa que não gosta de jantar, a gente sabe que o não jantar, é, cê vai ficar, apesar de ter as oito da noite um leite com biscoito, apesar de ser muito leve, passar a noite toda só com esse leite e biscoito pode ser pouco, né?! Mas tenta-se respeitar, todo dia é oferecido pra essa pessoa, aí ela fala: "não", é respeitado, né. Mas porque, a história dela, já diz, de passar fome, de ficar muito tempo sem se alimentar, né, mas é oferecido, a escolha é dela. Essa é dependente, mas ainda tem um grau de lucidez que, que como resposta deixa a equipe mais tranquila, né, de que é, de que, de que, não vai ser ruim pra ela. Tem outras que, que não gostam de ficar na área social, né, cê tenta respeitar, né, mas cê dá opções, né, é, "vai um pouquinho", "olha de longe", é, "o que, que cê gosta?", "o que cê gostaria que tivesse aqui?" Até nisso, as vezes é difícil, né, porque elas não tão acostumadas a ninguém perguntar a elas, não são todas, óbvio, mas esse, essa convivência, né, de longo prazo, já faz, elas já estabelecem nelas, uma confiança que elas chegam num cantim e falar: "oh é eu queria que tivesse uma oficina disso, disso e disso", aí a gente fala "oh a gente vai tentar". Porque as vezes, tem uma idosa, que tá com perda de visão, mais que ela quer, porque quer, fazer oficina de artesanato e não tá enxergando. Então assim, cê vai estimular o ponto fraco dela, naquele momento, né, cê tenta oferecer outra coisa, ela é resistente? É resistente, mas cê vai tentando, cê vai estimulando, cê vai apresentando outras possibilidades né, porque é essa propriamente dito, fazer uma coisa que ela não vai dar conta mais, né?! Porque já não tá vendo, não tá enxergando, mais aí da, vai ter alguma questão que vai aparecer disso né, no mínimo uma frustração. Mas no geral, a gente tenta isso, respeitar, perguntar mesmo pras não lucidas, sempre pergunto. Eu pelo menos sempre pergunto, "a senhora gostaria disso?", "a senhora quer ir em tal lugar?", as vezes elas falam, porque mesmo as não lucidas, as vezes tem uns momentos de lucidez, que a resposta é congruente com a pergunta, né?! Aí a gente tenta respeitar, né, mas é, é, é o dia a dia é a confiança delas, saber que não vai magoar né, então algumas por exemplo quando eu vou fazer uma atividade fala: "eu vou só porque é você" eu falo: "não", "a senhora não quer ir porquê? Aí tem dia que eu não levo, tem dia que eu deixo, aí eu sinto isso que, às vezes, elas falam assim, né. Então vai só porque sou eu, ou porque é alguém, outra pessoa, não é por isso,

aí a gente tenta entender o porquê tá acontecendo, que a senhora não quer participar. Sendo que, tem outras que acham, que poderiam ter muito mais pessoas, dentro da casa né?! A gente vai conhecendo, entendendo e dando espaço, mas repito, é algumas, é, tem um certo limite de expor a sua opinião. Algumas vão, mesmo se o resultado não for o que elas gostam e depois, às vezes gera um problema, insatisfação, é, algumas preferem não opinar, até lucidas, preferem não opinar, ou tem um pouquinho de cada coisa.
