# Supplementary material for: Care for older adults with disabilities in Long Term Care Facility
Source: Rev Bras Enferm. 2023 Dec 8;76(Suppl 2):e20220767. doi: 10.1590/0034-7167-2022-0767 (PMC10704689; doi:10.1590/0034-7167-2022-0767)
Supplement: 0034-7167-reben-76-s2-e20220767-suppl20 [file 0034-7167-reben-76-s2-e20220767-suppl20.pdf]

EP 20

1) Pesquisador 2: **Como é, pra você, trabalhar em uma ILPI?**

EP 20: Eu gosto, eu gosto de trabalhar com idoso, já tentei hospital, não gostei. Fui ali, pra Santa Casa, né, mas num gostei não, prefiro IPLI.

*Pesquisador 1: Por gostar dos idosos?

EP 20: Gosto, gosto.

*Pesquisador 1: Do trabalho?

EP 20: Gosto da rotina, gosto, tanto é, que eu já arrumei até outro, na mesma, rs, em outra clínica.

*Pesquisador 2: Cê trabalha em duas casas?

EP 20: Em duas casas, é.

*Pesquisador 2: E aqui foi sua primeira experiencia, com idoso?

EP 20: Foi, foi.

2) Pesquisador 2: **Me fale um pouco sobre seu relacionamento com os idosos que residem aqui.**

EP 20: Ah eu me do bem com eles, nunca tive desentendimento, nunca tive, não tive, não tiveram reclamação de mim, né?! Pelo menos que eu saiba, nunca chegou em mim, tem seis anos e assim, considero aqui a segunda casa. Porque fico aqui, e lá em casa, né, e eu já trabalhei aqui de dia, aí que a gente convive mais ainda com elas, né?! E a noite, a maioria tá dormindo, mais mesmo assim tenho um bom convívio com elas.

3) Pesquisador 2: **Qual a sua percepção sobre a relação dos idosos institucionalizados com seus familiares e amigos?**

EP 20: A maioria aqui não tem familiares, os pouco que tem, se dão bem, igual agora a pouco, cês viram, né?! O da dona Elvira, tá sempre presente, agora a maioria não tem parente, tem amigos, né, tem vizinhos, que de vez em quando vem. Da Guedes mesmo, vem, as amigas dela, o pessoal da igreja. Que tem umas que são evangélica, apesar que aqui, né, o pessoal é católico, mais isso não tem nada a ver. Mais aí eles vem visitar elas.

*Pesquisador 1: Cê acha que elas recebem essas visitas com frequência ou não?

EP 20: Olha, quando eu tava de dia, era mais ou menos, rs. Quem recebe mais, é igual a dona Elvira, dona Altina recebia também, do sobrinho, mais ele já faleceu. Mais assim, que tem mais frequência mesmo é dona Elvira, a Yara. Agora esses que tão vindo agora da prefeitura, que, né, essas já estavam aqui, né antes, mais eu acho que é pouco, por eles não terem familiares, né?! Mais é amigo que vem.

*Pesquisador 1: Cê acha que a casa estimula, essa vinda de amigos e familiares com frequência?

EP 20: Ah isso eu não vou te responder, porque na época né, que eu tava de dia era mais ou menos, rs, tô sendo sincera, né, agora, não sei como tá agora. Porque a gente a noite, tem coisa que durante o dia, não pega, não percebe, não vê.

*Pesquisador 1: Tem quanto tempo que cê trabalha a noite?

EP 20: Tem três anos, trabalhei três anos de dia e três anos a noite.

4) Pesquisador 2: **Você considera que os idosos dessa ILPI têm condições de tomar decisões sobre as coisas que precisam fazer em seu dia-a-dia? Por quê?**

EP 20: Nem todos.

*Pesquisador 1: Por quê?

EP 20: Uai, acho que são poucos que tem aqui, mais as que são conscientes a Nercina, a Yara, umas cinco, só.

*Pesquisador 1: E só elas que decidem o que elas fazem? As outras não?

EP 20: Uai tem umas que não tem condição de responder, né?! Aí como se diz, vamos supor, vai ter uma atividade, não tem como saber, você, a gente pergunta, "fulana você quer ir?" pelo menos é assim, era, né no dia quando eu estava. Hoje vai ter o terço, "você quer rezar?" "vão", "eu vou", "não eu não quero". Então a gente não colocava ela lá dentro, ela não quer e as que responde por si, elas mesmo falam, né?! A gente só fala: "hoje vai ter isso essa atividade", "hoje vai ter uma comemoração de aniversariante", "hoje vai ter visita". Convida elas pra participar, agora tem umas que as vezes quer ir, aí a gente respeita.

*Pesquisador 1: Mais as que não querem ir, as vezes podem ser, não, não necessariamente precisam ser independentes, né?

EP 20: Isso.

*Pesquisador: Pode ser uma cadeirante ....

EP 20: É mais a gente pergunta pra todos, igual “oh Pretinha”, é a Maria Alves, “cê que participar?”, “não, hoje eu não quero não”, e a gente deixa, ela não quer ir. As vezes a gente tenta, “não vai ser legal” e tal, “vamos lá participar”, “é pra cantar”, tenta incentivar, pelo menos eu fazia isso né, rs. Agora eu não sei como que está durante o dia, tô respondendo por mim, eu fazia isso.

*Pesquisador 1: Cê acha que todas tem condição, assim, de vocês perguntarem e elas responderem?

EP 20: Não, não, nem todas, igual a Bel, a xô vê a Eugenia, merma hora que ela que ela não que, entendeu? A Dora, as vezes ela quer, as vezes ela não quer também, mais são poucas.

*Pesquisador 1: Cê acha que a maioria consegue responder?

EP 20: Consegue, consegue.

*Pesquisador 1: Se perguntar?

EP 20: É, porque se não perguntar elas não vão saber o que está acontecendo, vão supor, tá todo mundo sentado assistindo televisão. Chegou o pessoal, uma visita aqui no refeitório, se você não levar e não perguntar. A visita vai entrar e sair e elas não vão saber ué, ainda mais as cadeirante, que cê coloca ali, se deixar fica ali. Agora, aquelas que anda pra lá e pra cá, pode, né, não passa despercebido, as vezes tão no quarto.
