# Supplementary material for: Care for older adults with disabilities in Long Term Care Facility
Source: Rev Bras Enferm. 2023 Dec 8;76(Suppl 2):e20220767. doi: 10.1590/0034-7167-2022-0767 (PMC10704689; doi:10.1590/0034-7167-2022-0767)
Supplement: 0034-7167-reben-76-s2-e20220767-suppl21 [file 0034-7167-reben-76-s2-e20220767-suppl21.pdf]

EP 21

1) Pesquisador 2: **Como é, pra você, trabalhar em uma ILPI?**

EP 21: É pra mim, normal, té gosto, já acostumei, aí não me vejo assim, trabalhando em outra área, que não se essa, já tô até acostumada já, tem as dificuldades como todos os emprego tem, né?! Mais dá pra levar.

*Pesquisador 2: E aqui foi seu primeiro emprego?

EP 21: Não, assim de carteira assinada é o segundo.

*Pesquisador 2: De cuidador de idoso?

EP 21: Não, eu trabalhei em casa de família um anos e quatro meses, aí sai e vim pra cá.

*Pesquisador 1: De cuidador de idosos, é o primeiro, né?

EP 21: É o primeiro, uhum, também nem deu pra sair né, oito ano num lugar só, rs. A maioria chega aqui e fica dois meses e vai embora, depois que eu tô aqui gente, já passou tanta gente, chega fica dois mês, três mês não dura nem um ano, dois ano e eu já tô me sentindo aposentando aqui, rs.

*Pesquisador 2: E porque que cê acha que as pessoas não ficam?

EP 21: Ah não sei, é porque as pessoas chegam aqui com o objetivo acho que de uma coisa e ela chega aqui é outra coisa, eu acho assim né, não sei, porque tipo assim ela chega aqui, sabe o que tem que fazer, aí com o mês que tá aqui, ou quinze dias, já começa reclamar, "ah não era isso que eu queria, tá pesado", não é assim, entra sabendo que vai trabalhar mesmo, trabalhar com idoso é viver nos altos e baixos. Eu, eu gosto, não sei os outros, os outros reclamam, ou não reclamo não, rs.

2) Pesquisador: **Me fale um pouco sobre seu relacionamento com os idosos que residem aqui.**

EP 21: Ah, eu não tenho problema com nenhum, pra falar a verdade pra você, todos eu chego e me comprimenta muito bem, me trato bem, eu trato todo mundo bem, gosto de tratar com carinho, com respeito, na hora de você brincar, é hora de você brincar, na hora de falar sério é hora de falar sério, porque idoso, cê tem que, tem umas que cê tem que chegar e falar sério, porque se não, aí tem muitas aqui também que cê vê carinhoso, tem

outras que cê vê que são mais agitado, mais nervoso, mais é tranquilo, eu relaciono muito bem com eles graças a Deus.

3) Pesquisador 2: **Qual a sua percepção sobre a relação dos idosos institucionalizados com seus familiares e amigos?**

EP 21: Aí, como eu trabalho a noite, eu num vejo muito essas coisas, se vem família se não vem, entendeu? A gente que trabalha a noite, a gente chega nesse horário vai embora seis horas da manhã, então a gente num vê, mais eu acho que os que vem aqui acho que é bem, né, não vejo reclamação não.

*Pesquisador 2:E eles falam alguma coisa, os idosos?

EP 21: Não, a Maria quando a irmã dela vai vim ela já começa a falar desda noite “a minha irmã vem amanhã, a minha irmã vem amanhã” fica feliz da vida, fala pra todo mundo que a irmã dela vem, mais os outros, num é muito de comentar, o filho da dona Elvira sempre eu vejo, sempre a gente chega ele fica até mais tarde com ela, mais as outras a gente não, as vezes gente nem conhece familiar deles.

*Pesquisador 2: E cê sempre trabalhou aqui a noite?

EP 21: Eu trabalhei muitos anos atrás, já tem muito tempo, trabalhei uns dois ano e meio durante o dia, aí fui pra noite, então a maioria dessas idosas que eu trabalhava durante o dia já faleceu, não tem mais, tem poucas, agora essas que entrou agora, tudo eu já tava a noite, aí não tem muito contato familiar.

4) Pesquisador 2: **Você considera que os idosos dessa ILPI têm condições de tomar decisões sobre as coisas que precisam fazer em seu dia-a-dia? Por quê?**

EP 21: Ah nem todas, algumas sim, igual a Nercina que ela é lucida, né?! A Nercina, a Gilberta, a Yara, a maioria não, que a maioria não tem noção de nada, né, mais as que tá lúcida, eu acho que sim.

*Pesquisador 1: Então é de acordo com a lucidez delas?

EP 21: Eu acho que sim, pelo menos o que eles falam aqui, porque essas que é lucida, elas têm acesso livre pra sair, entrar a hora que elas quer, já as outras acamadas não.

*Pesquisador 1: Mesmo pra coisas simples, como comer, tomar banho, escolher o que quer fazer durante o dia.

EP 21: Não, acho que pra isso assim, elas não tem muito não, porque ... eu não sei, não vou falar, que igual eu falei pro cê a noite tipo assim se ela falar “eu não vou usar uma fralda” a gente não vai lá colocar, entendeu?! Fica sem a fralda, se você não quer lanchar, então a gente não vai forçar, mais as coisas assim, mais outras que é lúcida mesmo tem mais autoridade, né, de que as tão mais acamada.
